# The intracellular subdomain of the volume-regulated anion channel subunit LRRC8A is a hotspot of channel activation

**DOI:** 10.1038/s41467-026-77507-y

**Published:** 2026-09-17

**Authors:** Sara Bertelli, Lu Wang, Malte Klüssendorf, Paola Gavazzo, Michael Pusch, Raimund Dutzler, Tobias Stauber

**Affiliations:** 1 Biophysics Institute, National Research Council, Genova, Italy; 2 Department of Biochemistry, University of Zurich, Zurich, Switzerland; 3 Institute for Molecular Medicine, MSH Medical School Hamburg, Hamburg, Germany; 4 Present Address: Institute of Biology, Cellular Biophysics, Humboldt Universität zu Berlin, Berlin, Germany

**Keywords:** Chloride channels, Cryoelectron microscopy, Permeation and transport, Biological fluorescence, Voltage clamp

## Abstract

Volume-regulated anion channels (VRACs), formed by heteromers of LRRC8 subunits, mediate the transport of anions and organic osmolytes in numerous physiological processes. Although their gating mechanism remains poorly understood, conformational rearrangements of the cytosolic leucine-rich repeat domains (LRRDs) have been implicated as a key step towards activation. Here, we identify the intracellular subdomain (ISD) of the obligatory LRRC8A as a key element coupling LRRD motion to pore opening. Substitution of leucine 402 with tryptophan (L402W) within a hydrophobic pocket of the ISD rendered LRRC8A homomers constitutively open, independent of osmotic stimulation or pharmacological modulation. Mutation of the nearby tryptophan 168 to leucine (W168L) also enhanced channel activity whereas the combination of both mutations (W168L-L402W) partly attenuated the hyperactive phenotype of L402W. FRET measurements revealed increased LRRD flexibility of L402W, which was reduced in W168L-L402W or by coexpression with an inhibiting sybody. The cryo-EM structure of L402W showed pronounced ISD rearrangements and outward tilting of the LRRDs, while the double mutant W168L-L402W contains features explaining its intermediate phenotype. Together, these data identify the ISD as a regulatory hub that transmits conformational changes from the LRRDs to the transmembrane gate of VRAC.

## Abstract (web summary)

Ion channels of the LRRC8 family are modular proteins consisting of a transmembrane pore and cytoplasmic regulatory domains. Here the authors combine structural and functional data to define the role of the intracellular sub-domain (ISD) of the obligatory LRRC8A subunit for channel activation.

## Introduction

Members of the LRRC8 protein family form large-pore channels that were shown to conduct a wide range of substrates ranging from halides to larger organic metabolites including taurine, amino acids and nucleotides as well as xenobiotics such as cisplatin and blasticidin^1–3^. In cells, these channels form heteromers composed of the essential LRRC8A subunit and its paralogues LRRC8B–E, with the subunit composition determining substrate specificity^4–6^.

LRRC8 channels are activated by osmotic cell swelling and are therefore referred to as volume-regulated anion channels (VRACs)^1,7^. The efflux of chloride and organic osmolytes through VRACs supports regulatory volume decrease. Channels of the same family can also be activated by isovolumic stimuli and contribute to diverse physiological processes by conducting signaling molecules such as excitatory amino acids, ATP, and cGAMP, regulatory molecules like taurine and glutathione or by modulating the membrane potential^1–3,8–22^. However, the activation mechanism remains unclear at both the cellular and structural levels^23–26^.

The five LRRC8 isoforms share a common organization containing four transmembrane helices in their N-terminal region and a large cytosolic part harboring multiple leucine-rich repeats (LRRs)^4,5,27,28^. Cryo-EM structures of LRRC8A homomers have defined the overall architecture of the channel. These form hexameric assemblies composed of a pore domain (PD) and horseshoe-shaped leucine-rich repeat domains (LRRDs) located in the cytoplasm^29–36^. The PD consists of three distinct regions: a tightly packed extracellular sub-domain (ESD) that likely serves as a selectivity filter, a transmembrane sub-domain formed by four helices per subunit and an intracellular sub-domain (ISD) that consists of an extended loop connecting TM2 and 3 and the region following TM4^29^. Except for a flexible region in the TM2-3 loop, both parts of the ISD form a compact unit that is critical for subcellular trafficking^29,37,38^. The ISD links the PD to the LRRD and thus occupies a strategic position between the two structural components of the protein. In most known structures of LRRC8 proteins, the proteins assemble as hexamers with the PD exhibiting six-fold (C6) symmetry, whereas the LRRDs show pronounced structural heterogeneity reflecting their higher mobility. In case of LRRC8A, which forms anion selective channels residing in a stable closed conformation^29,39,40^, pairs of LRRDs rearrange to maximize their interaction yielding a trimer-of-dimer arrangement with three-fold (C3) symmetry^24–26^.

In several studies, the LRRDs have been implicated in channel activation, potentially by alteration of their inter-subunit interaction due to reduced cytosolic ionic strength during osmotic cell swelling^40^. The fusion of bulky proteins to the C-termini enhances hypotonicity-induced activity and even causes basal activity under isotonic conditions^41^. FRET measurements between fluorescent proteins fused to the C-termini of LRRC8 proteins show reversible decreases during activation, consistent with LRRD movement^42^. In addition, binding of synthetic nanobodies (sybodies) to the LRRDs allosterically modulates channel activity: sybodies binding to the concave inner surface promote mobility and channel activation, while those stabilizing the outer surface inhibit the channel^43^. A similar inhibitory effect has been reported for puromycin-sensitive aminopeptidase^44^. Finally, the structures of LRRC8 heteromers consisting of A/C and A/D subunits show a pronounced destabilization of the LRRDs, reflecting their enhanced activation properties^34,35,45^.

How the LRRD movement is mechanically coupled to pore gating remains unknown, but this link likely involves the ISD connecting the LRRD to the transmembrane domain (TMD). Variants of LRRC8C identified in patients with a multisystem disorder cause amino acid substitutions or truncations within the ISD and lead to constitutive activation of LRRC8A/LRRC8C heteromers^46^. The structural analysis in these cases revealed increased LRRD flexibility in mutant LRRC8C homomers, further supporting a role for LRRD dynamics in gating^46^.

In this study, we probe the same region as found in LRRC8C disease-causing mutants to dissect the role of the ISD of the obligatory LRRC8A subunit in transmitting conformational changes from the LRRDs to the transmembrane region. We identify a mutation that renders LRRC8A homomers constitutively open, an effect partly attenuated by introducing an opposing substitution of an interacting residue. FRET measurements indicate enhanced LRRD flexibility in the open mutant, which is reduced by the attenuating mutation or by an inhibitory sybody. Cryo-EM analysis of the open mutant confirms increased LRRD mobility and reveals marked ISD rearrangements, whereas the double mutant shows features that can explain its partial phenotype. Together, these findings identify the ISD as a critical coupling element that links LRRD movement to pore gating in LRRC8 channels.

## Results

### Identification of a hotspot of channel activation

**Fig. 1:**
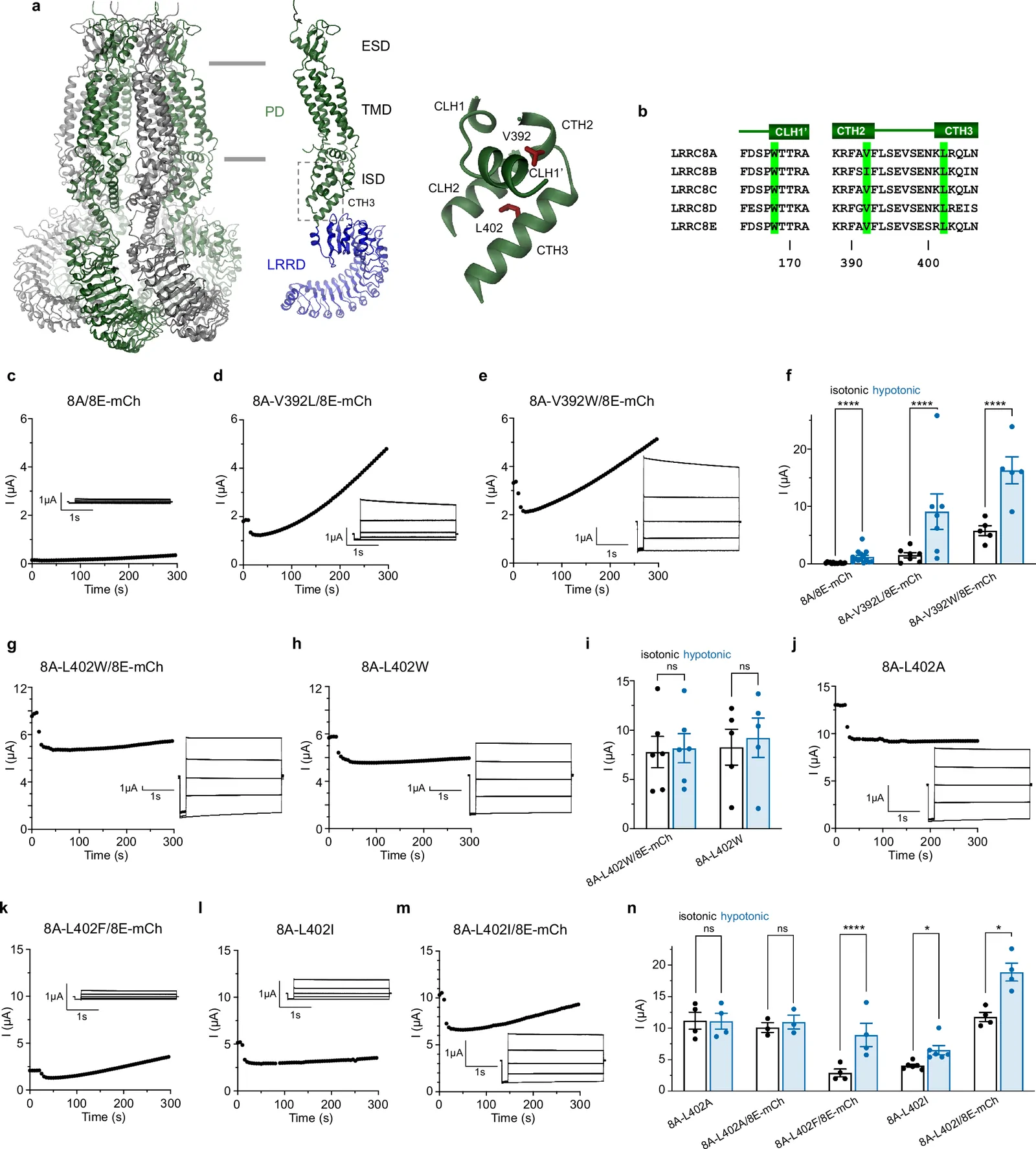
Activating mutations in LRRC8A. **a** Ribbon representation of the LRRC8A homomer (PDBID 7P5V). Left, view of the hexameric channel with membrane boundaries indicated, center, single subunit with PD and LRRD shown in unique colors and region of the ISD framed by a box, and right, zoom into the ISD with residues mutated in this study shown as sticks. **b** Sequence alignment of a region of the ISD of the five human LRRC8 subunits with mutated residues highlighted. Numbers refer to the LRRC8A sequence. **c–e** Time-course current response of heteromeric LRRC8 channels composed of a LRRC8E-mCh fusion protein and constructs of LRRC8A, expressed in *X. laevis* oocytes after exposure to hypotonic solution (t = 0), recorded by two-electrode voltage clamp electrophysiology. **c** LRRC8A WT. **d** V392L. **e** V392W. **f** Currents at isotonic and after application of hypotonic solutions of constructs shown in (**c**–**e**). **g** Current response of heteromeric channels composed of an LRRC8E-mCh fusion protein and the LRRC8A mutant L402W, and **h** of homomeric L402W. **i** Currents at isotonic (*t* = 0) and after application of hypotonic solutions (*t* = 250 s) of constructs shown in (**g**, **h**). **j–m** Current response of, **j** homomeric channels of the LRRC8A mutant L402A, **k** heteromeric channels composed of a LRRC8E-mCh and the LRRC8A mutant L402F, **l** homomeric channels of the LRRC8A mutant L402I, **m** heteromeric channel of the same construct with LRRC8E-mCh. **c–e**,** g**,** h**,** j–m**, Currents were measured as repetitive pulses to +60 mV applied at 0.2 Hz of representative cells injected with the indicated constructs in response to a hypotonic stimulus (the initial drop of currents is due to the lower chloride concentration in the hypotonic solution). Insets show current traces of the same oocytes in response to the IV-pulse protocol. **f**, **i**, **n** Mean current amplitudes measured at +60 mV under isotonic conditions (*t* = 0) and after ~280 s of hypotonic stimulation for the constructs shown in the corresponding panels. Individual measurements are shown as dots, and bars indicate mean ± s.e.m. Currents from the same oocyte before and after hypotonic stimulation were compared using a two-tailed paired ratio t-test; **n** refers to the number of recorded oocytes. In **f**, significant activation was observed for 8A/8E-mCh (*n* = 14, *P* < 0.0001), 8A-V392L/8E-mCh (*n* = 7, *P* < 0.0001) and 8A-V392W/8E-mCh (*n* = 5, *P* < 0.0001). In **i**, no significant change in current amplitude was detected for either 8A-L402W/8E (*n* = 6, *P* = 0.0707) or homomeric 8A-L402W channels (*n* = 5, *P* = 0.2138). In **n**, hypotonic stimulation did not significantly affect currents of 8A-L402A (*n* = 4, *P* = 0.8387) or 8A-L402A/8E-mCh (*n* = 3, *P* = 0.0802), whereas significant activation was observed for 8A-L402F/8E-mCh (*n* = 4, *P* < 0.0001), 8A-L402I (*n* = 6, *P* = 0.0151) and 8A-L402I/8E-mCh channels (*n* = 4, *P* = 0.0297).

A previous study investigating disease variants in LRRC8C (short 8C) has identified the intracellular sub-domain (ISD) of this subunit as a regulator of channel activity^46^. Two variants in this region that both cause a strong gain-of-function effect were found to be associated with a severe multisystem developmental disorder, including vascular and skeletal abnormalities^46^. Since one variant, 8C-V390L, concerns a conservative amino acid replacement located at the C-terminal end of the ISD helix CTH2, we have investigated whether the equivalent mutation in the conserved position of LRRC8A (short 8A) exerts a similar phenotype (Fig. 1a, b). As the overexpression of mutations with strongly activating properties was poorly tolerated by HEK293 cells, we have instead relied on their expression in *Xenopus laevis* oocytes by co-injection of untagged 8A RNA and mCherry-tagged 8E RNA (8E-mCh). The fluorescent tag in the E subunit induces a detectable increase in the constitutive activity of the channel, which is, however, small compared with the effect observed when the fluorescent tag is present on both subunits^4,41^ (Fig. 1c). Consequently, we do not expect the presence of the fluorescent protein to strongly bias the results. The mutant 8A-V392L co-expressed with 8E-mCh gives rise to a gain-of-function effect compared to WT 8 A/8E-mCh, showing larger constitutive currents while still being further activated by hypotonicity (Fig. 1c, d). For the same residue, the substitution by the bulkier Trp (8A-V392W) had an even stronger activating effect (Fig. 1e, f).

We next constructed the mutant 8A-L402W (Fig. 1a), inspired by the second disease-related variant of 8C that carries a frameshift mutation at the corresponding position, resulting in a subunit lacking the LRRD. In 8C, this variant also strongly enhances activation^46^. This residue, which is part of the same hydrophobic core of the ISD as Val 392, is located 10 residues downstream on the ensuing helix CTH3 connecting to the LRRD (Fig. 1a, b). 8A-L402W co-expressed with 8E-mCh induces very high constitutive currents that were insensitive to hypotonicity (Fig. 1g). The large currents of 8A-L402W/8E-mCh, which were observed already one day after mRNA injection, led us to express the mutant construct as homomeric assembly without 8E-mCh, resulting in similar large constitutive currents obtained in the same time range (Fig. 1h), with 8A-L402W expressed at similar levels as WT 8A (Supplementary Fig. 1a). As for the heteromeric A/E channels carrying the mutation, these currents were also insensitive to hypotonicity (Fig. 1g–i), hypertonicity (Supplementary Fig. 1b) and the VRAC inhibitors carbenoxolone (CBX) and DCPIB (Supplementary Fig. 1c, d). The recorded currents are mostly carried by Cl^-^ because they are strongly reduced when this small anion was replaced by the larger glutamate or when NaCl was removed from the extracellular solution (Supplementary Fig. 1e). The slightly less pronounced reduction in glutamate could indicate a residual permeability of this amino acid (Supplementary Fig. 1e, f). In addition, the absence of effect upon replacement of sodium by the large cation NMDG provides further evidence for the high anion selectivity (Supplementary Fig. 1e).

To probe whether the size of the Trp sidechain in the mutant 8A-L402W is essential to confer the strong activation, we tested other mutations at this position with different side-chain volumes (Fig. 1j–n). Surprisingly, the truncation of the side-chain in the mutant 8A-L402A behaved very similarly to its expansion in the mutant 8A-L402W. The injection of 8A-L402A on its own, or together with 8E-mCh, gave rise to large constitutive currents that were insensitive to either hypotonicity or hypertonicity and the addition of CBX, similar to 8A-L402W (Fig. 1g–j, n and Supplementary Fig. 1b, c). In contrast, 8A-L402F yielded no detectable activity when expressed on its own, whereas its co-expression with 8E-mCh resulted in enhanced constitutive currents, which were further stimulated by hypotonicity (Fig. 1k, n) and inhibited by hypertonicity and the addition of CBX (Supplementary Fig. 1b, c). Finally, we tested the conservative mutation L402I. Remarkably, 8A-L402I yielded elevated currents when expressed on its own, which were modestly sensitive to hypotonicity (Fig. 1l, n). When co-expressed with 8E-mCh, constitutive and hypotonicity-induced currents further increased (Fig. 1m, n), illustrating that a small perturbation of the side-chain volume in this position is sufficient to alter the activation properties of the channel. Thus, substituting Leu 402 with either a very bulky residue (Trp) or a very small residue (Ala) leads to strong activation even without 8E subunits, whereas substitution by Phe or Ile has a detectable, yet less pronounced effect, suggesting that the hydrophobic pocket burying the Leu sidechain was optimized for its size and that any perturbation in this position destabilizes the closed channel conformation.

### Compensatory effect of a double mutation

**Fig. 2:**
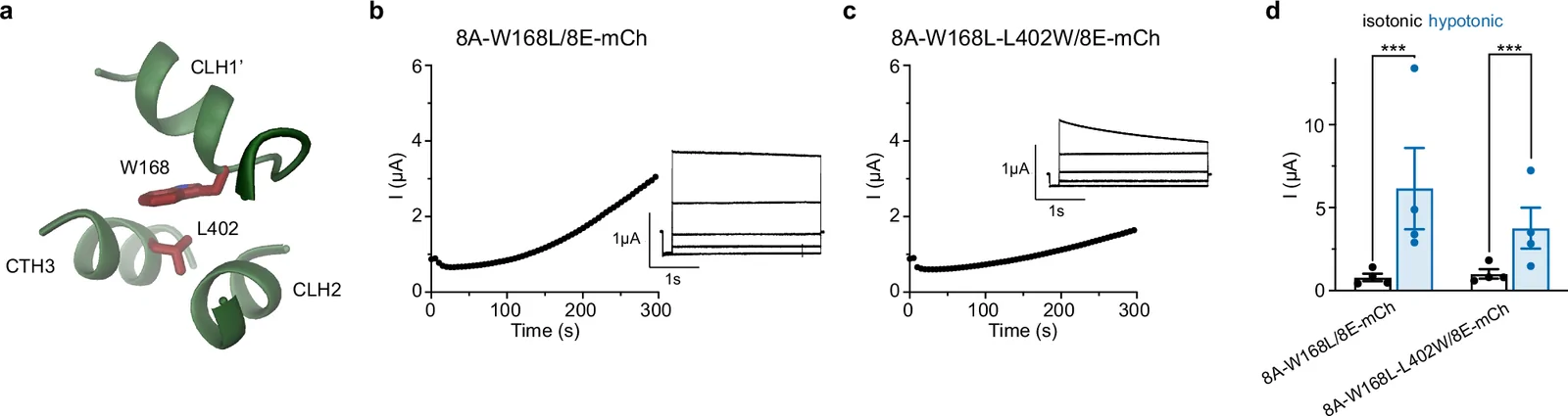
Mutation W168L restores gating in the context of the L402W mutation in heteromeric 8 A/8E channels. **a** Ribbon representation of a part of the ISD of LRRC8A showing the relationship between L402 and W168. **b**,** c** Current response to a repetitive pulse to +60 mV applied at 0.2 Hz of representative *X. laevis* oocytes injected with **b** LRRC8E-mCh and the LRRC8A mutant W168L, and **c** LRRC8E-mCh and the LRRC8A double mutant W168L-L402W, to a hypotonic stimulus. Insets show current traces of the same oocytes in response to the IV-pulse protocol. **d** Mean current amplitudes measured at +60 mV under isotonic conditions (*t* = 0) and after ~280 s of hypotonic stimulation for the constructs shown in (**b**, **c**). Individual measurements are shown as dots, and bars indicate mean ± s.e.m. Currents from the same oocyte before and after hypotonic stimulation were compared using a two-tailed paired ratio t-test. Significant activation was observed for both 8A-W168L/8E-mCh (*n* = 4, *P* = 0.0003) and 8A-W168L-L402W/8E-mCh (*n* = 4, *P* = 0.0021). n refers to the number of recorded oocyes.

Upon inspection of the structure of homomeric 8A, we noted that Leu 402 is close to residues of the linker connecting the membrane-spanning α-helices TM2 and 3. In particular, it appears to be in direct contact with Trp 168 on CLH1’ (Fig. 2a). To functionally explore a possible interaction between residues located on CTH3 and CLH1, we first investigated the mutant 8A-W168L co-expressed with 8E-mCh. The mutant resulted in increased constitutive and hypotonicity-induced currents compared to WT 8A/8E-mCh that were sensitive to hypertonicity and the addition of CBX (Fig. 2b and Supplementary Fig. 1b, c). No currents could be detected when 8A-W168L was injected on its own, suggesting that the consequences of the mutation on activation are much less severe than those of 8A-L402W (Supplementary Fig. 2a, b).

We next tested whether a combination of the mutants 8A-W168L and 8A-L402W could result in a mutual compensation and reduce the drastic effect of the latter. Indeed, in contrast to the single mutant L402W, the double mutant 8A-W168L-L402W expressed on its own did not yield any constitutive currents or signs of activation upon changing to hypotonic conditions (Supplementary Fig. 2c, d), while the co-expression of the double mutant 8A-W168L-L402W with 8E-mCh showed pronounced, yet much smaller constitutive currents than 8A-L402W or 8A-L402W co-expressed with 8E-mCh, while currents remained sensitive to hypotonicity (Fig. 2c, d). Both 8A-W168L/8E-mCh and 8A-W168L-L402W/8E-mCh were sensitive to CBX (Supplementary Fig. 1c).

Our studies thus demonstrate that the strong activating effect of a mutation in the terminal helix of the ISD can be reduced by a compensatory mutation with an interacting residue located on the same sub-domain that is distant in sequence. Taken together, our functional investigation of residues that are part of a hydrophobic core of the ISD and whose mutations show a strong activating phenotype illustrates the importance of this region for controlling channel gating.

### Conformational properties of LRR domains in mutant channels revealed by FRET

Our electrophysiology studies have identified a regulatory hotspot located in the ISD of LRRC8A where mutations exert a strong effect on channel activation (Figs. 1 and 2). To test whether the same mutations have caused a rearrangement of the attached LRRDs, we monitored Förster resonance energy transfer (FRET) between fluorescent proteins fused to the C-termini of LRRC8A and its corresponding mutants. Intra-complex FRET within heteromeric LRRC8 channels and homomeric assemblies of LRRC8A was previously shown to decrease upon hypotonic stimulation, indicating a separation of LRRD subunits upon activation^42^. We thus co-expressed C-terminally tagged mCerulean3 (as FRET donor) and mVenus (as FRET acceptor) constructs of LRRC8A in the background of either 8A-WT or the W168L and L402W mutants in HeLa cells and probed for changes in FRET efficiency upon their exposure to hypotonic conditions.

**Fig. 3:**
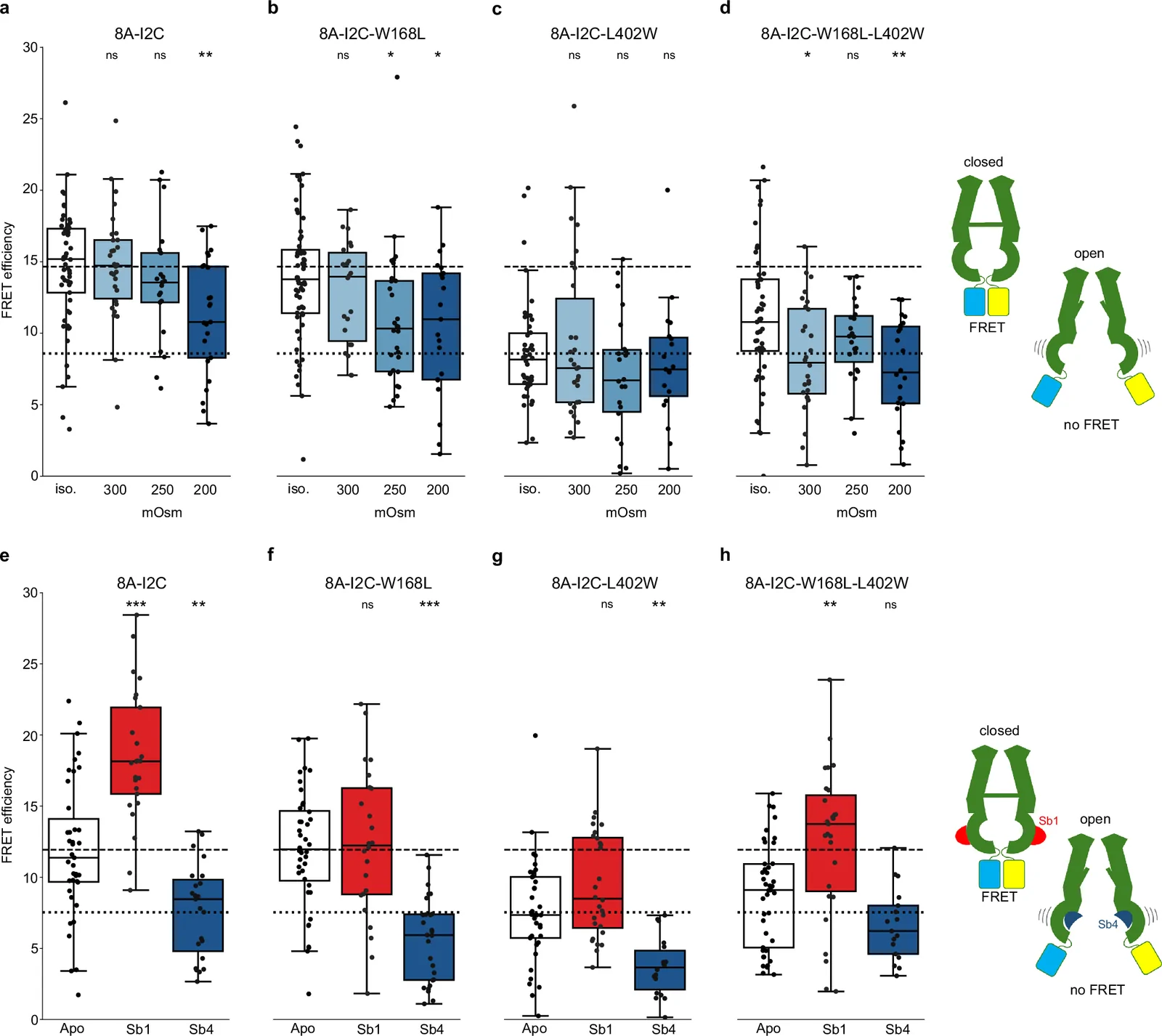
FRET experiments illustrating LRRD movement of LRRC8A constructs. FRET changes between indicated homomeric LRRC8A constructs containing fusions to fluorescent proteins upon osmotic challenges. **a–d** HeLa cells transfected with FRET pairs (donor and acceptor) for **a** LRRC8A-I2C (8A-I2C), **b** 8A-I2C-W168L, **c** 8A-I2C-L402W and **d** 8A-I2C-W168L-L402W. The cells were bathed successively in buffers with different tonicity: isotonic (iso, 340 mOsm), 300 mOsm, 250 mOsm or 200 mOsm, and images of the donor and acceptor were acquired before and after acceptor bleaching to calculate inter-subunit FRET efficiency. The dashed and dotted lines indicate the mean under isotonic conditions of 8A-I2C and 8A-I2C/L402W, respectively. *, ** significantly differ from iso, *P* < 0.05 and *P* < 0.01. One-way ANOVA followed by Tukey´s post-hoc test, comparison between the construct groups. *n* = 57, 30, 21, 25 (*P* = 0.0025) for 8A-I2C, *n* = 60, 22, 30 (*P* = 0.0147) and 21 (*P* = 0.0157) for I2C-W168L, *n* = 46, 30, 22, 19 for 8A-I2C-L402W and *n* = 51, 28 (*P* = 0.0284), 23, 22 (*P* = 0.0036) for 8A-I2C-W168L-L402W cells from 3 independent experiments each for iso, 300, 250 and 200 mOsm. **e–h** Cells expressing the same LRRC8A constructs as in (**a**–**d**) were transfected with respective FRET pairs (Apo) or together with the inhibitory sybody Sb1 or the activating sybody Sb4. Cells were bathed in isotonic buffer, and acceptor bleaching was performed as described for (**a**–**d**). The dashed and dotted lines indicate the mean of Apo of 8A-I2C and 8A-I2C-L402W, respectively. *, **, *** significantly differ from apo, *P* < 0.05, *P* < 0.01 and *P* < 0.0001. One-way ANOVA followed by Tukey´s post-hoc test, comparison between constructs group: *n* = 39 (Apo), 25 (Sb1; *P* < 0.0001), 24 (Sb4; *P* = 0.0013) cells for 8A-I2C, *n* = 40 (Apo), 24 (Sb1), 27 (Sb4; *P* < 0.0001) for 8A-I2C-W168L, *n* = 37 (Apo), 28 (Sb1), 18 (Sb4; *P* = 0.0007) cells for 8A-I2C-L402W, and *n* = 41 (Apo), 17 (Sb1; *P* = 0.0010), 17 (Sb4) cells for 8A-I2C-W168L-L402W from 3 independent experiments each. Data of individual cells are represented as points; box-plots in **a–h** show median (center line), upper and lower quartiles (box limits), 1.5x interquartile range (whiskers).

These studies turned out to be challenging for constructs with strong gain-of-function. As consequence of its constitutive activity (Fig. 1h, i), cells expressing 8A-L402W displayed compromised morphology, precluding reliable microscopy (Supplementary Fig. 3a). The effect could not be ascribed to increased cell surface expression of the mutants compared to LRRC8A-WT, as confirmed by surface biotinylation and immunostaining of an extracellular HA tag under non-permeabilizing conditions (Supplementary Fig. 4). To mitigate the described obstacle, we additionally introduced the mutations I2C or E6A into the N-terminal region of LRRC8A, which were previously shown to diminish ion conduction^33,47^. The combination of both mutants in the construct 8A-I2C-L402W, but not in 8A-E6A-L402W, improved cell morphology and allowed proper imaging (Supplementary Fig. 3b, c). The previously observed reversible hypotonicity-induced decrease in corrected FRET (cFRET) within LRRC8A complexes^42^ was preserved in the I2C mutant, indicating that the rearrangements of the LRRDs remain intact despite its impaired pore function (Supplementary Fig. 3d). We next measured the FRET efficiency using acceptor photobleaching. In isotonic buffer, where LRRC8 channels are predominantly in their closed conformation, 8A-I2C exhibited a FRET efficiency of 14.66 ± 0.55% (Fig. 3a). The W168L mutation in 8A-I2C-W168L had no effect under isotonic conditions (13.96 ± 0.59%) (Fig. 3b), whereas the strongly activating mutation L402W in 8A-I2C-L402W showed reduced FRET efficiency (8.59 ± 0.54%) (Fig. 3c), consistent with increased conformational flexibility of the LRRDs. The additional introduction of the W168L mutation in 8A-I2C-W168L-L402W partially restored FRET to intermediate levels of 11.05 ± 0.67% (Fig. 3d), consistent with the functional phenotype that is between WT and 8A-L402W observed in electrophysiology (Fig. 2c, d). The FRET efficiency of 8A-I2C decreased upon exposure to hypotonic extracellular buffer (11.16 ± 0.80% at 200 mOsm), confirming earlier observations^42^ (Fig. 3a and Supplementary Fig. 3d). A similar reduction was seen for 8A-I2C-W168L (to 10.51 ± 1.06% at 200 mOsm) (Fig. 3b), reflecting the observed increase of activity in equivalent conditions (Fig. 2b, d). In contrast, FRET in 8A-I2C-L402W was only marginally reduced further below the low level observed in isotonic conditions (7.86 ± 0.97% at 200 mOsm), consistent with the absence of additional current activation in electrophysiological recordings (Fig. 1h, i). FRET in 8A-I2C-W168L-L402W also declined under hypotonic conditions (7.42 ± 0.75% at 200 mOsm), reaching levels comparable to those of other constructs (Fig. 3d) in line with its behavior observed in electrophysiology (Fig. 2c, d).

We next tested the effect of synthetic nanobodies (sybodies) targeting the LRRDs of LRRC8A, thereby altering its activation properties^43^, on inter-subunit FRET. To this end, HeLa cells were co-transfected with mCerulean3- and mVenus-labeled LRRC8A constructs and either of two sybodies: Sb1, which inhibits LRRC8 activity by stabilizing the LRRDs in a three-fold symmetric conformation, and Sb4, which promotes activation by increasing LRRD flexibility^43^. In 8A-I2C, the co-expression with Sb1 increased FRET efficiency, consistent with LRRD stabilization, whereas Sb4 reduced FRET (Fig.  3e), in line with enhanced domain mobility as suggested by structural data^43^. Similar trends were observed for the single mutants W168L and L402W and the double mutant W168L-L402W, although absolute FRET values remained lower compared to WT (Fig. 3f–h). Notably, Sb1 failed to increase FRET in 8A-I2C-W168L, suggesting it could not further stabilize the LRRDs in this background (Fig. 3f). While Sb1 showed a trend towards increased FRET in 8A-I2C-L402W and 8A-I2C-W168L-L402W, this effect was not statistically significant and did not reach levels observed with WT 8A-I2C upon addition of the same sybody (Fig. 3g, h).

Collectively, our FRET studies to monitor LRR conformations concur with functional results obtained by electrophysiology and further emphasize the relation between LRRD mobility and activation.

### Structural properties of the mutants LRRC8-L402W and LRRC8-I2C-L402W

**Fig. 4:**
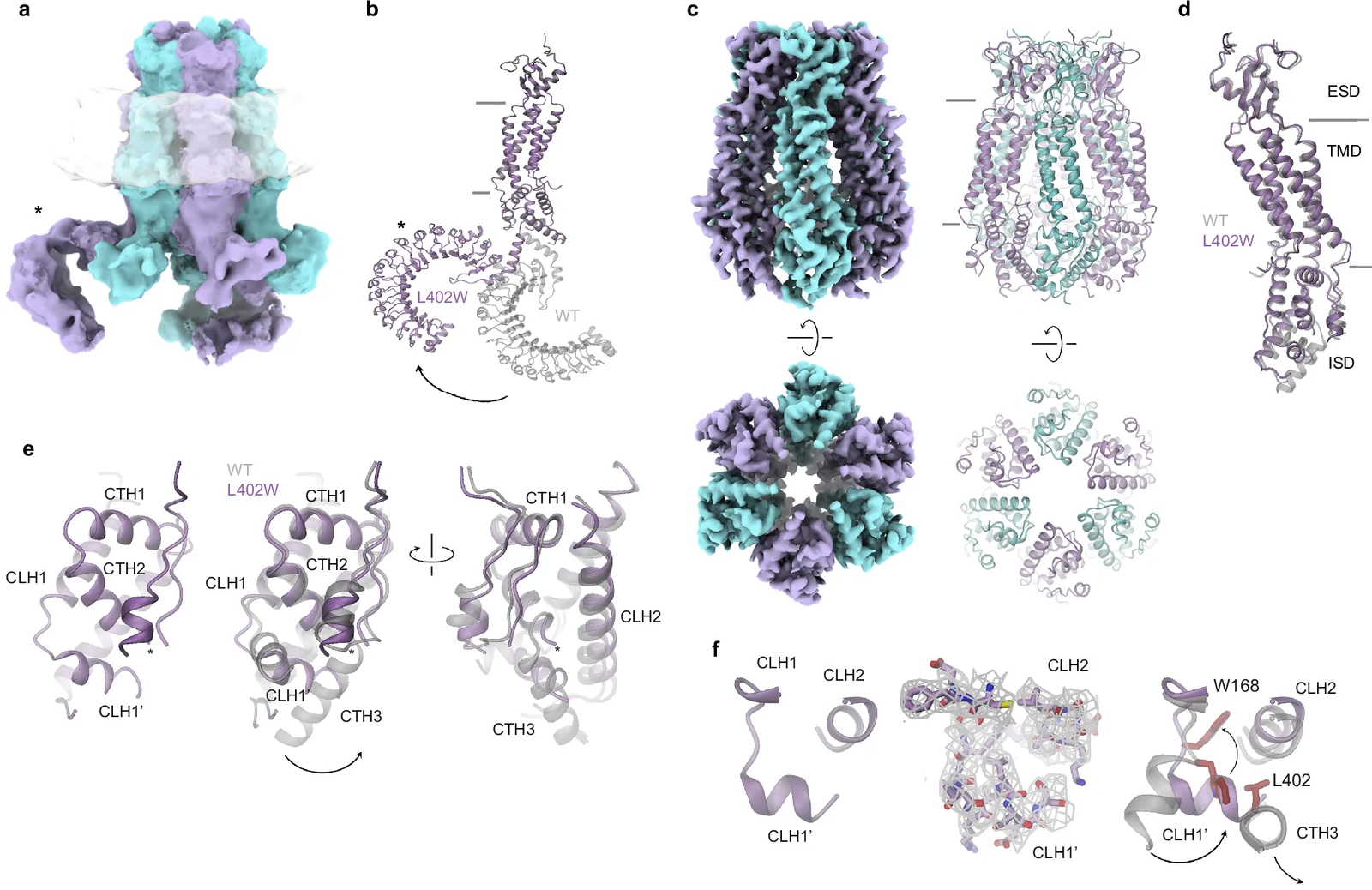
Cryo-EM structure of the activating LRRC8A mutant L402W. **a** A Low-resolution reconstruction of a selected 3D class of the L402W dataset in ATP showing large deviations of the LRRDs. **b** Superposition of the PDs of LRRC8A WT and the indicated subunit (*) displays a strong outward movement by 63° of the LRRD in L402W. **c** Map at 2.78 Å (left) and ribbon representation of the refined model (right) of the L402W dataset in the absence of ATP in indicated orientations. **d** Superposition of the PD of a single subunit of WT and L402W. **b–d** Membrane boundaries are indicated. **e** Blowup of the ISDs from the superposition shown in d in indicated orientations. **f** Conformational change in the ISD of L402W shown as ribbon (left), as stick model with cryo-EM density superimposed as mesh (center) and as superposition with WT with conformational changes indicated by arrows.

After identifying L402W as an LRRC8A mutant with strongly enhanced activation properties and showing by FRET that the mutation is associated with an altered LRRD arrangement that resembles an activated state, we were interested in the detailed features underlying its functional behavior. To characterize its structural properties, we overexpressed the protein by transient transfection of LRRC8^-/-^ cells (carrying genetic knockouts of all five LRRC8 subunits)^48^ and purified a sample in the detergent GDN. We subsequently determined structures by cryo-EM from two samples in the presence and absence of ATP, which was previously found to facilitate activation, at 3.4 and 2.8 Å, respectively (Supplementary Fig. 5 and Table 1). In both datasets, we obtained equivalent representations of the channel, with a C6 symmetric arrangement of the pore domain (PD) and strong conformational variability of the LRRDs. The latter is apparent in the dataset from the sample containing ATP, where certain 2D classes show a pronounced rearrangement of LRRD orientations leading to an expansion of the width of the protein (Supplementary Fig. 5b). The widening of the intracellular region is also illustrated in a low-resolution envelope after ab initio 3D classification, which shows a strong movement of one LRRD away from the pore axis towards the membrane that was not observed in WT (Fig. 4a and Supplementary Fig. 5b). The movement can be approximated by an outward tilt as rigid body by 63° around a pivot located at the boundary between the PD and the LRRD at the start of CTH3 (Fig. 4b). Upon further map refinement, the density of the LRRDs averaged out as a consequence of their large heterogeneity in the sample (Supplementary Fig. 5b). Although nominally at 3.4 Å resolution, the quality of the density was compromised due to preferred particle orientation and was thus not suitable for a detailed structural interpretation (Supplementary Fig. 5c–e).

**Table 1:** Cryo-EM data collection, refinement and validation

	Dataset 1 LRRC8A-L402W with ATP (EMD-55483) (PDB 9T34)	Dataset 2 LRRC8A-L402W (EMD-55484) (PDB 9T35)	Dataset 3 LRRC8A-I2C-L402W (EMD-57359) (PDB 29TC)	Dataset 4 LRRC8A-W168L-L402W channel trimer (EMD-55481)	Dataset 5 LRRC8A-W168L-L402W (EMD-55482) (PDB 9T33)
**Data collection and processing**					
Magnification	130,000	130,000	130,000	130,000	130,000
Voltage (kV)	300	300	300	300	300
Electron exposure (e^–^/Å^2^)	62.98	60.61	64.03	56.04	56.04
Defocus range (μm)	−0.8 to −2.4	−0.8 to −2.4	−0.8 to −2.6	−0.8 to −2.6	−0.8 to −2.6
Pixel size (Å)	0.651	0.651	0.651	0.651	0.651
Symmetry imposed	C6	C6	C6	C3	C1
Initial particle images (no.)	37,304	126,060	1,912,386	391,902	538,894
Final particle images (no.)	7,657	42,551	186,012	334,498	257,619
Map resolution (Å) FSC threshold	3.38 0.143	2.78 0.143	2.54 0.143	4.18 0.143	2.65 0.143
Map resolution range (Å)	3.4–4.2	2.8–3.5	2.5–3.0	4.2–7.4	2.7–3.3
**Refinement**					
Initial model used (PDB code)	5ZSU	5ZSU	9T35		5ZSU
Model resolution (Å) FSC threshold	3.7 0.5	3.0 0.5	2.7 0.5		2.8 0.5
Model resolution range (Å)	3.2–3.7	2.7–3.0	2.4–2.7		2.5–2.8
Map sharpening *B* factor (Å^2^)	71.4	89.1	97.0		61.3
Model composition Non-hydrogen atoms Protein residues Ligands	15,342 1830 0	15,342 1830 0	15,342 1830 0		14,572 1732 0
*B* factors (Å^2^) Protein Ligand	79.70 –	81.11 –	102.81 –		72.18 –
R.m.s. deviations Bond lengths (Å) Bond angles (°)	0.002(0) 0.359(0)	0.002(0) 0.352(6)	0.003(0) 0.458(0)		0.002(0) 0.355(6)
Validation MolProbity score Clashscore Poor rotamers (%)	1.69 3.39 3.18	1.49 2.74 2.47	1.50 3.85 1.35		1.65 2.64 4.53
Ramachandran plot Favored (%) Allowed (%) Disallowed (%)	96.97 3.03 0.00	97.31 2.69 0.00	96.52 3.48 0.00		97.51 2.49 0.00

An equivalent view of the protein was obtained from the better resolved dataset in the absence of ATP, which defined the conformation of the PD at 2.8 Å following the application of C6 symmetry (Fig. 4c and Supplementary Fig. 5f–j). While, like in the refined map from the dataset in the presence of ATP, the density of the LRRDs is not resolved, the structure reveals the molecular details of the PD at high resolution, including the conformational changes in its ISD that likely carry features that underlie the strong activating phenotype of the mutation (Fig. 4d–f). This structure shows a very similar organization of the ESD and the TMD as observed in WT and a conformational rearrangement of the ISD that is well defined and that strongly affects the relationship between the PD and the LRRD (Fig. 4d–f). The described structural features are reflected in the RMSD of 2.6 Å of the entire PD, 0.8 Å of the same unit excluding the ISD, and 4.2 Å for the ISD, based on the superposition of all six chains of L402W and WT. The conformational changes in the ISD are a consequence of the mutation of Leu 402 located on terminal helix CTH3 at the interface with the α-helix CLH2 preceding TM3 (Fig. 1a). The replacement with the bulkier Trp side-chain results in a disturbance of the interaction with CLH2 leading to a 2 Å outward movement of the latter and a much larger movement of CTH3 and its connected LRRD, whose densities are both not defined in the refined map (Fig. 4e). The resulting gap opened by the movement of CTH3 is filled by a conformational change of the short helix CLH1’, which succeeds the helix CLH1 connected to TM2 (Fig. 4e, f). The resulting 90⁰ rotation along a hinge in the loop region connecting to CLH1 leads to the formation of firm contacts with CLH2 (Fig. 4f). The rearrangement in this region is also accompanied by a 2 Å displacement of the loop preceding CTH1 as response to tighten interactions after the movement of CTH3 (Fig. 4e). The structure of the LRRC8A mutant L402W has thus revealed the conformational rearrangements leading to the strong activation of the mutant (Fig. 1h, i) and it has confirmed the break of the tight domain interactions indicated in FRET measurements (Fig. 3c).

To clarify whether similar structural properties would also be observed in the construct used for FRET studies, we have determined the structure of the double mutant 8A-I2C-L402W that combines the strongly activating L402W with a mutation on the N-terminus exerting the opposite effect to demonstrate that the inhibitory mutation did not perturb the structural rearrangements of the former (Supplementary Fig. 6 and Table 1). In this dataset, we found equivalent strong distortions in the conformation of the LRRDs in low-resolution reconstructions as observed in the data of LRRC8A-L402W (Supplementary Figs. 5b and 6c). As in the case of the single mutant, the LRRD density vanishes in the high-resolution reconstruction at 2.5 Å (Supplementary Fig. 6f), whereas the ISD shows the same conformational rearrangement leading to the dissociation of the terminal helix CTH3, which is not defined in the density (Supplementary Fig. 7). Similarly, we did not find pronounced density for the N-terminus, which remains disordered. Instead, we found the appearance of a crosslink leading to the formation of dimers on SDS-PAGE gels under non-reducing conditions that was not observed in the single mutant (Supplementary Fig. 6b), which suggests that the introduction of a cysteine residue leads to the formation of inter-subunit disulfide bridges, which could block the pore at the region of the N-terminus. Consequently, this data confirms that the mutation of sites in the ISD, while essential in the transduction of a conformational change leading to pore opening, acts independently of the actual gate of the pore that regulates the pore conformation.

### Structural properties of the double mutant LRRC8A-W168L-L402W

**Fig. 5:**
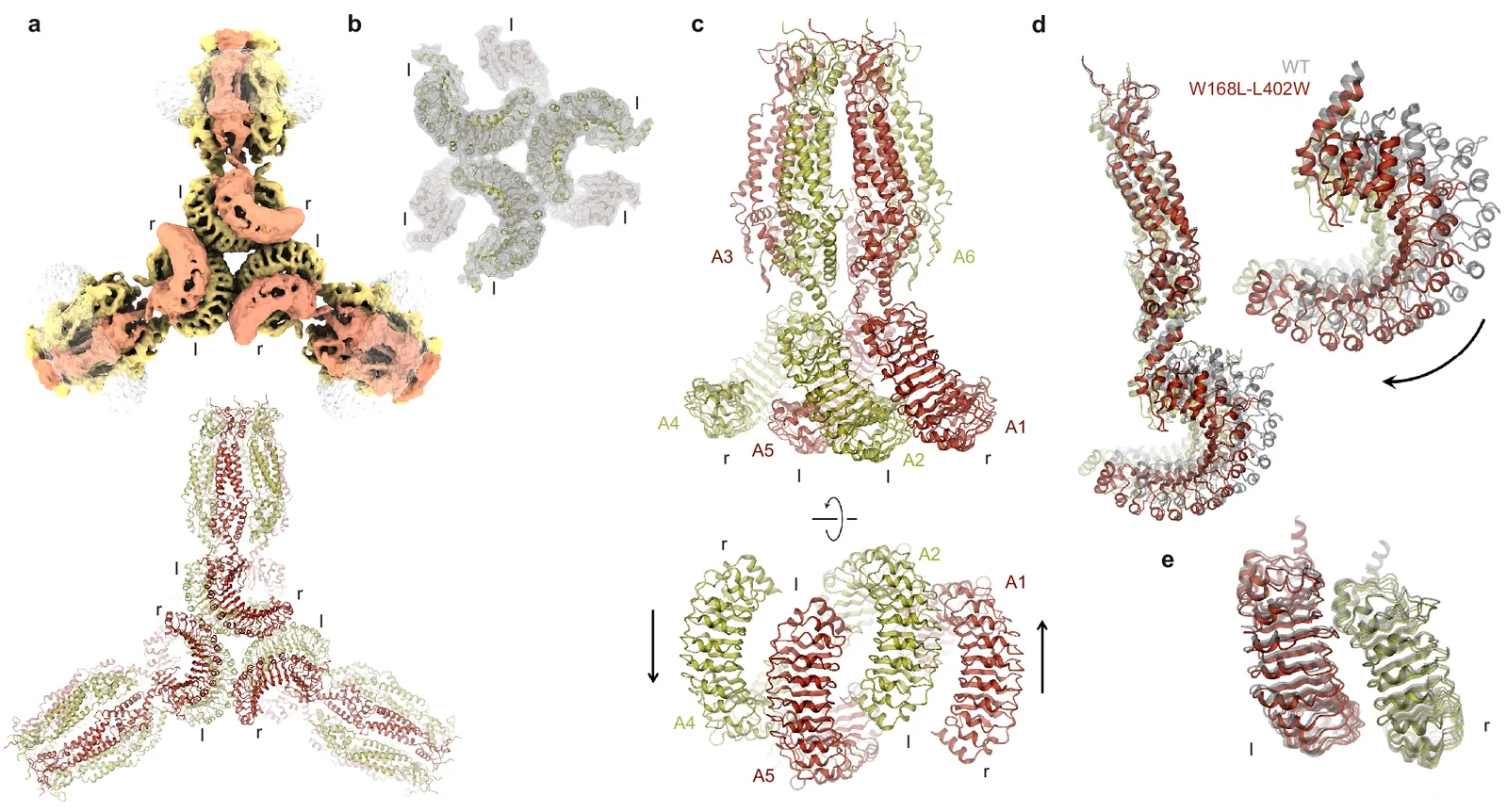
Cryo-EM structure of the LRRC8A double mutant W168L-L402W. **a** Map at 4.3 Å (top) and ribbon representation of the refined model (bottom) of a trimeric assembly of channels of the double mutant W168L-L402W. **b** Model of interacting LRRDs in the assembly with superimposed cryo-EM density. **c** Structure of the hexameric assembly of the W168L-L402W channel in indicated orientations. Shown is the relationship between domain pairs of subunits in A1, A2 and A4, A5 positions, with the direction of their movement relative to WT indicated. The LRRDs in A3 and A6 positions were not defined in the density and are thus not included in the model. **d** Superposition of the PD of pairs of tightly interacting subunits of WT and the double mutant W168L-L402W. Inset (right) shows a blow up of the LRRDs, arrow indicates the direction of movement from WT to the mutant. **e** The superposition of tightly interacting LRRD pairs of WT and W168L-L402W shows an undisturbed relationship between interacting LRRDs in the mutant. **a–d** l and r indicate left and right positions in tightly interacting LRRD pairs.

In the structure of LRRC8A, the sidechain of Leu 402 is in van der Waals (VDW) distance to the bulky Trp 168 on CLH1’ (Fig. 2a). We thus reasoned that the mutation of the latter could compensate for the increased sidechain volume of the mutant L402W, causing the partial reversion of the functional phenotype of the double mutant W168L-L402W towards WT that was found in the data from electrophysiology and FRET microscopy (Figs. 2c, d and 3d). We thus decided to determine the structure of this double mutant to better understand the basis for its intermediate phenotype. To this end, we prepared the protein analogously to the single mutant L402W in the absence of ATP and recorded data by cryo-EM (Supplementary Fig. 8 and Table 1). In this dataset we found particles of two distinct sizes with one population (encompassing 38% of picked particles) resembling WT and the mutant L402W, and a second population (encompassing 62% of picked particles) of much larger size (Supplementary Fig. 8b). After 2D classification, 3D reconstruction and map refinement, the particle population of increased size turned out to originate from trimeric assemblies of LRRC8A-W168L-L402W hexamers that interact via their C-terminal parts (Fig. 5a, b, Supplementary Figs. 8b and 9a). This interaction is a consequence of a rearrangement of the cytoplasmic LRRDs, where pairs of well-defined and tightly interacting domains alternate with a single unpaired subunit, where the domain is mobile and thus not defined in the density (Fig. 5a, c and Supplementary Fig. 9a, b). This alternating arrangement has allowed the two LRRDs on the left side to slide towards each other to maximize contacts between domain pairs oriented in opposite directions, while largely preserving the mutual relationship between l and r subunits (Fig. 5c–e). The resulting formation of a C2-symmetric arrangement has created a flat platform that facilitated the trimerization of hexamers, which in turn has further stabilized the observed domain organization (Fig. 5b). While this unique domain arrangement was stabilized in the trimeric channel assembly, we assume that it would also exist in isolated hexamers. However, in classes of such isolated hexamers, the domain conformation is more dynamic and less well defined in the density and thus difficult to assign with confidence (Supplementary Figs. 8b and 9c). Despite the ambiguity in the LRRD conformation, the equivalence of the structure of the PD in both particle populations is evident when comparing its density obtained from isolated particles (at 2.95 Å) with the density obtained upon masking one of the three channels, which was improved to 2.65 Å, where the density of the LRRDs has vanished in both cases (Supplementary Figs. 8f–g and 9d). This resulting map defined the structure of the double mutant, where the PD essentially displays the same features as WT (with RMSDs of 1.7 Å for the entire PD, 0.71 Å after exclusion of the ISDs, and 2.0 Å of the ISDs, upon superposition of models containing all subunits of the hexamer). As expected from the single mutant L402W, the structural deviations compared to WT in the ESD and TMD are minimal, whereas they are enlarged in the ISD carrying the two mutations. In this region, we did not find density for helix CTH3 carrying the mutation L402W, and CLH1, the site of the mutation W168L, whose rearranged conformation in the single mutant L402W is stabilized by Trp 168 (Fig. 4f and Supplementary Fig. 9e, f).

In summary, the structure of the double mutant 8A-W168L-L402W revealed structural features that differ from both WT and L402W, which may explain its intermediate functional phenotype. Different from L402W, the destabilization of the LRRD conformation is less severe and instead results in a domain arrangement that is presumably facilitated by a repacking of residues at the site of the mutations, which in turn affects the conformational mobility of the LRRDs moving as rigid units. Together with functional results, it further supports the notion of the ISDs acting as regulators of the open probability of the channel by mediating conformational changes between the LRRDs and a still unknown channel gate.

## Discussion

**Fig. 6:**
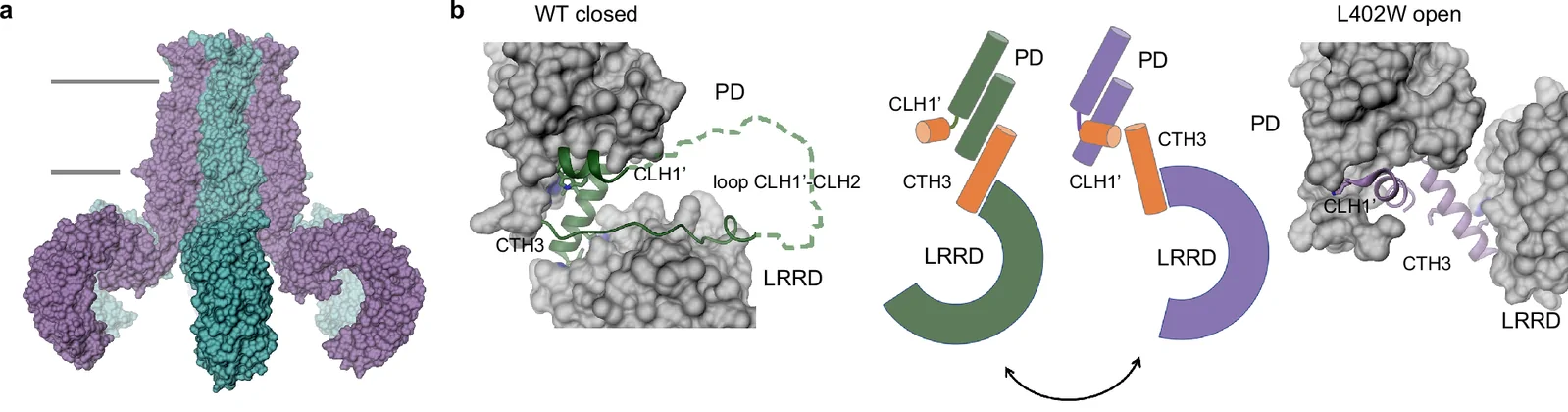
Schematic properties of mutants leading to channel activation. **a** Hypothetic model of an activated channel that is based on a hexameric assembly of the subunits of the mutant L402W with strong outward movement of the LRRD shown in Fig. 4b. The model is represented by its molecular surface. **b** Scheme of the conformational changes in the ISD and the LRRD of a single subunit between WT (left) and the mutant L402W (right). Shown are surfaces of the LRRD and the ISD except for the moving elements CLH1’ and CTH3, which are displayed as ribbons. Cartoons (center) show schematic movements of the LRRD by the dissociation of CTH3 from its interaction interface in the ISD.

Here, we have addressed the role of the ISD of LRRC8A as mediator of conformational changes of the LRRDs leading to channel activation. By bridging the cytoplasmic domain with the transmembrane pore, this unit occupies a strategic location to transmit signals received by the LRRDs to release a gate in the PD, whose location is still debated. The results of the study are in full agreement with previous work that has identified two variants in the ISD of the LRRC8C subunit, causing the same severe multi-system disorder^46^. These include a conservative point mutation replacing a Val with a Leu and a nonsense mutation that results in a premature stop of translation at the boundary to the LRRD. Both variants confer a dominant gain-of-function that leads to constitutive channel activity even under resting conditions^46^. By studying its functional properties by electrophysiology, we were able to confirm here a similar strong gain-of-function phenotype for the equivalent point mutation (V392L) in LRRC8A. This mutant is located on one of the helices (CTH2) that bridges the PD to the LRRD and strongly increases the activation properties of channels consisting of A and E subunits (Fig. 1d, f). A considerably more severe functional phenotype was found for the mutation of a position located 10 residues further downstream in CTH3, the terminal helix of the pore domain. At the equivalent location in one of the disease-causing variants of LRRC8C, a single nucleotide insertion at residue 400 has altered this position from a Leu to an Ile, followed by a premature termination of the protein eight residues later. In LRRC8A, mutation of the corresponding Leu 402 to Trp resulted in strong basal activity for both LRRC8A/E channels and homomeric LRRC8A (Fig. 1g–i). The mutation thus reverts the properties of the latter, which, when expressed on its own, resides in a stable closed conformation with very low open probability even under hypotonic conditions^29,43^. The fact that other mutations, including the truncation of the side-chain to an alanine and even a conservative replacement by an Ile, showed a similar effect (Fig. 1j–n), defines the shape of the sidechain as a critical property for maintaining WT properties. The described mutations have perturbed the local structure of the ISD and its ensuing LRRD as shown by cryo-EM and cellular studies (Figs. 3–5). To monitor conformational changes of the LRRD during activation and their altered distribution in the mutants, we relied on FRET studies employing suitably labeled subunits containing fusions to fluorescent proteins^42,49^. These studies show a clear decrease of FRET in homomeric LRRC8A channels upon exposure to a hypotonic environment as a consequence of the disruption of the tight arrangement found under isotonic conditions (Fig. 3a). Corresponding changes were also obtained upon expression of nanobody-based binders that either inhibit the channel leading to a FRET increase or activate the channel, thereby lowering FRET (Fig. 3e). In agreement with its constitutive activity observed in electrophysiology, we found FRET in the mutant L402W to be consistently low as in the activated state of WT, suggesting a domain rearrangement leading to the separation of FRET pairs (Fig. 3c). This was confirmed in the cryo-EM structure of the mutant L402W, where the LRRDs were found as highly mobile and where a large, about 60° movement of the domain away from the symmetry axis was observed in a low-resolution reconstruction (Fig. 4a–c). The same strong distortion of the LRRD was also observed in a double mutant I2C-L402W, which combines the activating mutation L402W with another mutation at the N-terminus that was previously shown to prevent channel opening^47^ (Supplementary Figs. 6 and 7), further emphasizing the role of the ISD as mediator in the coupling to a distinct gate region that regulates the pore conformation. The observed movement of the LRRD would bring this unit into the vicinity of the lipid bilayer and could make a direct membrane interaction a plausible scenario (Fig. 6a). Remarkably, a similar distortion is also found in a study describing the properties of heteromeric LRRC8A/D channels in the presence of the sybody Sb4, which acts as a potentiator to increase channel activity^50^.

The detailed impact of the mutation in the ISD on channel conformations was defined in reconstructions of 8A-L402W and 8A-I2C-L402W at high resolution, where in both cases the LRRD density has vanished due to their increased structural heterogeneity (Fig. 4e, f and Supplementary Fig. 5, 6). In these maps, the structured part of the ISD is well-defined, apart from the terminal helix CTH3, which has dissociated from its tight interaction with helix CLH2, as a result of the perturbation of the interaction site. As a consequence, the short helix CLH1’, which is part of an extended loop region that is connected to its preceding helix CLH1 via a mobile linker, has changed its conformation to fill the hydrophobic gap opened by the dissociation of CTH3, thereby locking the interaction site in a conformation that could be critical for channel activation (Fig. 4f and Supplementary Fig. 7b). CLH1’ could thus act like a doorstop that stabilizes the open conformation by hampering the reassociation of CTH3 with the ISD. The flexible loop region connecting CLH1 and CLH2 is remarkable since it contains, besides the short helix CLH1’, several putative phosphorylation sites. This region regulates the trafficking of LRRC8A and its insertion into other subunits permits their shuttling to the plasma membrane, turning these chimeric constructs into activatable channels^37^. In the high-resolution structure of the LRRC8A/Sb1 complex, the 40 residues following CTH1’ are mobile and thus not defined in the structure, which allows the observed helix rearrangement. In contrast, the following 17 residues at the C-terminus of the loop bind to the surface of the LRRD in subunits located in the r-position, thereby potentially stabilizing the domain conformation^29^. Our structures thus emphasize the role of CTH3 as a suitable coupling element between the LRRD and the remainder of the ISD by forming tight interactions with both parts of the protein (Fig. 6b).

Our study has also identified the residue Trp 168, located at the end of CLH1’, to engage in VDW contacts with L402, and we thus investigated whether its replacement in the double mutant W168L-L402W would compensate for the increase in the side-chain volume of L402W and thus revert the effect of the single mutant. While our electrophysiology and FRET data of  W168L-L402W reveal properties that are intermediate between WT and the single mutant L402W, our structures have illustrated that this phenotype is due to a conformation where, as a consequence of both side-chain replacements, the domains have rearranged while keeping their pairwise contacts that are a hallmark of the LRRDs of the 8A subunit (Fig. 5c).

In summary, our study has defined the ISD as a region involved in the transmission of conformational changes from the LRRDs to the PD to open a gate that allows ion conduction. The assigned role of the LRRDs acting as allosteric modules for activation supports previous observations, where channel activation was accompanied by an increase in domain mobility^42,43,46^. In a resting state, the tight association between LRRDs of the 8A subunits stabilize a closed channel conformation. This stable conformation can either be perturbed by binders^43^, mutations^46^ or the formation of heteromeric channels that destabilize the LRRD domain arrangement by introducing subunits with altered interaction properties of their LRRDs^34,35,45^. In all cases, we found the structures of the extracellular and membrane-inserted parts of the PD not to be visibly affected, which renders the ensuing step in the activation from the ISDs still elusive. Despite these open questions, the described results have laid the foundation for detailed follow-up studies on the coupling between the ISDs and a channel gate.

## Methods

### Oocyte preparation and molecular biology

Oocytes from *Xenopus laevis* were purchased from Ecocyte Biosciences (Dortmund, Germany). Human LRRC8A and LRRC8E subunits and the variants used in this study were subcloned into the pCSDest vector^41^. For oocyte expression, the LRRC8E subunit was C-terminally fused to mCherry (mCh). Single point mutations were introduced by site-directed mutagenesis PCR using primers listed in Supplementary Table 1. cRNA was transcribed with the SP6 mMessageMachine kit (Thermo Fisher Scientific) following linearization with *Mlu*I (Thermo Fisher Scientific). Oocytes were injected with ~6 ng RNA per subunit and incubated at 18 °C for 1–3 days prior to electrophysiological recordings.

### Fluorescence microscopy to quantify cell surface expression

Oocytes were injected with 5 ng of RNA encoding 8A-VFP or 8A-L402W-VFP. After 2 days of expression, surface fluorescence signal was measured using a spinning-disk confocal microscope (Evident, Milan) equipped with a 60x oil immersion objective. To this end, oocytes were oriented with the dark side facing the objective to reduce autofluorescence and fluorescence from cytosolic proteins. The same laser power and exposure time were used for all recordings. Average fluorescence was calculated over a region of interest in focus using Fiji^51^.

### Two-electrode voltage clamp

Recordings were performed using the GePulse acquisition software (http://users.ge.ibf.cnr.it/pusch/programs-mik.htm). The holding potential was −30 mV, and current–voltage relationships were obtained with 3 s voltage steps from −100 to +60 mV in 40 mV increments. To estimate LRRC8-mediated currents at different potentials, the IV-pulse protocol was applied: a prepulse to −100 mV for 200 ms was followed by recordings at distinct voltages ranging from −100 to 60 mV in 20 mV increments for 3 s.

For assessment of channel expression and osmotic sensitivity, oocytes were maintained in an isotonic bath (215 mOsm) containing 100 mM NaCl, 5 mM MgSO₄, 10 mM HEPES, pH 7.3. The hypertonic solution was prepared by adding 100 mM mannitol. Hypotonic stimulation was applied by diluting the isotonic solution 1:1 with H₂O (120 mOsm). Carbenoxelone (Sigma) was directly dissolved in the isotonic solution at 100 µM. To monitor the activation kinetics during hypotonic stimulation, oocytes were clamped at −20 mV, interrupted by 200 ms pulses to +60 mV in 5 s intervals, with averaged currents over the pulse period plotted as a function of time.

Ion selectivity was determined by substituting NaCl with sodium glutamate, NaI or NMDG-Cl or by using a NaCl-free solution composed of 10 mM HEPES, pH 7.3 and 5 mM MgSO_4_.

### Determination of relative glutamate selectivity

The shift in reversal potential (ΔE_rev_), measured in 100 mM chloride and 100 mM glutamate solutions, was determined for oocytes co-injected with 8A- or 8A-L402W-VFP and 8E-mCh. Since current amplitudes for 8A-VFP/8E-mCh co-injected oocytes were smaller than those in 8A-L402W injected oocytes, and thus more affected by leak/background currents, for these experiments, currents measured in 200 µM DCPIB were subtracted for both conditions (100 mM chloride and 100 mM glutamate). Salt-bridges were used as reference electrodes, and a liquid junction potential of 8 mV was determined as described^52^ and added to the measured shift in reversal potential. The relative permeability, P_Glut_/P_Cl_, was calculated as

1 $$\frac{{P}_{{Glut}}}{{P}_{{Cl}}}=\exp \left(\frac{-F{\Delta E}_{{rev}}}{{RT}}\right)$$

where F is Faraday’s constant, R the gas constant and T the temperature.

### Statistical analysis of electrophysiological data

Electrophysiological data were analysed using paired measurements obtained from the same oocyte before and after hypotonic stimulation. Current amplitudes were quantified from 50 ms pulses to +60 mV during the time-course of recordings. Solution exchange was complete after 20 s of perfusion. Thus, for summary plots, current values measured 20 s after switching to hypotonic solution (*t* = 0) were compared with values obtained after ~300 s of hypotonic perfusion, corresponding to an exposure of ~280 s.

Statistical significance was assessed using a two-tailed paired ratio t-test, which evaluates whether the geometric mean of the ratio between paired measurements differs significantly from unity. Individual oocytes were treated as independent experimental units. Data are presented as mean ± s.e.m., with individual measurements shown as dots in the figures. Sample sizes (n) correspond to the number of recorded oocytes and are indicated in the respective figure legends.

### Expression constructs for Förster Resonance Energy Transfer (FRET) measurements

For FRET experiments, hLRRC8A (8A) and the mutated sequences for 8A-W168L, 8A-L402W and 8A-W168L-L402W were subcloned into pECFP-N1-LRRC8A-mCerulean3 and pECFP-N1-LRRC8A-mVenus^42^ via *Eco*RI and *Sbf*I restriction sites to generate donor (mCerulean) and acceptor (mVenus) pairs. The VRAC ion conductance diminishing mutations I2C and E6A were introduced in these expression constructs by site-directed mutagenesis (Supplementary Table 1). All constructs were confirmed by Sanger sequencing (Microsynth AG) of the open reading frame.

### Stimulated Emission Förster Resonance Energy Transfer (SE-FRET)

SE-FRET measurements were performed as previously described^49^. Briefly, one day before transfection, 5 × 10^4^ HeLa cells (Leibniz Forschungsinstitut DSMZ) were seeded onto glass-bottom dishes in DMEM supplemented with 10% fetal bovine serum (FBS) and 1% penicillin-streptomycin (Pen/Strep; 50 U ml^−1^/50 µg ml^−1^) at 37 °C and 5% CO_2_. Cells were either transfected with the donor or acceptor constructs individually to determine FRET correction factors or in combination (donor and acceptor) for SE-FRET measurements. Cells were transfected with 500 ng (single transfection) or 1000 ng (double transfection) of pDNA with PEI at a 1:3 ratio, prepared in Opti-MEM. One day after transfection, SE-FRET experiments were performed using a Leica THUNDER Imager (Leica) equipped with a Leica LED8 lamp, the filter cube CYR71010, an HC PL APO 63x/1.40 OIL objective, long pass filter for 460/80 and 553/70 and a Leica DFC9000GTC camera using the SE-FRET plugin for the Leica LAS X software. Before image acquisition, cells were bathed in isotonic solution (150 mM NaCl, 6 mM KCl, 1.5 mM CaCl_2_, 1 mM MgCl_2_, 10 mM D(+)-Glucose and 10 mM HEPES; 340 mOsm). Cells were imaged for 9 cycles per condition with different bath solutions applied in the following sequence with an interval of 10 s: baseline (isotonic solution), hypotonic solution (same composition as isotonic solution except for a decreased concentration of NaCl to 105 mM; 240 mOsm), isotonic solution, hypertonic solution (isotonic solution supplemented with 160 mM D(-)-Mannitol; 500 mOsm) and isotonic solution. Raw SE-FRET values of each region of interest (ROI, representing one cell) were normalized against the mean SE-FRET value of the baseline of the corresponding region. The mean of all ROIs in one field of view (FOV) was used for data representation and statistical analysis.

### FRET acceptor bleaching

For the acceptor bleaching experiments, HeLa cells were seeded, co-transfected and imaged with the microscopy set-up described for SE-FRET. Cells used for acceptor bleaching under different tonicities were co-transfected with 500 ng of each FRET construct (donor and acceptor). Cells used for acceptor bleaching with synthetic nanobodies (sybodies) under isotonic conditions were co-transfected with 250 ng of each FRET construct together with 1500 ng of constructs encoding Sb1 or Sb4 (ref. ^43^). Before image acquisition, cells were bathed in an isotonic solution. For the image acquisition, cells were positioned in the FOV so that only a part of the cells remained illuminated by adjusting the field diaphragm (Supplementary Fig. 10a). An image of the donor (excitation at 440 nm and emission at 460/80 nm) and acceptor (excitation at 510 nm and emission at 535/70 nm) was taken (pre-bleaching). The field aperture was closed, and the acceptor was bleached with 100% LED intensity at 510 nm for 90 s, followed by imaging of the donor and acceptor fluorescence with an open aperture (post-bleaching) (Supplementary Fig. 10b). After repeating for different FOVs on the same dish, the bath solution was exchanged to a buffer with different tonicity, and the process was repeated with different FOVs. Bath solutions: isotonic buffer (340 mOsm), 300 mOsm (same as isotonic solution except for 130 mM NaCl), 250 mOsm (105 mM NaCl) and 200 mOsm (80 mM NaCl).

For the quantification of FRET, the images prior to bleaching were segmented using Cellpose v3.1 (ref ^53^) to generate a labeled image containing masks for each cell in the FOV (Supplementary Fig. 10c). A mask for the bleached area was generated from an image of the acceptor channel without sample acquired with an adjusted diaphragm. The part of each cell mask within the bleached area was used to measure the mean fluorescence intensity of the donor and acceptor channels pre- and post-bleaching. Cells located entirely within the bleach area were excluded from the analysis. FRET efficiency was calculated according to following formula and negative FRET efficiencies were excluded.

2 $$\%{{\mathrm{FRET}}}=\frac{\left({{\mathrm{donor}}}\,{{\mathrm{post}}}\,{{\mathrm{bleaching}}}\right)-({{\mathrm{donor}}}\,{{\mathrm{pre}}}\,{{\mathrm{bleaching}}})}{{{\mathrm{donor}}}\,{{\mathrm{post}}}\,{{\mathrm{bleaching}}}}\,x\,100$$

### Immunofluorescence imaging of cell surface expression

LRRC8A, containing an HA tag in the extracellular loop between TM3 and 4 (ref. ^4^), was subcloned into pEGFP-C1 via *EcoR*I and *Kpn*I restriction sites. The mutations W168L, L402W and W168L-L402W were introduced by site-directed mutagenesis (Supplementary Table 1). A control construct with a cytoplasmic HA tag at the C-terminus was generated by amplifying the cDNA of hLRRC8A with a primer pair introducing an HA tag sequence fused to the C-terminus (Supplementary Table 1). The PCR product was subcloned into pEGFP-C1 using *EcoR*I and *Kpn*I restriction sites.

For plasma membrane staining, 1 × 10^4^ HeLa cells were seeded on 16-mm glass coverslips in 12-well plates pretreated with poly-L-lysine and transfected with 1 µg plasmid DNA per construct using PEI as described above. 24 h after transfection, cells were washed with Dulbecco’s Phosphate Buffered Saline (DPBS), fixed with 4% paraformaldehyde (PFA) for 20 min, and excess PFA was quenched with 50 mM ammonium chloride in DPBS, followed by washing with DPBS. Samples were then incubated at room temperature for 1 h in either 3% BSA in DPBS supplemented with 0.1% saponin (permeabilized) or 3% BSA in DPBS without saponin (non-permeabilized). Cells were subsequently incubated overnight at 4 °C with a mouse anti-HA antibody (clone HA-11; Covance) diluted 1:100 in the respective permeabilization/blocking solution. After washing with DPBS, cells were incubated with an anti-mouse secondary antibody conjugated to Alexa Fluor 647 (abcam, #ab150111) diluted 1:500 for 2 h at room temperature, followed by washing with DPBS. Coverslips were mounted on glass slides with mounting medium (Thermo Fisher Scientific, #P36961).

GFP and Alexa Fluor 647 images were acquired using a Leica THUNDER Imager (Leica) equipped with a Leica LED8 lamp and a DFT51010 filter cube. Image display settings were adjusted for the weaker HA signal in non-permeabilized cells; consequently, signals in permeabilized cells appear saturated.

### Cell surface biotinylation

To assess cell surface expression by biotinylation, hLRRC8A (WT or the W168L, L402W, or W168L-L402W mutants) was cloned into pcDNA3.

For cell surface biotinylation experiments, HeLa LRRC8A knockout cells^14^, kindly provided by Z. Qiu, were seeded on 100 mm dishes and transfected the following day with 10 µg of expression construct. 24 h after transfection, cells were placed on ice, and all subsequent steps were performed on ice or at 4 °C with pre-cooled buffers. Cells were washed with DPBS and incubated with 0.5 mg/ml Sulfo-NHS-Biotin (Thermo Fisher Scientific, #21217) in DBPS for 30 min under agitation. Excess biotin was quenched by washing twice with 100 mM glycine in DPBS, followed by incubation in 100 mM glycine in DPBS for 20 min under agitation.

After washing twice with DPBS, cells were lysed in 600 µl lysis buffer (150 mM NaCl, 50 mM Tris/HCl, pH 8, 5 mM EDTA, 1% Triton X-100, 0.5% sodium deoxycholate, 0.1% sodium dodecyl sulfate) supplemented with protease inhibitor cocktail (Roche, #11836145001) for 30 min. Lysates were centrifuged at 16,000 *g* for 15 min, and 80% of the lysate was incubated overnight at 4 °C with rotation with 100 µl streptavidin-conjugated agarose beads (Thermo Fisher Scientific, #20349) equilibrated in lysis buffer. The remaining 20% was retained as input. Beads were washed three times by centrifugation and resuspension in lysis buffer with rotation for 5 min. Beads and input samples were then mixed with 120 µl and 40 µl, respectively, of 4x LDS buffer (Invitrogen, #NP0008) supplemented with 400 mM DTT and heated to 70 °C for 10 min. After sequential centrifugation at 1000 g and 20,000 g for 1 min, supernatants from the beads (30% of total membrane fraction) and input (5% of total input) were analyzed by SDS-PAGE and Western blotting.

After blocking with 5% milk in Tris-buffered saline (TBS) supplemented with 0.05% Tween 20 (TBS-T), the membranes were probed with antibodies against LRRC8A^4^ (kindly provided by T. J. Jentsch), GM130 (BD Biosciences no. #610823), Na/K-ATPase (Millipore, #05-369) and ß-actin (abcam, #ab8226). Protein bands were visualized by enhanced chemiluminescence (Thermo Fisher Scientific, #34075) after incubation with HRP-conjugated secondary antibodies (abcam, #6721 and #6789) diluted in 5% milk in TBS-T. Signals were detected using an Azure Biosystems 500Q, and band intensities were quantified using Fiji from non-saturated bands. Cell surface expression was calculated as the ratio of membrane fraction to input (total protein).

### Protein purification

For Cryo-EM sample preparation, constructs of full-length human LRRC8A were generated by FX-cloning^54^. Initially, expression constructs were cloned into the pInit vector. The single-site mutation of hLRRC8A-L402W and the double-site mutation of hLRRC8A-W168L-L402W (Supplementary Table 1) were modified in the pInit vector using a modified QuickChange protocol^55^ and subcloned into the pcDX vector as constructs containing the modified LRRC8A with a C-terminal Rhinovirus 3 C protease, mCherry, a myc-tag, and a streptavidin-binding peptide (pcDX-hLRRC8A-C3ChMS). All constructs were sequenced (Microsynth AG).

Protein expression was carried out in the HEK293 cell-line LRRC8^−/−^ containing genetic knockouts of all five LRRC8 family members, kindly provided by T. J. Jentsch. This cell line was adapted to suspension culture and growth in HyCell TransFx-H^TM^ medium (Cytiva) supplemented with 4 mM L-glutamine, 1% FBS, 100 U/ml Pen/Strep, and 1.5 g/l Poloxamer 188 at 37 °C and 5% CO_2_.

The day before transient transfection, cells were split to a density of 0.8 × 10^6^ cells/ml. The endotoxin-free plasmid (1.2 μg per 10^6^ cells) and PEI MAX 40000 were diluted and mixed at a 1:2.5 (wt/wt) ratio in DMEM medium. After transfection, cells were supplemented with 4 mM Valproic acid and incubated for 96 h in the case of the mutant 8A-L402W and for 72 h in the case of the double mutant 8A-W168L-L402W at 37 °C and 5% CO_2_. Cells were harvested by centrifugation at 500 g for 15 min, washed with PBS (at 4 °C), and pellets were flash-frozen in liquid N_2_ and stored at −80 °C.

For protein purification, all steps were carried out at 4 °C. The cell pellets were resuspended in 200 ml of buffer containing 25 mM Tris-HCl, pH 7.4, 50 mM NaCl, 50 μg/ml DNase, 10 μM leupeptin, 1 μM pepstatin, and 1 μM benzamidine. After 10 min, the suspension was supplemented with 2% of the detergent glyco-diosgenin (GDN, Anatrace) and incubated for 1.5 h for protein extraction. The sample was subjected to centrifugation at 8000 g for 50 min. The supernatant was incubated in batch with 4 ml of Streptactin superflow resin (IBA LifeSciences) for 1 h to bind solubilized LRRC8A constructs, transferred to a gravity column and washed with 20 CV of wash buffer (25 mM Tris-HCl, pH 7.4, 250 mM NaCl, 0.01% GDN). The bound protein was eluted with wash buffer containing 10 mM D-desthiobiotin (Sigma-Aldrich). The tag of the protein was cleaved by incubation with HRV 3 C protease (at a 1:250 molar ratio) for 1 h, and then concentrated to 500 μl using an Amicon Ultra centrifugal filter with a cut-off of 100 kDa (Merck). Finally, the protein was subjected to a Superose 6 10/300 GL (Cytiva) size exclusion chromatography column and eluted with SEC buffer (25 mM Tris-HCl, pH 7.4, 250 mM NaCl, 0.005% GDN). The purified protein was concentrated and used for the preparation of cryo-EM grids. For the mutant 8A-L402W prepared in the presence of ATP, the wash buffer and SEC buffer were supplemented with 2 mM of ATP.

### Cryo-EM sample preparation and data collection

For preparation of cryo-EM samples, cryo-EM grids (Quantifoil R1.2/1.3 Au 300 mesh) were glow-discharged for 60 s before vitrification. 2.5 μl of protein samples at a concentration of 2.5–3.5 mg ml^−1^ were applied on glow-discharged grids, incubated for 3 s, blotted for 5 s and flash-frozen in a mixture of liquid ethane/propane using a Vitrobot Mark IV system (Thermo Fisher Scientific). Grids were stored in liquid nitrogen until used for data collection.

Cryo-EM grids were imaged on a 300 kV Titan Krios G3i cryo-electron microscope (Thermo Fisher Scientific) with a 100 μm objective aperture and a post-column BioQuantum energy filter (Gatan) with a 20-eV slit. All data were acquired using a K3 direct electron detector (Gatan) in counted super-resolution mode at a magnification of 130,000 x corresponding to a pixel size of 0.651 Å/pixel (0.3255 Å/pixel in super-resolution) with a total exposure time of 1.25 s (consisting of 47 individual frames). All micrographs were collected with a defocus range from −0.8 to −2.4 μm using EPU 3.9 and AFIS faster acquisition (Thermo Fisher Scientific). The total electron dose on the specimen was 63 e^−^/Å^2^ for two datasets of the single-mutation 8A-L402W and 60 e^−^/Å^2^ for the dataset of the double-mutant 8A-W168L-L402W. The structures of L402W in the presence or absence of ATP, of I2C-L402W and of 8A-W168L-L402W were determined from datasets containing 16,279, 25,496, 32,257 and 27,985 micrographs, respectively.

### Cryo-EM image processing

All datasets were processed in cryoSPARC v4.7.1 (ref. ^56^). After motion correction and CTF estimation, the particles were picked using blob picker with a maximum particle diameter of 180 Å and selected to obtain an improved template. For the datasets of L402W in the presence of ATP, additional particles were picked with a trained Topaz model^57^, 2D classified to generate an ab initio model and separated into two different volumes after 3D classification. One of the maps with a large LRRD movement of one subunit, containing 7657 particles, was processed by non-uniform refinement with C1 symmetry to a resolution of 3.9 Å. Subsequently, the map quality was improved to 3.8 Å after local CTF refinement and non-uniform refinement with C6 symmetry. Particles of 8A-L402W without ATP were picked and selected in several topaz training sessions and 2D classification. The initial map was generated using non-uniform refinement with C1 symmetry after ab-initio reconstruction and heterogeneous refinement with a resolution of 3.14 Å. To investigate the mobility of the LRR domains and improve the map quality, the map was processed as four different volumes after 3D classification, heterogeneous refinement, and non-uniform refinement, maintaining C1 symmetry. Three of four maps were of very low resolution, ranging between 12.01 Å and 9.7 Å, and one of four maps was at a substantially higher resolution of 3.10 Å, which was further improved to 2.78 Å after local CTF refinement and non-uniform refinement applying C6 symmetry.

For the dataset of the double-mutant I2C-L402W, micrographs were processed using motion correction (at a pixel size of 0.651 Å) and CTF estimation. An initial set of particles was extracted with a box size of 500 pixels. Particles were picked using a template picker and divided into two groups: the first group consisted of particles with clear LRR domain features, which were classified to generate three ab initio models representing different LRR conformations. The remaining particles, which primarily displayed the pore domain, were used to generate a model that was refined via heterogeneous refinement and Non-Uniform Refinement with C1 symmetry to a resolution of 2.4 Å. However, this map was anisotropic due to the strongly preferred particle orientation in this reconstruction. The particles were thus further classified, leading to an improved map showing less anisotropy in a final stack of 186,012 particles extending to 2.6 Å. This map was further improved to 2.54 Å after 3D classification and NU refinement with C6 symmetry applied.

For the dataset of double mutant W168L-L402W, the micrographs were processed using motion correction (at a binned pixel size of 1.302 Å/pixel) and CTF estimation, and an initial set of particles was extracted with a box size of 300 pixels. After 2D classification, two different assemblies of particles were obtained. One set of particles is of similar size as WT and contains a single hexameric channel, the second set of considerably larger particles contains an assembly of three interacting hexamers. Two initial maps of the larger assembly were generated and separated in an ab initio reconstruction and two rounds of heterogeneous refinement to remove junk particles. This map was further refined to a higher resolution of 2.65 Å after non-uniform refinement with C1 symmetry, containing one single hexameric channel, which lacks the density of LRRDs. For the reconstruction of the entire trimeric channel complex, the particles were re-extracted with a larger box size of 500 pixels and refined by non-uniform refinement with C3 symmetry to a resolution of 4.33 Å. The smaller-sized particles were used to obtain a reference template by topaz training from unbinned motion -corrected micrographs (0.651 Å/pixel) with particles extracted with a box size of 500 px. The final map was generated from 208,727 particles and refined to 2.95 Å, containing one hexameric channel but missing the LRRD density after non-uniform refinement and 3D classification. To investigate the structure of the individual channels in the trimeric assembly, 538,894 particles of the channel trimer were re-extracted with a small box size of 500 pixels from unbin micrographs (0.651 Å/pixel) and trained by Topaz train as a reference. All particles were divided into three different classes: 204,758 particles with visible LRR domains, 257,619 particles with a pore domain but unresolved LRR domains, and the rest containing a class of junk particles. The class with a single resolved LRR domain was improved to 2.78 Å from 124,661 particles after 3D classification and non-uniform refinement with C1 symmetry. The map of the pore domain was polished to 2.65 Å using local and global CTF refinement with C1 symmetry.

### Model building and refinement

For model building of 8A-L402W, the structure of hLRRC8A (PDB: 7XZH) was placed into the density of the better resolved map of the mutant obtained in the absence of ATP, manually rebuilt in Coot^58^ to match the altered conformation and refined in Phenix^59^. This model was used to refine the highly similar structure of L402W in the presence of ATP, and the model of the single hexamer of the double mutant W168L/L402W lacking the LRRDs and part of the ISD. For the modeling of the channel trimer at lower resolution (4.33 Å), the structure of hLRRC8A was rebuilt to match the altered LRRD conformation of this construct in Coot and refined in Phenix. Figures were generated using UCSF ChimeraX^60^ and Dino (http://www.dino3d.org).

### Reporting summary

Further information on research design is available in the Nature Portfolio Reporting Summary linked to this article.

## Supplementary information

Supplementary Information

Reporting Summary

Transparent Peer Review file

## Source data

Source Data

## Data Availability

The three-dimensional cryo-EM density maps have been deposited in the Electron Microscopy Data Bank under accession numbers EMD-55484 (LRRC8A-L402W), EMD-55483 (LRRC8A-L402W with ATP), EMD-57359 (LRRC8A-I2C-L402W), EMD-55482 (LRRC8A-W168L-L402W) and EMD-55481 (LRRC8A-W168L-L402W channel trimer). The deposition includes maps of full-length proteins, corresponding half-maps 1 and 2, the mask used for final FSC calculation, as well as relevant higher resolution maps obtained after local refinement. Coordinates for the models of full-length LRRC8A-L402W, LRRC8A-L402W in the presence of ATP, LRRC8A-I2C-L402W and LRRC8A-W168L-L402W have been deposited in the Protein Data Bank under accession numbers 9T35 https://doi.org/10.2210/pdb9T35/pdb, 9T34 https://doi.org/10.2210/pdb9T34/pdb, 29TC https://doi.org/10.2210/pdb29TC/pdb and 9T33 https://doi.org/10.2210/pdb9T33/pdb, respectively. All other data needed to evaluate the conclusions of the study are present in the paper or in the supplementary materials. Source data are provided with this paper.
