# Supplementary material for: The intracellular subdomain of the volume-regulated anion channel subunit LRRC8A is a hotspot of channel activation
Source: Nat Commun. 2026 Sep 17;17:9885. doi: 10.1038/s41467-026-77507-y (PMC13586329; doi:10.1038/s41467-026-77507-y)
Supplement: Supplementary file 1 [file 41467_2026_77507_MOESM1_ESM.pdf]

Supplementary Information

The intracellular subdomain of the volume-regulated anion channel subunit LRRC8A is a hotspot of channel activation

Sara Bertelli, Lu Wang, Malte Klüssendorf, Paola Gavazzo, Michael Pusch, Raimund Dutzler and Tobias Stauber

Supplementary Table 1: Mutagenesis primers

I2C-fwd	CTT AAC CAT GTG TCC GGT GAC AGA GCT CC
I2C-rev	GTC ACC GGA CAC ATG GTT AAG CCT GC
E6A-fwd	GGT GAC AGC GCT CCG CTA CTT TGC GGA CAC G
E6A-rev	GCG GAG CGC TGT CAC CGG AAT CAT GG
W168L-fwd	GAA GTG CTT CGA CTC GCC CCT GAC CAC GAG GGC CCT GT
W168L-rev	CCC TCG TGG TCA GGG GCG AGT CGA AGC ACT TCA GCA GGA T
V392L-fwd	TCC AAG CGC TTC GCC CTC TTC CTG TC
V392L-rev	GGG CGA AGC GCT TGG AGT AGA GCG GG
V392W-fwd	CTT CGC CTG GTT CCT GTC GGA GGT GA
V392W-rev	ACA GGA ACC AGG CGA AGC GCT TGG AG
L402A-fwd	GAA CAA GGC GCG GCA GCT GAA CCT C
L402A-rev	CTG CCG CGC CTT GTT CTC ACT CAC C
L402F-fwd	GAA CAA GTT TCG GCA GCT GAA CCT CA
L402F-rev	GCT GCC GAA ACT TGT TCT CAC TCA CC
L402I-fwd	GAA CAA GAT TCG GCA GCT GAA CCT CA
L402I-rev	GCT GCC GAA TCT TGT TCT CAC TCA CC
L402W-fwd	GAG AAC AAG TGG CGG CAG CTG AAC CTC AAC
L402W-rev	GCT GCC GCC ACT TGT TCT CAC TCA CCT
L402W-exHA fwd	GAG TGA GAA CAA GTG GCG GCA GCT GAA CCT C
L402W-exHA rev	GAG GTT CAG CTG CCG CCA CTT GTT CTC ACT C
8A-cHA-fwd	TTT TTG AAT TCC ATG ATT CCG G
8A-cHA-rev	AAA AAG GTA CCT CAG GCA TAA TCT GGG ACG TCA TAA GGG TAG GCC TGC TCC TTG TCA GC

Supplementary Figures

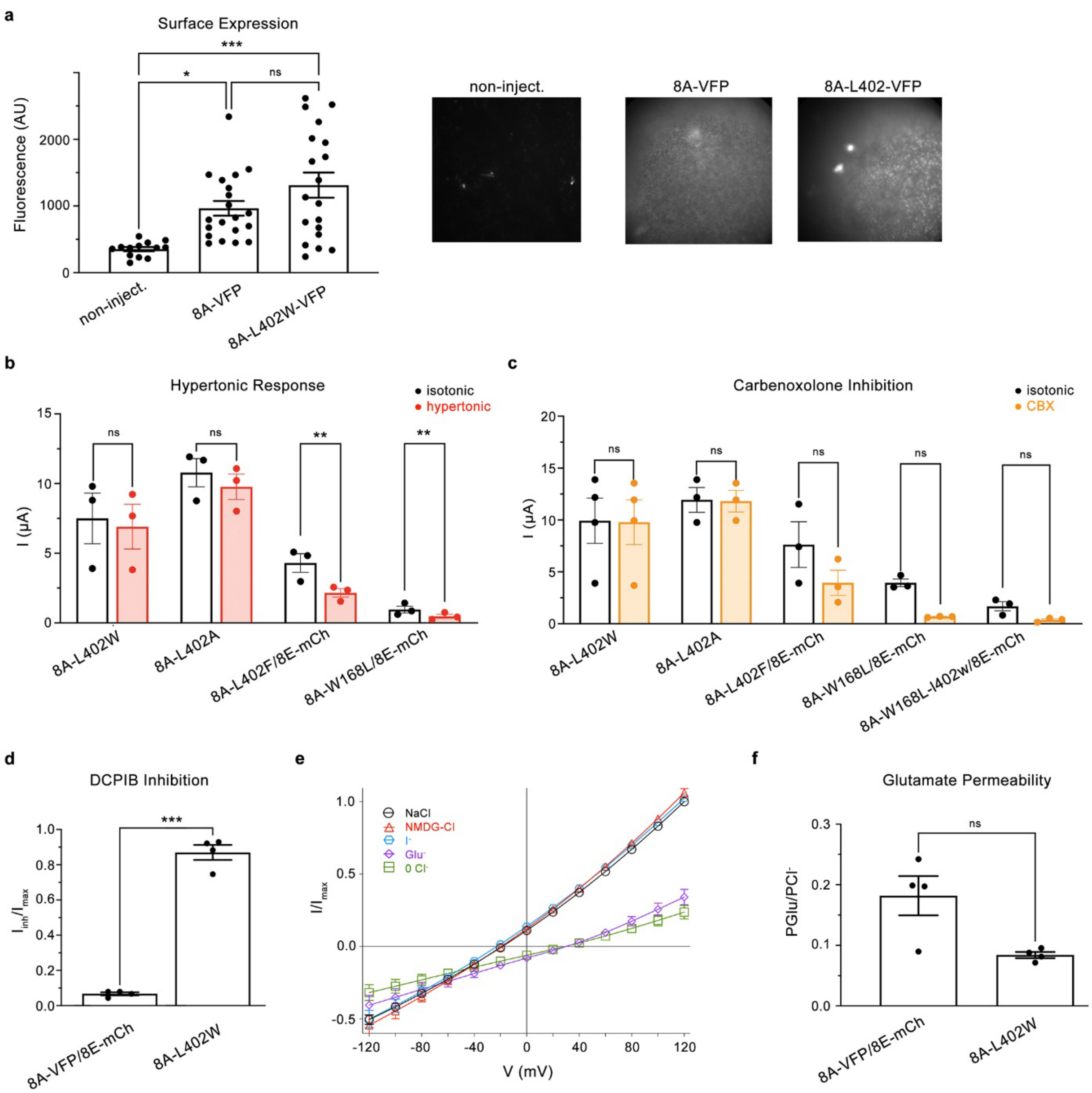

Supplementary Fig. 1: Properties of LRRC8 currents. a, Plasma membrane expression levels of LRRC8A-Venus fusion proteins in *X. laevis* oocytes assayed by the fluorescence at the cell surface. Left, mean VFP-fluorescence of indicated fusion constructs. While the difference between non-injected (n = 13) and either 8A-VFP-injected (n = 20) or 8A-L402W-injected (n = 19) oocytes is statistically significant (p = 0.0142 and 0.0001, respectively), the difference between 8A-VFP-injected or 8A-L402W-injected oocytes is not statistically significant (p = 0.1631), as determined by one-way ANOVA followed by Tukey´s post-hoc test. Right, fluorescence microscopy images of representative oocytes expressing these constructs. **b**, Sensitivity of basal LRRC8-mediated currents to hypertonicity. Mean values of currents of cells injected with

the indicated constructs at +60 mV upon and 280 s after replacing isotonic by hypertonic buffer. **c**, Sensitivity of basal LRRC8-mediated currents to carbenoxolone (CBX). Mean values of currents of cells injected with the indicated constructs at +60 mV and 280 s after application of CBX. **b**, **c**, Currents of 8A-L402W and 8A-L402A were not significantly affected by hypertonicity (panel b; p = 0.1587 and 0.0801, n = 3) or CBX (panel c; p = 0.1494 and 0.6291, n = 4 and 3). Currents of 8A-L402F/8E-mCh were significantly reduced by hypertonicity (panel b, p = 0.0038, n = 3) but not by CBX (panel c, p = 0.0030, n = 3), whereas currents of 8A-W168L/8E-mCh were significantly reduced by hypertonicity (panel b, p = 0.0074, n = 3) and by CBX (panel c, p = 0.0027, n = 3). Currents of 8A-W168L-L402W/8E-mCh were significantly reduced by CBX (panel c, p = 0.0014, n = 3). **d**, Inhibition of indicated LRRC8 constructs upon addition of 100 μM of DCPIB. Plotted is the normalized current decrease after inhibitor addition. The difference between the inhibition of 8A-VFP/8E-mCh and 8A-L402W is statistically significant (p = 0.0005, n = 4). **e**, Anion selectivity of LRRC8A-L402W as measured from current-voltage relationships at specified extracellular solutions showing pronounced differences in the currents and a shift in the reversal potential that demonstrate that the recorded currents are mediated by Cl^-. **f**, Apparent permeability ratio between Glu^- and Cl^- for indicated constructs. The difference is statistically significant (p = 0.0741, n=4). **b**-**d**, **f**, Statistical significance was assessed by a two-tailed paired ratio t-test. **a**-**f**, Errors are s.e.m.

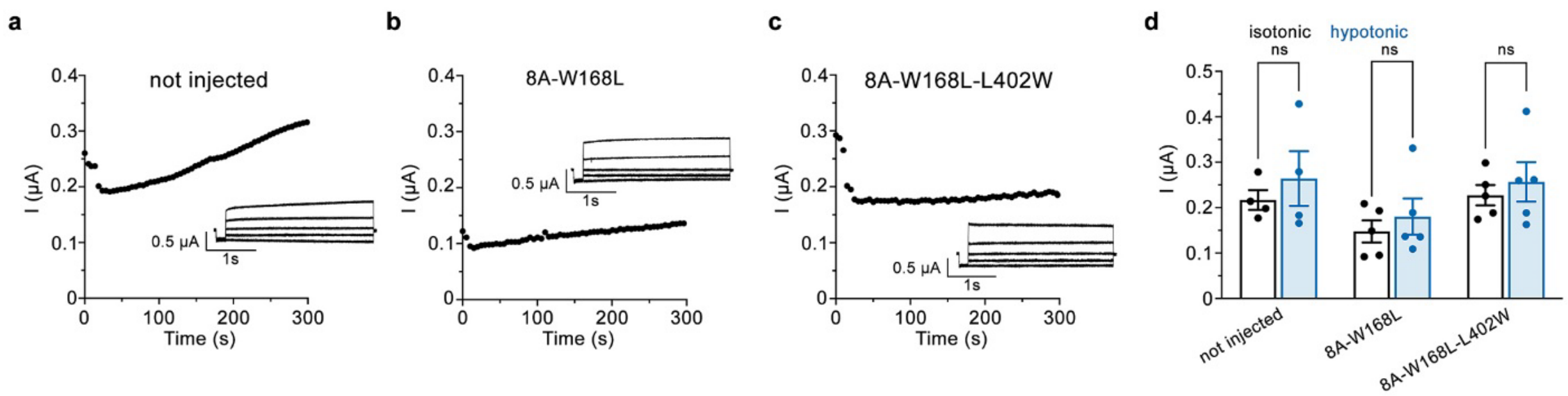

Supplementary Fig. 2: Low current response by homomeric constructs of the mutants 8A-W168L and 8A-W168L-L402W. a-c, Current response to a repetitive pulse to +60 mV applied at 0.2 Hz of representative cells of, **a**, non-injected *X. laevis* oocytes, or oocytes injected with, **b**, the constructs 8A-W168L or, **c**, 8A-W168L-L402W to a hypotonic stimulus. The initial drop of currents is due to the lower Cl^- concentration in the hypotonic solution. Insets show current traces of the same oocytes in response to an IV-pulse protocol. **d**, Mean values of currents at +60 mV before and after ~280 s of hypotonic stimulation. Individual experiments are shown as dots, errors are s.e.m. Statistical significance was evaluated using a two-tailed paired ratio t-test. Hypotonic stimulation did not significantly affect the currents of non-injected oocytes (ni; p = 0.5127, n = 4), 8A-W168L (p = 0.3396, n = 5), or 8A-W168L-L402W (p = 0.3127, n = 5).

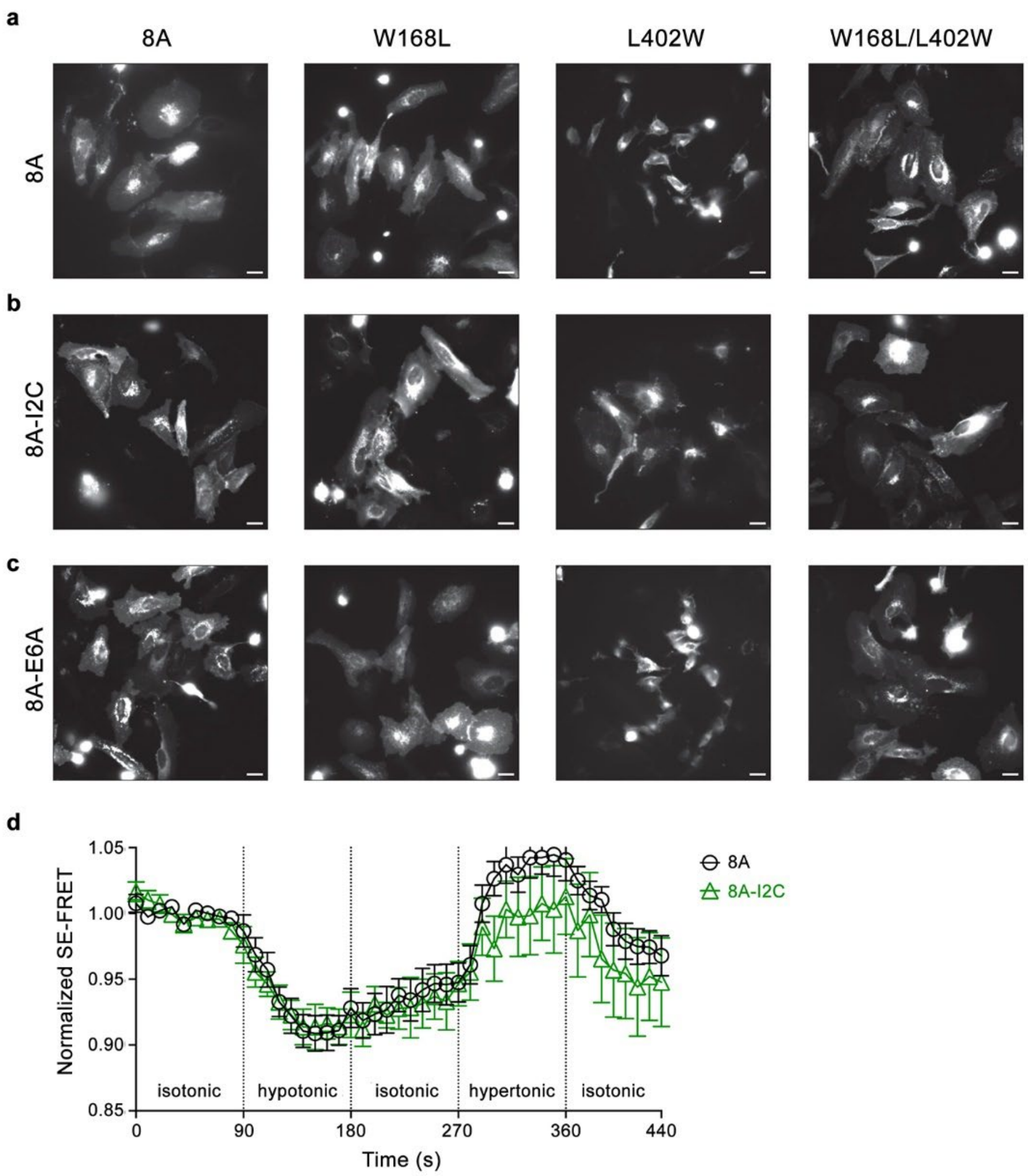

Supplementary Fig. 3: Mutant expression for FRET experiments. a-c, Representative microscopy images of the donor channel of HeLa cells transfected with FRET pairs (mCerulean3 as donor and mVenus as acceptor) for, **a**, LRRC8A (8A), 8A-W168L, 8A-L402W and 8A-W168L-L402W or, **b**, with pairs also containing the additional mutation I2C or, **c**, E6A. Scale bars, 20 µm. **d,** Hela cells transfected with FRET pairs for 8A or 8A-I2C and imaged over time with an interval of 10 s, after changing the surrounding solution by buffers with different tonicities for 90 s as indicated (isotonic: 340 mOsm; hypotonic: 240 mOsm; hypertonic: 500 mOsm). Traces show the calculated SE-FRET values normalized to the mean SE-FRET value of the first 90 s in isotonic buffer. n=6 fields of view with different numbers of cells from 3 independent experiments.

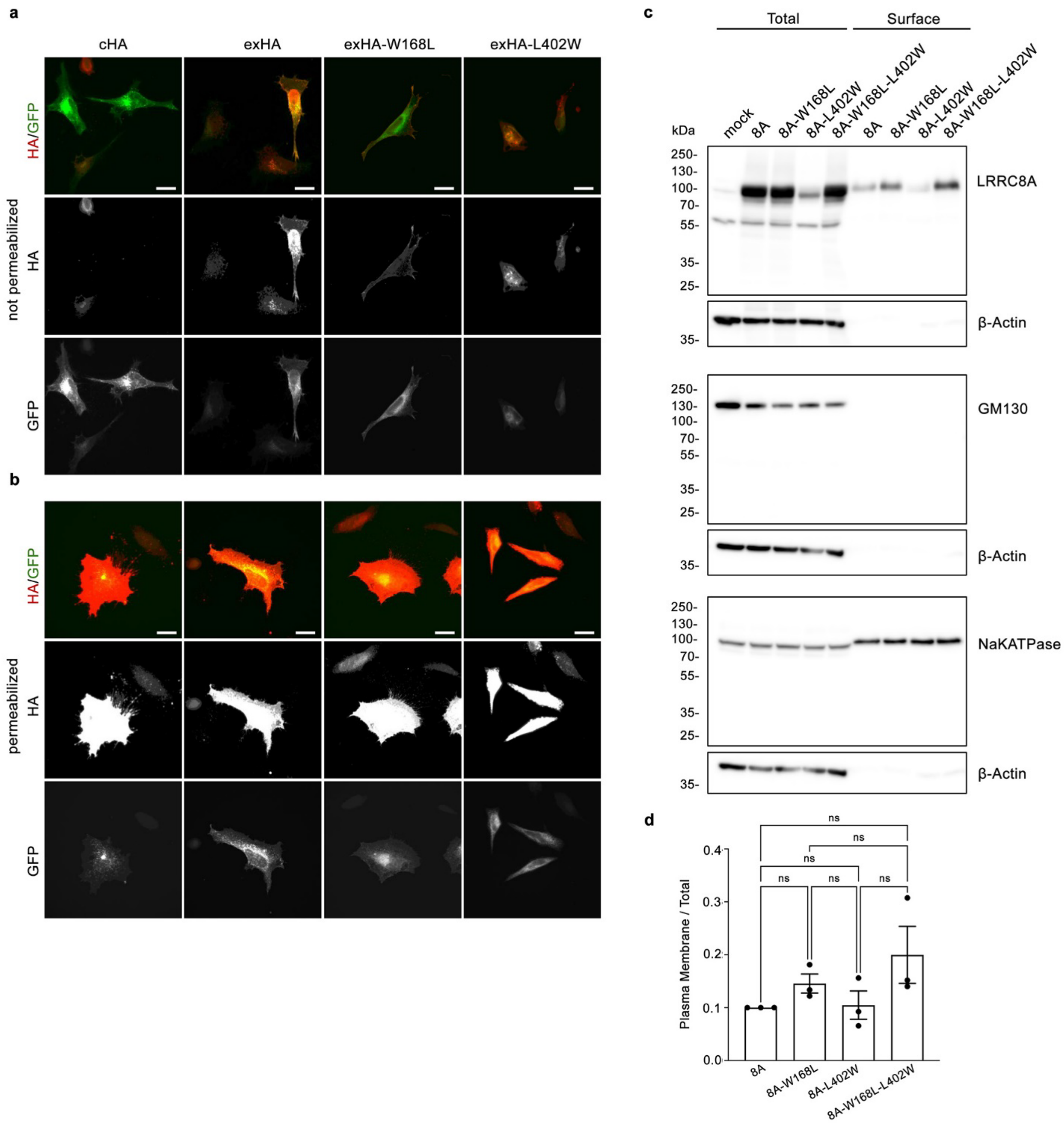

Supplementary Fig. 4: Plasma membrane expression of LRRC8A variants assessed by HA immunostaining and cell surface biotinylation. a, Representative microscopy images of HeLa cells transfected with a GFP-LRRC8A constructs containing a cytoplasmic HA tag fused to the C-terminus (cHA), or LRRC8A variants (WT, W168L or L402W) carrying an extracytosolic HA tag in the loop between TM3 and 4. The cells were stained with an antibody against the HA tag either in, **a**, untreated cells, or **b**, after detergent permeabilization. Note the anti-HA signal for all cells expressing LRRC8A variants with an extracytosolic HA tag, but not with the C-terminal HA tag in non-permeabilized cells. Scale bars, 20 μm. **c,** Representative Western blots of the biotinylated fraction (surface) and total protein (total) of LRRC8A

knockout HeLa cells transfected with the LRRC8A variants WT, W168L, L402W or W168L-L402W. The membranes were probed with antibodies against LRRC8A, the Golgi-localized peripheral membrane protein GM130 and the plasma membrane Na/K-ATPase. β-actin served as loading control. Total protein of cells transfected without plasmid DNA (mock) shows LRRC8A antibody specificity. Note the reduced levels of 8A-L402W, which is consistent with its toxic effect on expressing cells. **d,** Quantification of relative cell surface expression from three independent experiments as in **c**. Values are shown as mean ± s.e.m. from three independent experiments. No statistical significance was observed by one-way ANOVA followed by Tukey´s post-hoc test.

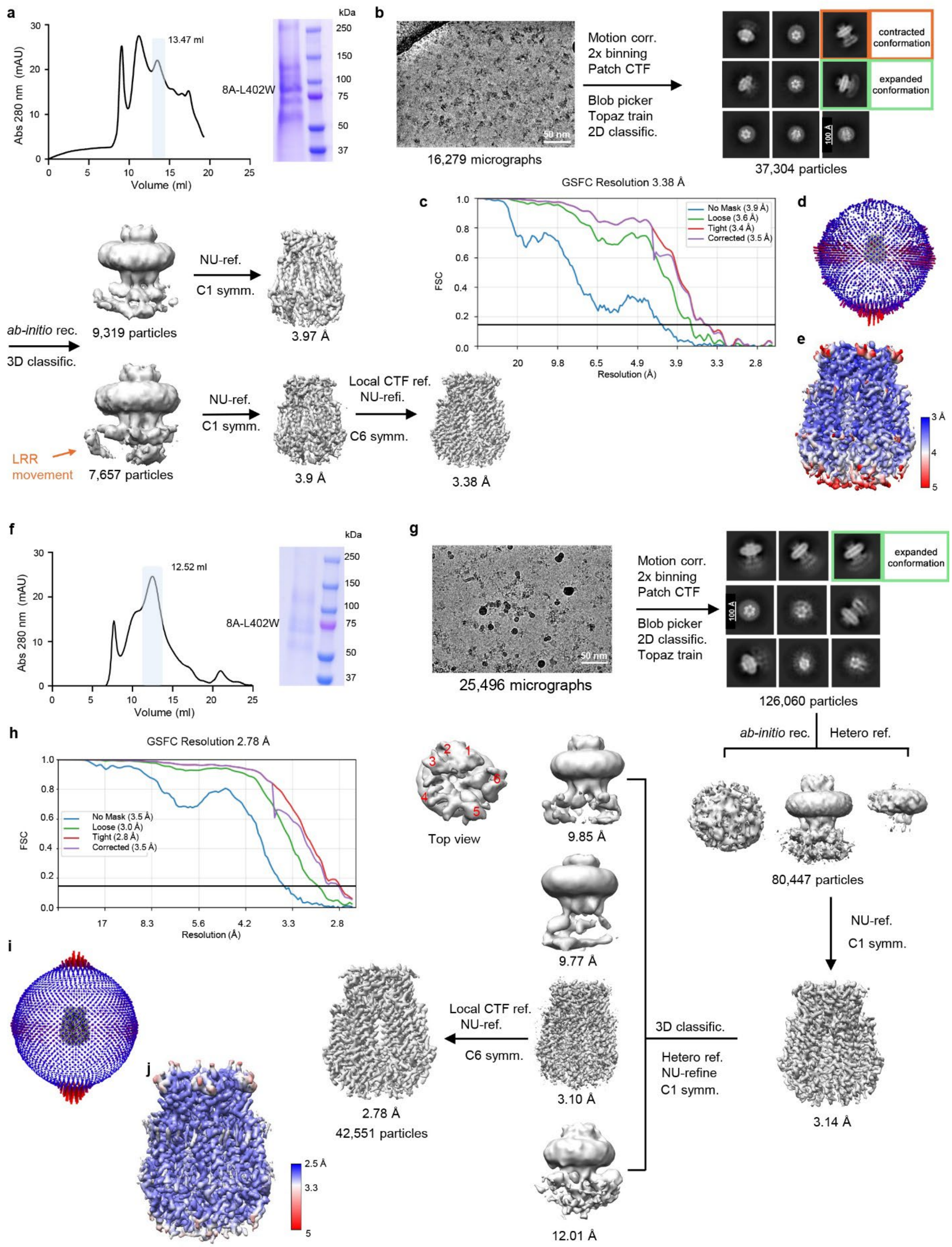
a
13.47 ml
Abs 280 nm (mAU)
Volume (ml)
8A-L402W
kDa
b
Motion corr.
2x binning
Patch CTF
Blob picker
Topaz train
2D classific.
16,279 micrographs
contracted conformation
expanded conformation
37,304 particles
GSFC Resolution 3.38 Å
c
No Mask (3.9 Å)
Loose (3.6 Å)
Tight (3.4 Å)
Corrected (3.5 Å)
FSC
Resolution (Å)
d
e
ab-initio rec.
3D classific.
NU-ref.
C1 symm.
9,319 particles
3.97 Å
LRR movement
7,657 particles
3.9 Å
Local CTF ref.
NU-refi.
C6 symm.
3.38 Å
f
12.52 ml
Abs 280 nm (mAU)
Volume (ml)
8A-L402W
kDa
g
Motion corr.
2x binning
Patch CTF
Blob picker
2D classific.
Topaz train
25,496 micrographs
expanded conformation
126,060 particles
h
GSFC Resolution 2.78 Å
No Mask (3.5 Å)
Loose (3.0 Å)
Tight (2.8 Å)
Corrected (3.5 Å)
FSC
Resolution (Å)
Top view
9.85 Å
9.77 Å
3.10 Å
12.01 Å
ab-initio rec.
Hetero ref.
80,447 particles
NU-ref.
C1 symm.
3.14 Å
3D classific.
Hetero ref.
NU-refine
C1 symm.
Local CTF ref.
NU-ref.
C6 symm.
2.78 Å
42,551 particles
i
j

Supplementary Fig. 5: Data processing strategy for the LRRC8A mutant L402W in presence and absence of ATP. **a**, Size exclusion chromatography (SEC) trace of a sample of the mutant 8A-L402W in presence of ATP (left) and SDS-PAGE gel of the peak fraction (indicated by blue box on the chromatogram). **b,** Representative image and workflow of cryo-EM data processing. Representative 2D class averages. Expanded and contracted LRRD conformations are marked in green and orange, respectively. Low-resolution 3D reconstructions show pronounced movements of LRR domains. **c,** Fourier shell correlation (FSC) plot of the final map. Straight line corresponds to the resolution at which the FSC drops below the 0.143 threshold. **d**, Angular distribution plot of all particles included in the final reconstruction of the structures. **e,** Final 3D reconstruction colored according to local resolution. **f**, Size exclusion chromatography (SEC) trace of a sample of the mutant 8A-L402W in absence of ATP (left) and SDS-PAGE gel of the peak fraction (indicated by blue box on the chromatogram). **g**, Representative image and workflow of cryo-EM data processing. Representative 2D class averages with a class average showing an expanded LRRD domain conformation marked in green. **h**, Fourier shell correlation (FSC) plot of the final map. Straight line corresponds to the resolution at which the FSC drops below the 0.143 threshold. **i**, Angular distribution plot of all particles included in the final reconstruction of the structures. **j**, Final 3D reconstruction colored according to local resolution.

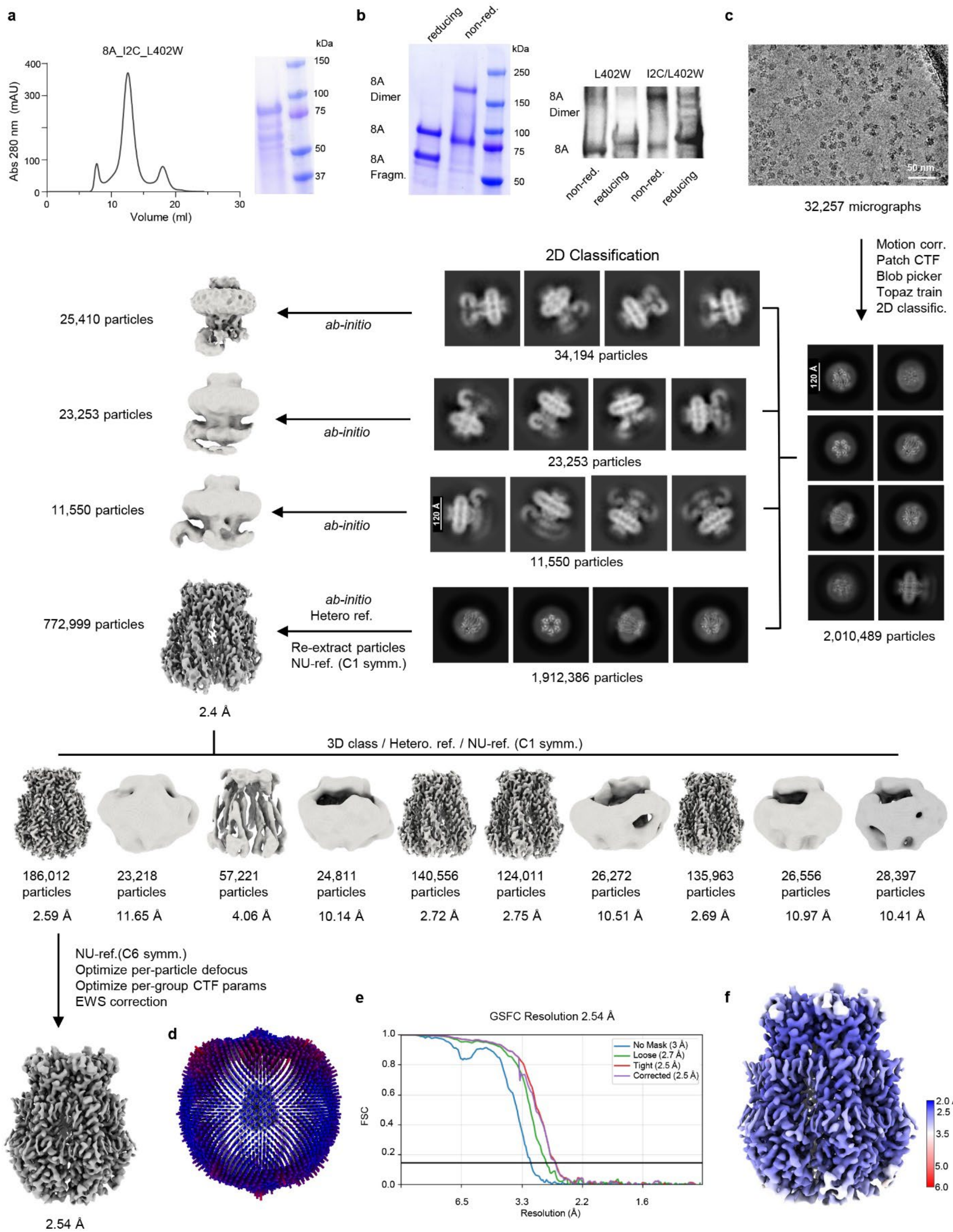
a
8A_I2C_L402W
Abs 280 nm (mAU)
Volume (ml)
kDa
150
100
75
50
37
b
reducing
non-red.
8A Dimer
8A
8A Fragm.
kDa
250
150
100
75
50
L402W
I2C/L402W
8A Dimer
8A
non-red.
reducing
non-red.
reducing
c
50 nm
32,257 micrographs
Motion corr.
Patch CTF
Blob picker
Topaz train
2D classific.
2D Classification
120 Å
2,010,489 particles
25,410 particles
ab-initio
34,194 particles
23,253 particles
ab-initio
23,253 particles
11,550 particles
ab-initio
120 Å
11,550 particles
772,999 particles
ab-initio
Hetero ref.
Re-extract particles
NU-ref. (C1 symm.)
1,912,386 particles
2.4 Å
3D class / Hetero. ref. / NU-ref. (C1 symm.)
186,012 particles
2.59 Å
23,218 particles
11.65 Å
57,221 particles
4.06 Å
24,811 particles
10.14 Å
140,556 particles
2.72 Å
124,011 particles
2.75 Å
26,272 particles
10.51 Å
135,963 particles
2.69 Å
26,556 particles
10.97 Å
28,397 particles
10.41 Å
NU-ref.(C6 symm.)
Optimize per-particle defocus
Optimize per-group CTF params
EWS correction
2.54 Å
d
e
GSFC Resolution 2.54 Å
FSC
No Mask (3 Å)
Loose (2.7 Å)
Tight (2.5 Å)
Corrected (2.5 Å)
Resolution (Å)
6.5
3.3
2.2
1.6
f
2.0 Å
2.5
3.5
5.0
6.0

Supplementary Fig. 6: Data processing strategy for the LRRC8A double mutant I2C-L402W. a, Size exclusion chromatography (SEC) traces of 8A-I2C-L402W (left) and SDS-PAGE gel of the peak fraction (right). **b**, Cys-crosslink between subunits via the mutated N-terminus. Left, SDS PAGE of the purified construct 8A-I2C-L402W under reducing and non-reducing conditions. A band at higher molecular weight obtained under non-reducing conditions (8A dimer) indicates crosslinking between two subunits. The band at lower molecular weight (A8 fragm.) corresponds to the larger C-terminal fragment in a fraction of subunits containing a cleavage in the extended intracellular loop connecting TM2 and 3 as confirmed by mass spectrometry, which appears upon reduction of intra-subunit disulfide bridges. Right, Western blot shows formation of an inter-subunit crosslink in 8A-I2C-L402W but not in 8A-L402W. **c**, Representative cryo-EM image and workflow of the cryo-EM data processing. Low-resolution 3D reconstructions from a comparably small number of particles with preferred side view orientation show pronounced movements of LRR domains. The majority of particles containing top and side views with no defined LRR domain density allowed a reconstruction of the PD at high resolution, whose quality suffered from preferred particle orientation. Further classification resulted in a well-defined density of the PD, which after application of C6 symmetry reached 2.5 Å. **d**, Angular distribution plot of all particles included in the final reconstruction of the structure of the I2C-L402W channels with C6 symmetry. **e**, Fourier shell correlation (FSC) plot of the final map. Straight line corresponds to the resolution at which the FSC drops below the 0.143 threshold. **f**, Final 3D reconstruction colored according to local resolution.

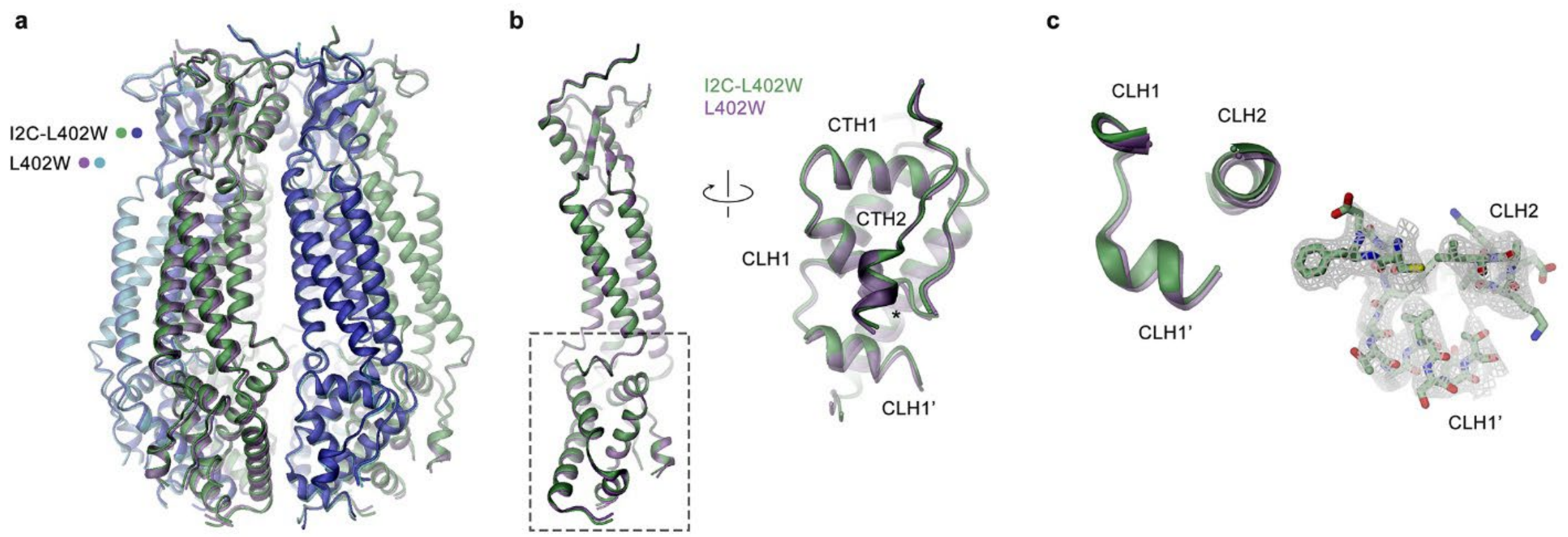

Supplementary Fig. 7: Comparison of the Structures of 8A-L402W and 8A-I2C-L402W. a, Superposition of the refined structures of the mutants 8A-I2C-L402W and 8A-L402W. The PDs of both proteins are shown as ribbons whereas the LRRD is not defined. **b**, Single subunit of the superposition shown in **a** with the ISD (box) enlarged and shown as blowup in indicated orientation (right). **c**, Left, superposition of the region of the ISD of 8A-I2C-L402W and L402W whose conformation has changed compared to WT. Right, stick model of the refined structure of the same region of 8A-I2C-L402W with cryo-EM density (at 2.54 Å) superimposed as mesh.

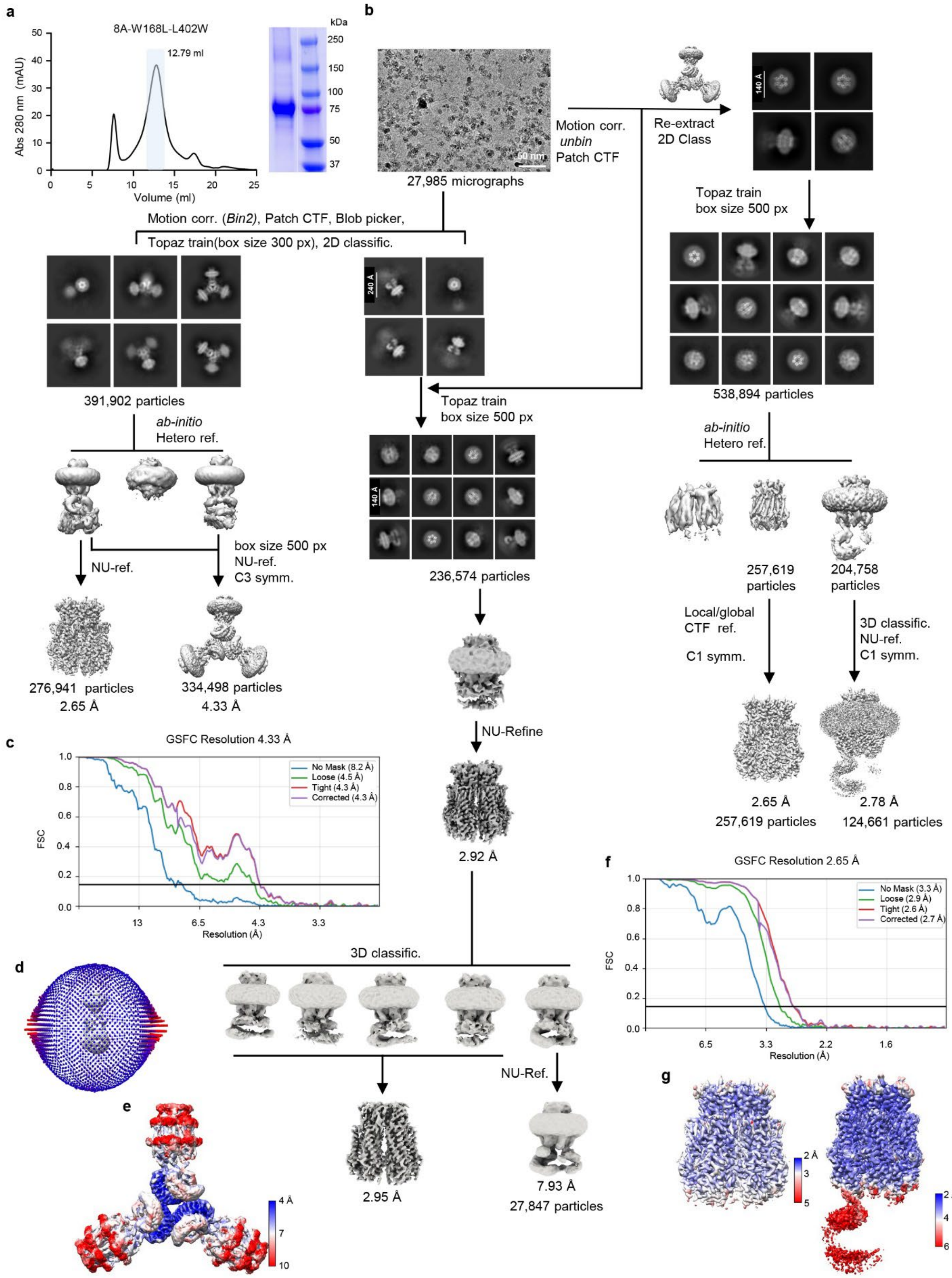
a
8A-W168L-L402W
12.79 ml
Abs 280 nm (mAU)
Volume (ml)
kDa
250
150
100
75
50
37
b
50 nm
27,985 micrographs
Motion corr.
unbin
Patch CTF
Re-extract
2D Class
140 Å
Topaz train
box size 500 px
Motion corr. (Bin2), Patch CTF, Blob picker,
Topaz train(box size 300 px), 2D classific.
240 Å
391,902 particles
ab-initio
Hetero ref.
Topaz train
box size 500 px
538,894 particles
ab-initio
Hetero ref.
box size 500 px
NU-ref.
C3 symm.
NU-ref.
236,574 particles
257,619 particles
204,758 particles
Local/global CTF ref.
C1 symm.
3D classific.
NU-ref.
C1 symm.
276,941 particles
2.65 Å
334,498 particles
4.33 Å
NU-Refine
2.92 Å
2.65 Å
257,619 particles
2.78 Å
124,661 particles
c
GSFC Resolution 4.33 Å
No Mask (8.2 Å)
Loose (4.5 Å)
Tight (4.3 Å)
Corrected (4.3 Å)
FSC
Resolution (Å)
f
GSFC Resolution 2.65 Å
No Mask (3.3 Å)
Loose (2.9 Å)
Tight (2.6 Å)
Corrected (2.7 Å)
3D classific.
d
NU-Ref.
e
2.95 Å
7.93 Å
27,847 particles
g

Supplementary Fig. 8: Data processing strategy for the LRRC8A double mutant W168L-L402W. a, Size exclusion chromatography (SEC) traces of 8A-W168L-L402W (left) and SDS-PAGE gel of the peak fraction (right, indicated by blue box on the chromatogram). **b,** Representative cryo-EM image and workflow of the cryo-EM data processing. **c,** Fourier shell correlation (FSC) plot of the final map of the trimeric assembly of W168L-L402W channels. Straight line corresponds to the resolution at which the FSC drops below the 0.143 threshold. **d**, Angular distribution plot of all particles included in the final reconstruction of the structure of the trimeric assembly of W168L-L402W channels. **e,** Final 3D reconstruction colored according to local resolution of the trimeric assembly of W168L-L402W channels. **f,** Fourier shell correlation (FSC) plot of the final map of a single hexameric W168L-L402W channel obtained from the particles of the trimeric assembly. Straight line corresponds to the resolution at which the FSC drops below the 0.143 threshold. **g**, Final 3D reconstruction colored according to local resolution of the two reconstructions of hexameric W168L-L402W channels obtained from particles of the trimeric assembly.

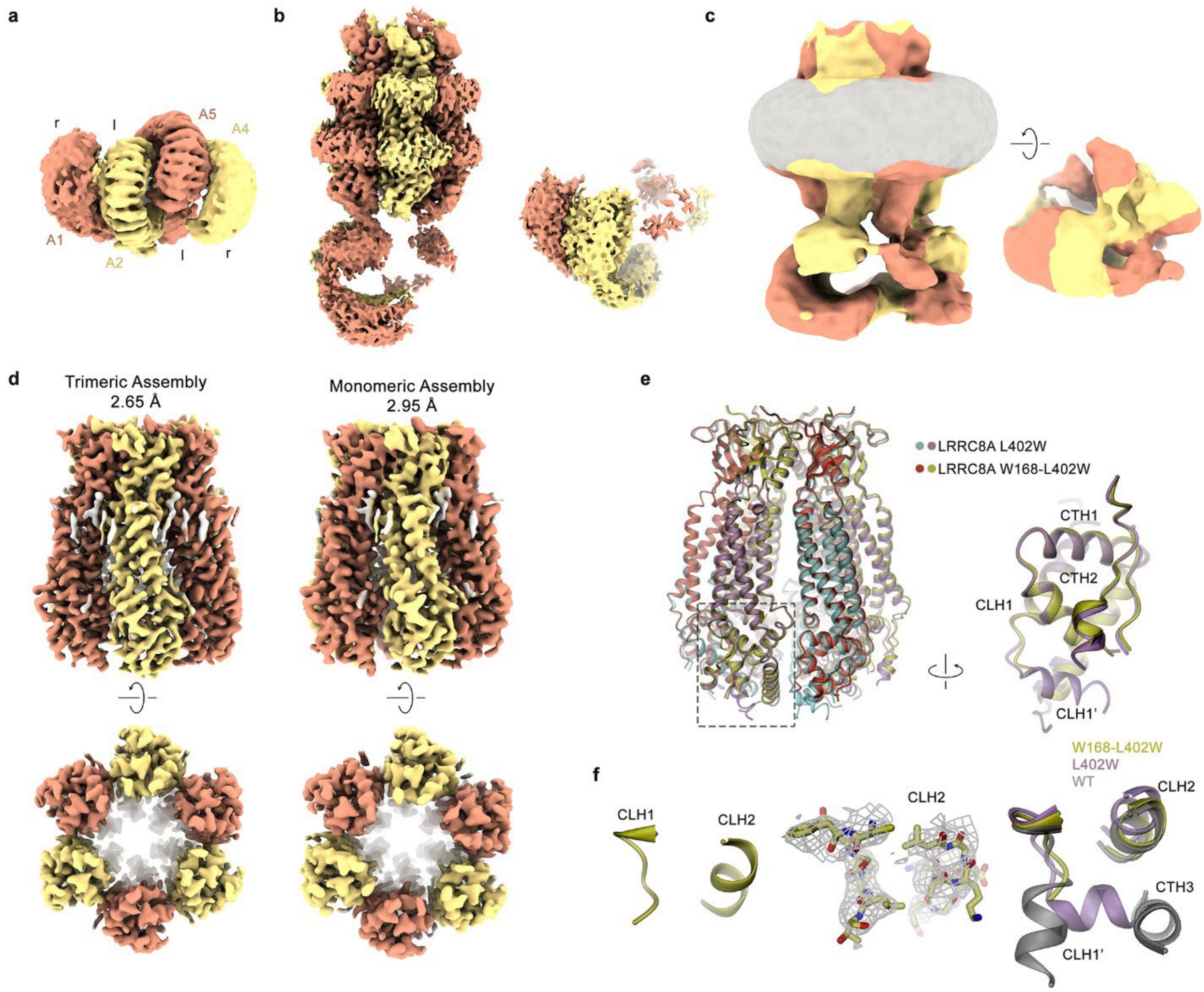

Supplementary Fig. 9: Features of the 8A-W168L-L402W structure. a, Density at 4.33 Å of the LRRD of a single hexameric channel of the trimeric assembly viewed from the cytoplasm. **b**, View from the membrane (left) and from the cytoplasm (right) of the refined density of an extracted hexamer with partially structured LRRDs at 2.78 Å. **c**, 3D class obtained from isolated particles of hexameric channels with partly defined LRRDs at 7.93 Å. **d**, High resolution reconstructions of single hexameric channels obtained after masking of the trimeric assembly (left, at 2.65 Å) and from monomeric particles (right, at 2.95 Å). Both densities show equivalent conformations of the PD with the cytoplasmic LRRDs averaged out. **e**, Superposition of the PDs of the mutants 8A-W168L-L402W and 8A-L402W. Box marks the ISD of one subunit shown as blowup, right. The superposition illustrates the structural similarity of both conformations, except for α-helix CLH1', which is not defined in 8A-W168L-L402W. **f**, Region in the ISD of 8A-W168L-L402W that has changed its conformation in 8A-L402W shown as ribbon (left), as stick model with cryo-EM density superimposed as mesh (center) and as superposition with WT and 8A-L402W.

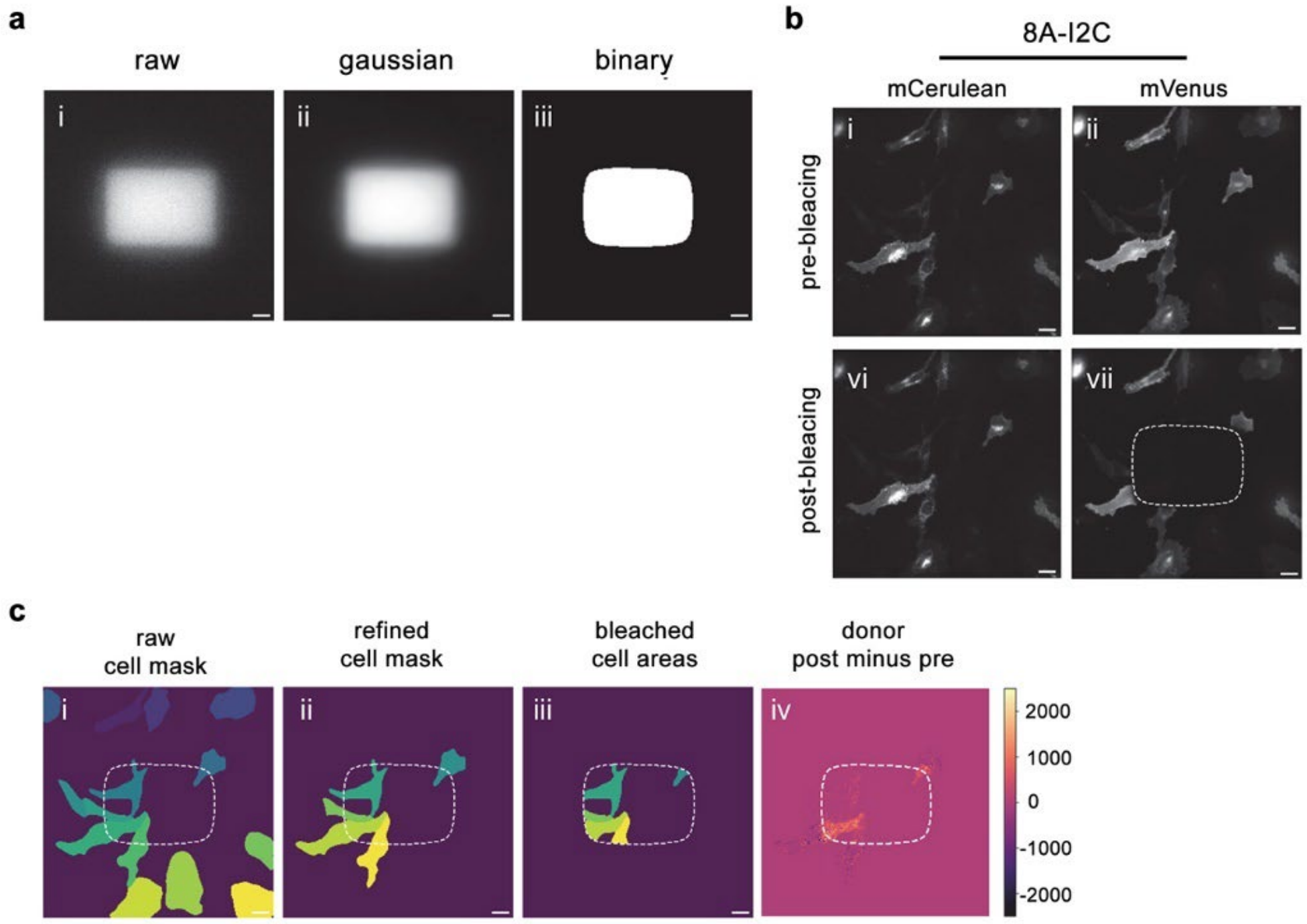

Supplementary Fig. 10: Photo-acceptor bleaching. **a,** Generation of the binary mask defining the bleached area. A gaussian filter was applied to the raw intensity image of the acceptor channel without sample at closed field diaphragm, followed by minimum-thresholding. **b,** Intensity images of the 8A-mCerulean3 (donor) and the 8A-mVenus (acceptor) pre- and post-bleaching. **c**, Cell masks generated by Cellpose showing the processing of cell labels of the donor image (i-iii). Donor channel intensity post-bleaching subtracted by pre-bleaching only for the areas defined by the cell mask shown in ii (iv). Dashed box indicates the bleached area as in a-iii. Scale bars, 20 µm.

SDS-PAGE Gels and Western Blots

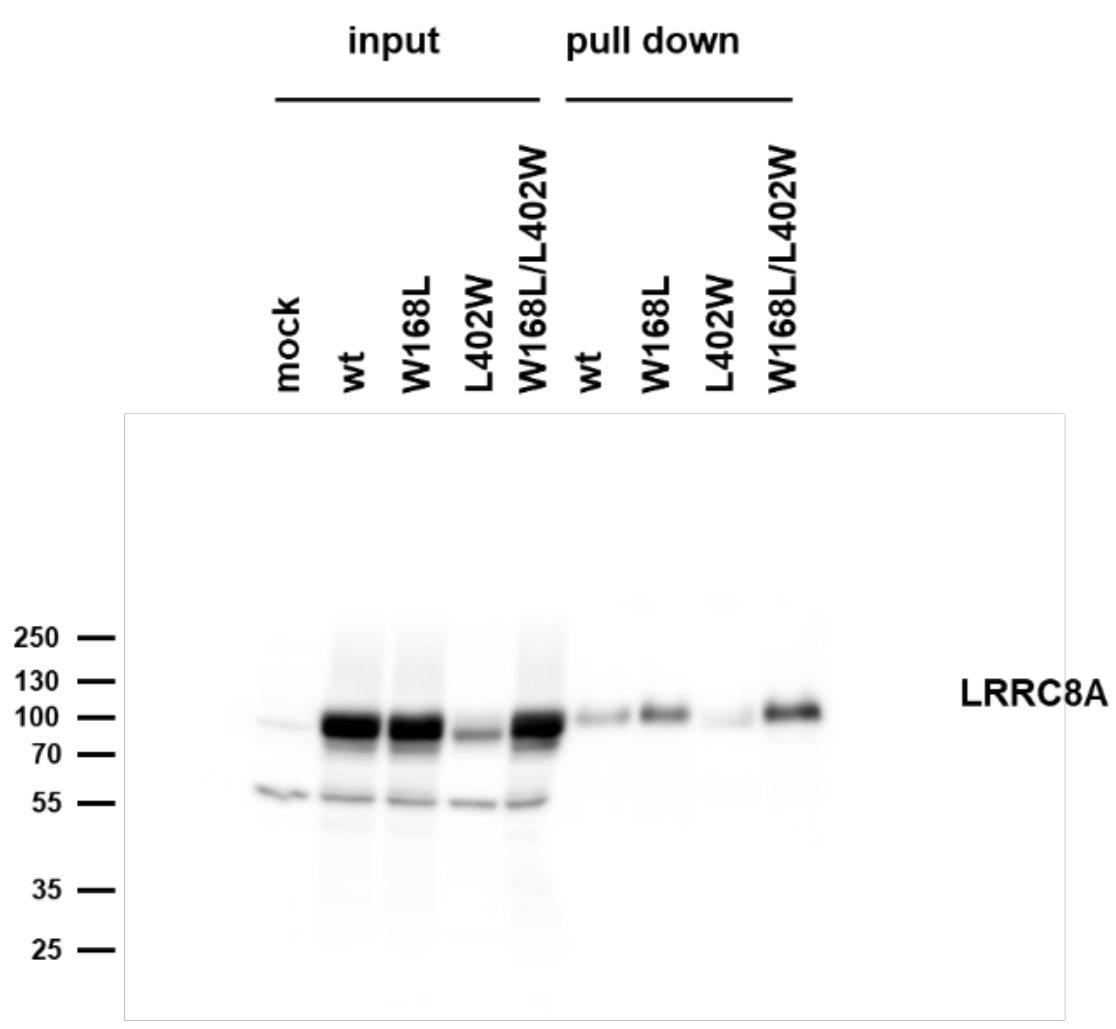

Primary Ab: Anti-LRRC8A produced in rabbit (Voss et al., Science 2014; kindly provided by T. J. Jentsch, diluted 1:500)
Secondary Ab: Goat Anti-Rabbit IgG H&L (HRP) (abcam, #6721, diluted 1:5000)

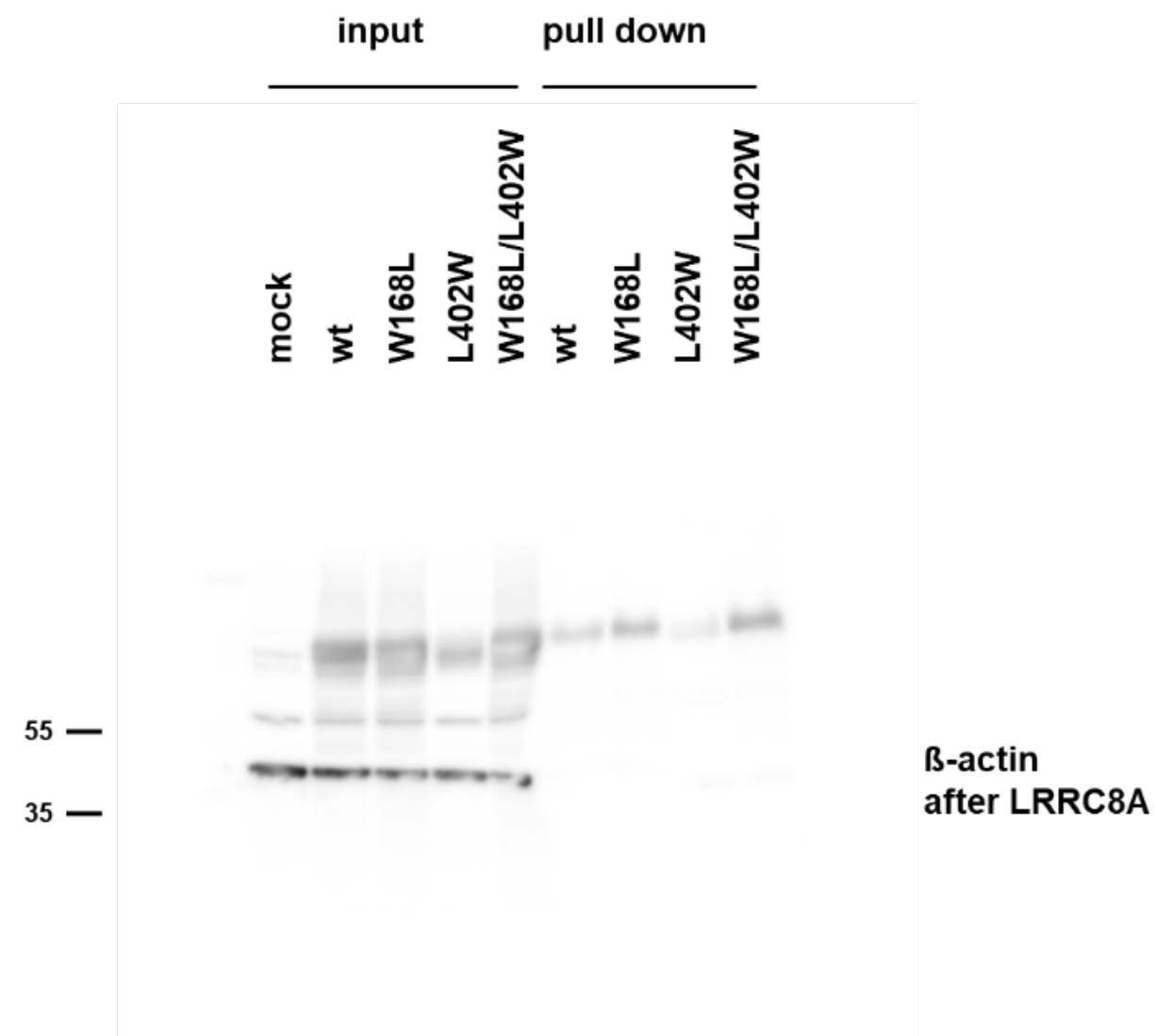

Primary Ab: Anti-ß-actin (abcam, #ab8226, diluted 1:1000)
Secondary Ab: Goat Anti-Mouse IgG H&L (HRP) (abcam, #6789, diluted 1:5000)

Supplementary Fig. 4c top

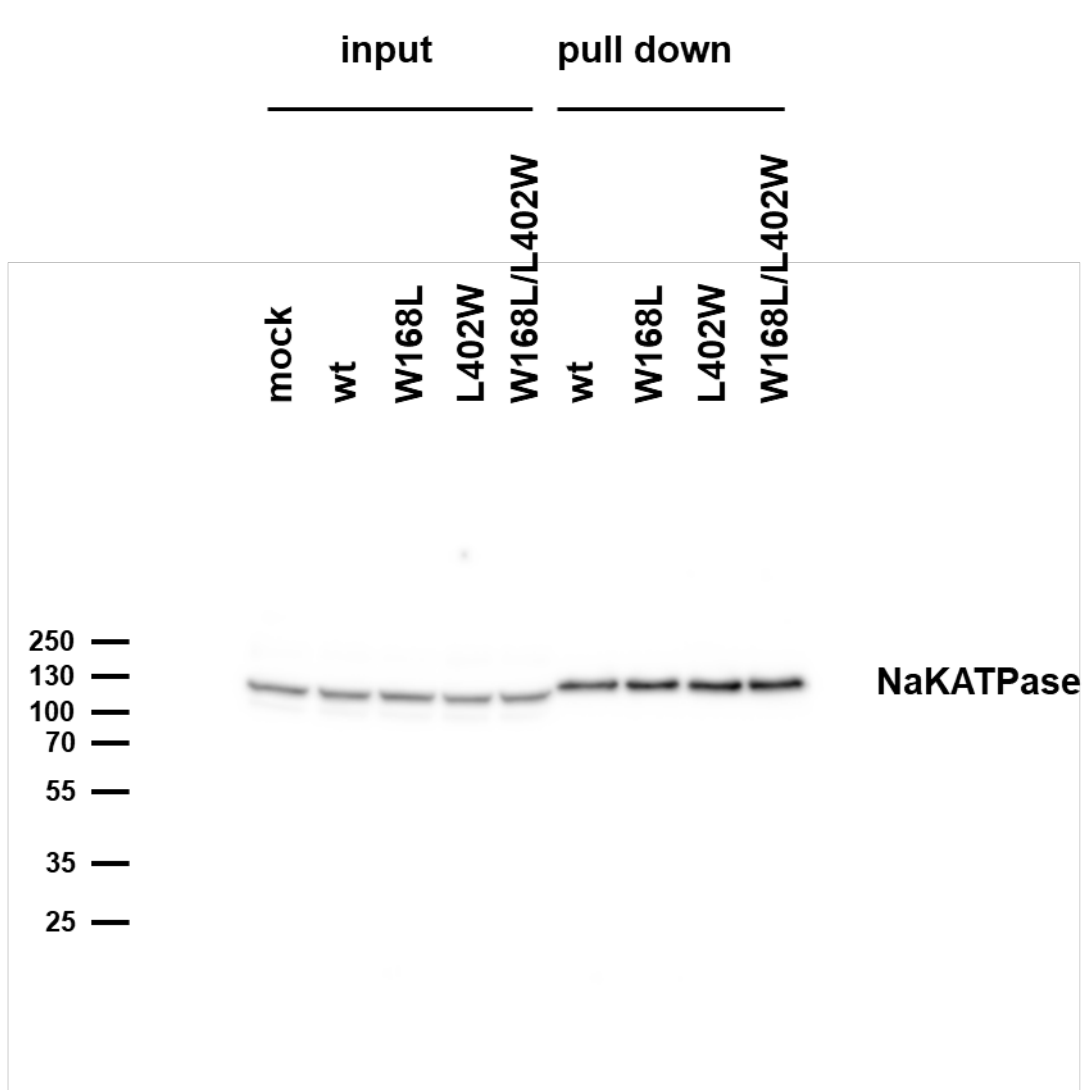

Primary Ab: Monoclonal Anti-Na/K-ATPase a-1 Antibody produced in mouse (Millipore, #05-369, clone C464.6 diluted 1:500)
Secondary Ab: Goat Anti-Mouse IgG H&L (HRP) (abcam, #6789, diluted 1:5000)

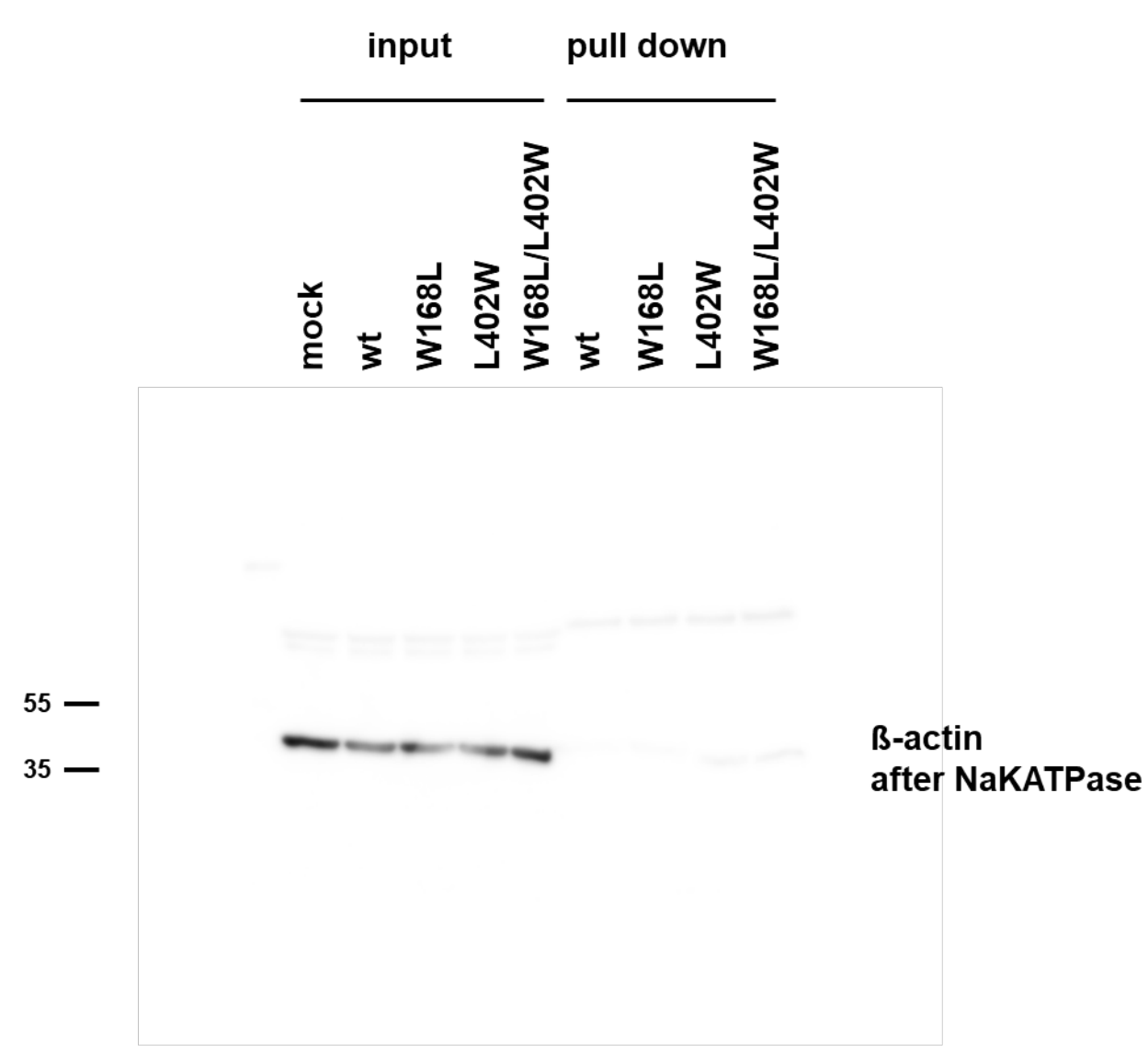

Primary Ab: Anti-ß-actin (abcam, #ab8226, diluted 1:1000)
Secondary Ab: Goat Anti-Mouse IgG H&L (HRP) (abcam, #6789, diluted 1:5000)

Supplementary Fig. 4c center

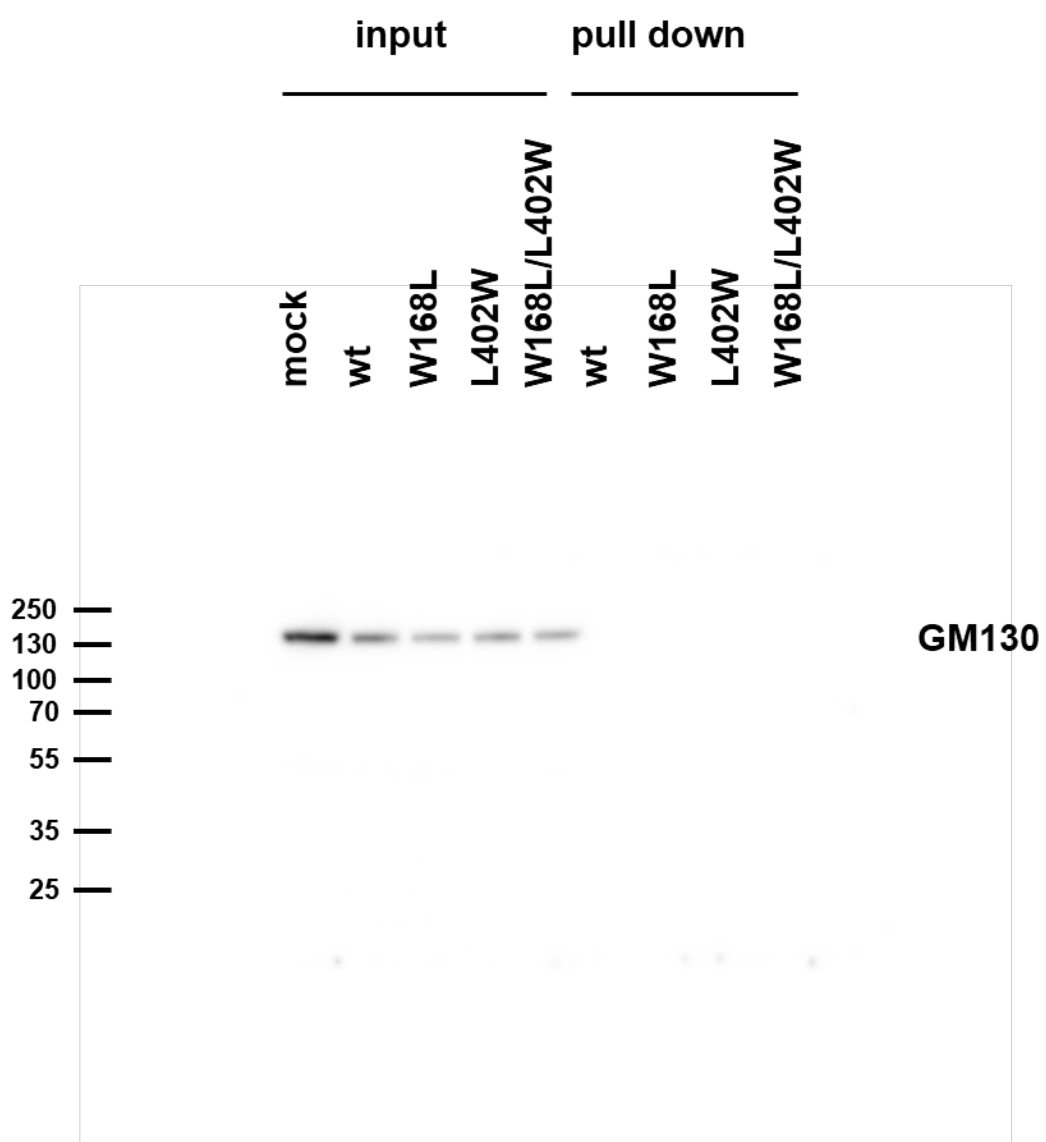

Primary Ab: Anti-GM130 produced in mouse (BD Biosciences, #610823 diluted 1:500)
Secondary Ab: Goat Anti-Mouse IgG H&L (HRP) (abcam, #6789, diluted 1:5000)

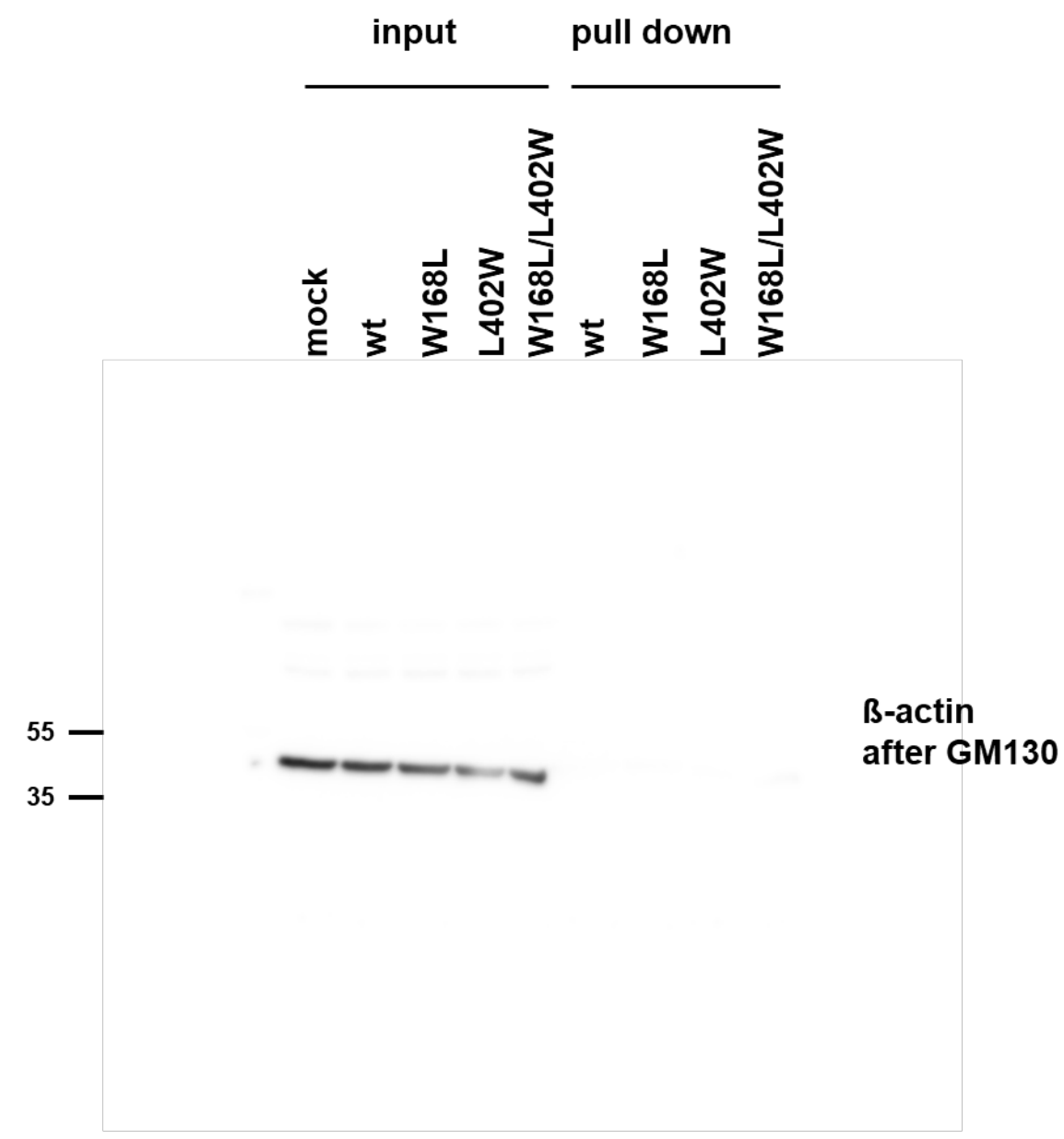

Primary Ab: Anti-ß-actin (abcam, #ab8226, diluted 1:1000)
Secondary Ab:Goat Anti-Mouse IgG H&L (HRP) (abcam, #6789, diluted 1:5000)

Supplementary Fig. 4c bottom

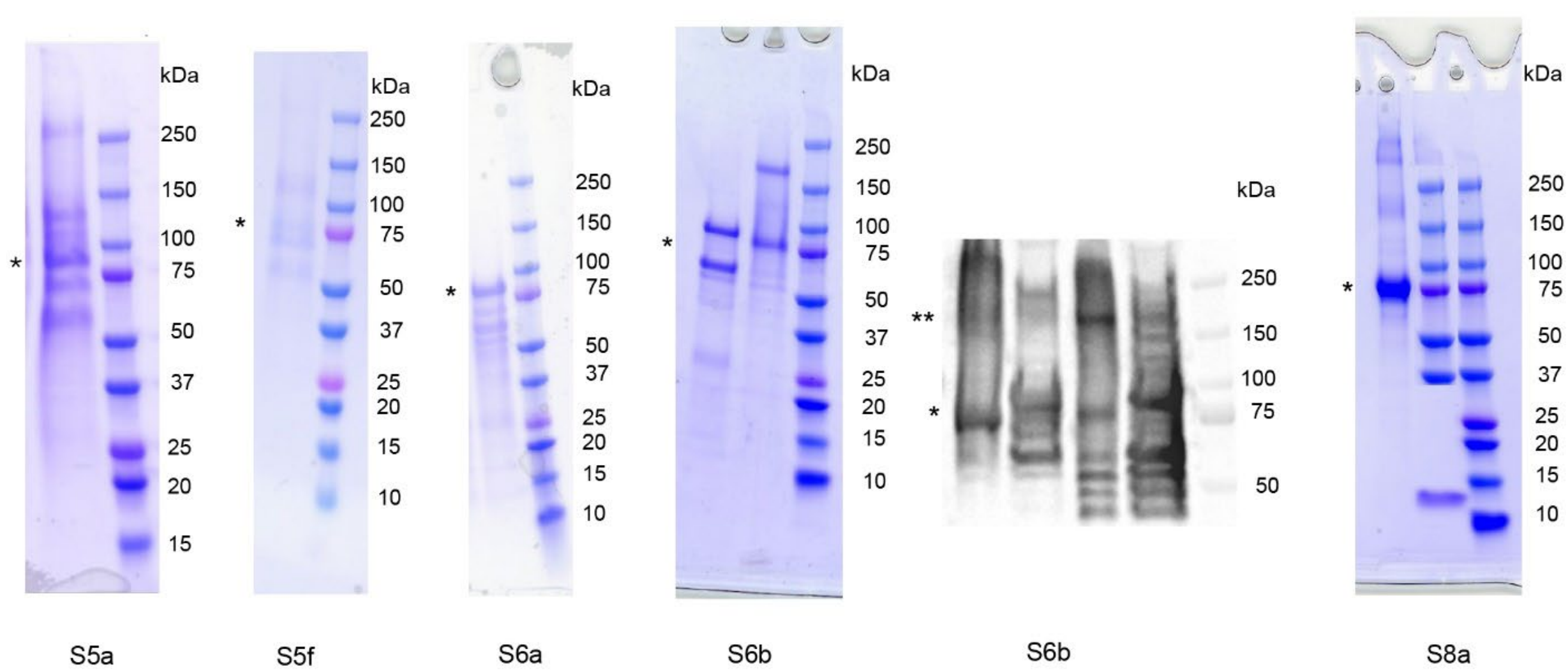

Supplementary Figs. 5a, f, 6a, 6b and 8a
