# Supplementary material for: The intracellular subdomain of the volume-regulated anion channel subunit LRRC8A is a hotspot of channel activation
Source: Nat Commun. 2026 Sep 17;17:9885. doi: 10.1038/s41467-026-77507-y (PMC13586329; doi:10.1038/s41467-026-77507-y)
Supplement: Supplementary file 2 [file 41467_2026_77507_MOESM2_ESM.pdf]

nature portfolio

Corresponding author(s): Michael Pusch, Raimund Dutzler and Tobias Stauber

Last updated by author(s): Aug 6, 2026

Reporting Summary

Nature Portfolio wishes to improve the reproducibility of the work that we publish. This form provides structure for consistency and transparency in reporting. For further information on Nature Portfolio policies, see our Editorial Policies and the Editorial Policy Checklist.

Statistics

For all statistical analyses, confirm that the following items are present in the figure legend, table legend, main text, or Methods section.

n/a	Confirmed	
☐	☒	The exact sample size (n) for each experimental group/condition, given as a discrete number and unit of measurement
☐	☒	A statement on whether measurements were taken from distinct samples or whether the same sample was measured repeatedly
☐	☒	The statistical test(s) used AND whether they are one- or two-sided<br>*Only common tests should be described solely by name; describe more complex techniques in the Methods section.*
☐	☒	A description of all covariates tested
☐	☒	A description of any assumptions or corrections, such as tests of normality and adjustment for multiple comparisons
☐	☒	A full description of the statistical parameters including central tendency (e.g. means) or other basic estimates (e.g. regression coefficient) AND variation (e.g. standard deviation) or associated estimates of uncertainty (e.g. confidence intervals)
☐	☒	For null hypothesis testing, the test statistic (e.g. F, t, r) with confidence intervals, effect sizes, degrees of freedom and P value noted<br>*Give P values as exact values whenever suitable.*
☒	☐	For Bayesian analysis, information on the choice of priors and Markov chain Monte Carlo settings
☒	☐	For hierarchical and complex designs, identification of the appropriate level for tests and full reporting of outcomes
☒	☐	Estimates of effect sizes (e.g. Cohen's d, Pearson's r), indicating how they were calculated

Our web collection on statistics for biologists contains articles on many of the points above.

Software and code

Policy information about availability of computer code

Data collection	GePulse acquisition software, Leica LAS X software, EPU 3.9, CellSens (Olympus)
Data analysis	Excel 2019, CryoSPARC v4, Phenix v1.20.1, Coot v0.9.8, ChimeraX, GraphPad Prism v9, Fiji, Ana custom analysis software, Sigmaplot V8.02, Cellpose v3.1.1.1, Scikit-image v0.22.0, Pandas v2.2.3, Numpy v1.26.4, Scipy v1.15.2, Statsmodels v0.14.2, Matplotlib v3.10.0, Seaborn v0.13.2

For manuscripts utilizing custom algorithms or software that are central to the research but not yet described in published literature, software must be made available to editors and reviewers. We strongly encourage code deposition in a community repository (e.g. GitHub). See the Nature Portfolio guidelines for submitting code & software for further information.

Data

Policy information about availability of data

All manuscripts must include a data availability statement. This statement should provide the following information, where applicable:

- Accession codes, unique identifiers, or web links for publicly available datasets
- A description of any restrictions on data availability
- For clinical datasets or third party data, please ensure that the statement adheres to our policy

The three-dimensional cryo-EM density maps have been deposited in the Electron Microscopy Data Bank under accession numbers EMD-55484 (LRRC8A-L402W), EMD-55483 (LRRC8A-L402W with ATP), EMD-57359(LRRC8A-I2C-L402W), EMD-55482 (LRRC8A-W168L-L402W) and EMD-55481 (LRRC8A-W168L-L402W channel trimer). The deposition includes maps of full-length proteins, corresponding half-maps 1 and 2, the mask used for final FSC calculation as well as relevant higher

resolution maps obtained after local refinement. Coordinates for the models of full-length LRRC8A-L402W, LRRC8A-L402W in presence of ATP , LRRC8A-I2C-L402W and LRRC8A-W168L-L402W have been deposited in the Protein Data Bank under accession numbers 9T35 [https://doi.org/9T35/pdb], 9T34 [https://doi.org/9T34/pdb], 29TC [https://doi.org/29TC/pdb] and 9T33 [https://doi.org/9T33/pdb], respectively. All other data needed to evaluate the conclusions of the study are present in the paper or in the supplementary materials. Source data are provided with this paper.

Research involving human participants, their data, or biological material

Policy information about studies with human participants or human data. See also policy information about sex, gender (identity/presentation), and sexual orientation and race, ethnicity and racism.

Reporting on sex and gender	n/a
Reporting on race, ethnicity, or other socially relevant groupings	n/a
Population characteristics	n/a
Recruitment	n/a
Ethics oversight	n/a

Note that full information on the approval of the study protocol must also be provided in the manuscript.

Field-specific reporting

Please select the one below that is the best fit for your research. If you are not sure, read the appropriate sections before making your selection.

☒ Life sciences ☐ Behavioural & social sciences ☐ Ecological, evolutionary & environmental sciences

For a reference copy of the document with all sections, see nature.com/documents/nr-reporting-summary-flat.pdf

Life sciences study design

All studies must disclose on these points even when the disclosure is negative.

Sample size	For two-electrode voltage-clamp (TEVC) recordings, data were obtained from 4–6 independent experiments.<br>For FRET experiments, the sample sizes (number of cells) were as follows:<br>8A-I2C: N = 57, 30, 21, 25 cells (for iso, 300, 250, and 200 mOsm conditions, respectively);<br>I2C-W168L: N = 60, 22, 30, 21 cells;<br>8A-I2C-L402W: N = 46, 30, 22, 19 cells;<br>8A-I2C-W168L-L402W: N = 51, 28, 23, 22 cells.<br>For FRET experiments under Apo and ligand (Sb1, Sb4) conditions:<br>8A-I2C: N = 39 (Apo), 25 (Sb1), 24 (Sb4);<br>8A-I2C-W168L: N = 40 (Apo), 24 (Sb1), 27 (Sb4);<br>8A-I2C-L402W: N = 37 (Apo), 28 (Sb1), 18 (Sb4);<br>8A-I2C-W168L-L402W: N = 41 (Apo), 17 (Sb1), 17 (Sb4).<br>For SE-FRET experiments:<br>8A: N = 6;<br>8A-I2C: N = 6.<br>For cell surface experiments:<br>N = 3 for all samples shown.<br>All data were collected from three independent experiments.
Data exclusions	n/a
Replication	All data were collected from three independent experiments.
Randomization	Samples were randomly assigned to experimental groups.
Blinding	Blinding was not applied

Reporting for specific materials, systems and methods

We require information from authors about some types of materials, experimental systems and methods used in many studies. Here, indicate whether each material, system or method listed is relevant to your study. If you are not sure if a list item applies to your research, read the appropriate section before selecting a response.

Materials & experimental systems

n/a	Involved in the study
☐	☒ Antibodies
☐	☒ Eukaryotic cell lines
☒	☐ Palaeontology and archaeology
☒	☐ Animals and other organisms
☒	☐ Clinical data
☒	☐ Dual use research of concern
☒	☐ Plants

Methods

n/a	Involved in the study
☒	☐ ChIP-seq
☒	☐ Flow cytometry
☒	☐ MRI-based neuroimaging

Antibodies

Antibodies used

Monoclonal Anti-LRRC8A antibody produced in mouse (Sigma-Aldrich, SAB1412855, dilute: 1:1000);
Peroxidase-AffiniPure Goat Anti-Mouse IgG (H+L) (min X Hu,Bov,Hrs,Rb,Sw Sr Prot) (Jackson Immuno Research, 115-035-146,dilute: 1:10000);
Rabbit Anti-LRRC8A (Voss et al., Science 2014; kindly provided by T. J. Jentsch, diluted 1:500);
Mouse Anti-GM130 (BD Biosciences, #610823, diluted 1:500);
Mouse Anti-Na+/K+ ATPase α-1 Antibody, clone C464.6 produced in mouse (Millipore, #05-369, diluted 1:500);
Mouse Anti-ß-actin (abcam, #ab8226, diluted 1:1000);
Goat Anti-Rabbit IgG H&L (HRP) (abcam, #6721, diluted 1:5000)
Goat Anti-Mouse IgG H&L (HRP) (abcam #6789, diluted 1:5000)

Validation

Monoclonal Anti-LRRC8A antibody was validated by Sigma-Aldrich for Western blot,
Rabbit Anti-LRRC8A was validated on LRRC8A knock out cell line for Western blot (Voss et al., Science 2014);
Monoclonal Anti-Na+/K+ ATPase α-1 Antibody, clone C464.6 was validated by Merck for Western blot,

Eukaryotic cell lines

Policy information about cell lines and Sex and Gender in Research

Cell line source(s)

Xenopus laevis, Leibniz Forschungsinstitut DSMZ, ATCC

Authentication

Oocytes from Xenopus laevis, HeLa cells from Leibniz Forschungsinstitut DSMZ, HEK293 cell line from ATCC

Mycoplasma contamination

The cell lines were tested and are free from mycoplasma contamination

Commonly misidentified lines
(See ICLAC register)

No commonly misidentified lines were used in the study

Plants

Seed stocks

n/a

Novel plant genotypes

n/a

Authentication

n/a
