# Supplementary material for: The intracellular subdomain of the volume-regulated anion channel subunit LRRC8A is a hotspot of channel activation
Source: Nat Commun. 2026 Sep 17;17:9885. doi: 10.1038/s41467-026-77507-y (PMC13586329; doi:10.1038/s41467-026-77507-y)
Supplement: Supplementary file 3 [file 41467_2026_77507_MOESM3_ESM.pdf]

nature portfolio

Peer Review File

The intracellular subdomain of the volume regulated anion channel subunit LRRC8A is a hotspot of channel activation

Corresponding Author: Professor Raimund Dutzler

This file contains all reviewer reports in order by version, followed by all author rebuttals in order by version.

Version 0:

Reviewer comments:

Reviewer #1

(Remarks to the Author)
The manuscript by Bertelli et al. addresses a fundamental question in the VRAC field: how conformational changes in the cytoplasmic LRR domains are coupled to pore opening. By focusing on the intracellular subdomain (ISD) of the obligatory LRRC8A subunit, the authors combine electrophysiology, FRET, and cryo-EM to dissect a critical structural element that links LRR domain dynamics to channel gating. The study provides compelling evidence that subtle perturbations within a hydrophobic pocket (V402 and W168) of the ISD can dramatically alter channel activity, including inducing constitutive opening. Overall, the work offers critical mechanistic insight into the activation of VRAC, a physiological important anion channel. Below are a few comments that should be addressed prior to publication.
1. The authors conclude that the mutant VRAC channel activities are insensitive to pharmacological inhibition. However, CBX (concentration not clearly specified) is neither a specific nor a particularly potent VRAC inhibitor. Notably, the observation that smaller currents remain sensitive to CBX inhibition (Fig. S1) suggests that the larger currents may simply require more effective blockade. The authors may therefore consider testing more potent VRAC inhibitors, such as high concentrations (>100 µM) of DCPIB or dicumarol, to more convincingly support the claim of pharmacological insensitivity. Another consideration is that most VRAC inhibitors are thought to act on the pore domain. Conceptually, it is therefore difficult to reconcile why these mutant channels—which primarily exhibit increased flexibility of the LRR domains but retain a largely similar pore architecture—would be insensitive to such blockers. Additional clarification or experimental support would help strengthen this interpretation.
2. On pages 124–125, it would be very helpful to directly compare the permeability to larger osmolytes (such as glutamate) between the L402W LRRC8A homomeric channel and the LRRC8A/8E heteromeric channel, to better clarify how this mutation affects substrate selectivity relative to heteromeric assemblies.
3. In Fig. 3e–h (FRET experiments with Sb1 and Sb4), it would be important to present the corresponding electrophysiological data showing the effects of these sybodies on the mutant channels, in order to directly correlate the observed conformational changes with functional outcomes. For example, does Sb1 inhibit L402W mutant? Does Sb4 potentiate W168L?
4. Optional: does W168L also compensate the hyperactive phenotype of L402A? It would further support the conclusion in Fig. 2.
5. In Fig. 5, it is unclear why the authors focused on the presumably nonphysiological trimeric complex rather than the canonical monomeric structure. The conformational changes the authors highlighted could simply due to this artificial assembly. It would be important to clarify the rationale for this choice and to present the monomeric structure and indicate if it exhibits similar conformational features.
6. Coloring of one hexamer in the top panel of Fig. 5a (map) is incorrect?
7. In Fig. 5c, it would be helpful to include a cryo-EM density map of the LRR domains viewed from the cytoplasmic (bottom) side in the supplementary figures, to better support the structural interpretation.

Reviewer #2

(Remarks to the Author)

In this paper, Bertelli and colleagues examine a series of mutations (based on disease-causing mutants in LRRC8C) along the ISD of LRRC8A that drastically alter the activation of the resultant homomeric A or heteromeric A/E VRAC channels. They utilize electrophysiology, FRET, and cryo-EM to provide a holistic view of the effect of these mutations on the structure

and function of the LRRC8A subunit and resultant VRAC channels. Overall, the study adds to the field of VRAC literature, but there are some specific critiques for the authors to address, which can be found below.

1. The authors state that "the fluorescent tag in one of the subunits induces a detectable, yet comparatively small constitutive activity, which is pronounced when substituting the 8A subunit but negligible in substitutions of 8E", and references Figure 1c, which depicts the current for 8A/8E-mCh. However, the sentence is confusing in that it appears to state that substituting a fluorescent tag into 8A results in constitutive activity without any mutations, but there is no figure to demonstrate that, nor any statement of subtracting said constitutive activity due to fluorescent tag from the 8A mutant homomeric channels. This should be clarified.
2. Line 120 incorrectly claims Fig 1h, as heteromeric A/E lack of response to hypotonicity, when in fact it is Fig 1g.
3. Is it possible that the mutant channels just express better on the plasma membrane? Its not such a trivial thing to know in oocytes but it seems important to be sure these are gating changes and not expression changes. It might be that the data in total is enough to convince readers that it is likely gating, but, at minimum, a mention of this is warranted.
4. The papers lacks a complete and clear description of statistical approaches
a. Its possible that I just cannot find it, but there does not appear to be a description of the statistics for the electrophysiology experiments. The authors provide statistical significance within their figures, but do not provide their statistical methods or numbers in the figure captions for Figures 1 or 2.
b. The authors state that 8A-I2C-L402W demonstrated increased FRET with the addition of the Sb1, but that is not statistically significant in their data so they cannot make that statement.
c. While the authors present some of their statistical methods in their caption for Figure 3, the paper would benefit from a data analysis and statistics section in the methods.

5. For the acceptor photobleaching FRET, what were the criteria for including cells in the analysis beyond the discussed? We have performed a great deal of acceptor photobleaching FRET and we found that a direct fusion of Cerulean and citrine (or CFP and YFP) shows a max FRET efficiency of around 25-30%. The data in Figures 3a-3d basically span the entire range of "full" FRET (25-30%) to almost no FRET (1-2%). That seems like a surprising variance that makes the data a little difficult to interpret especially given the relatively weak statistical significance. Could there be cells that maybe fail a potential exclusion criterion that are included here? Could it be that sometimes you do not have excess donor, which you want in acceptor photobleaching FRET, and sometimes you do? Or do you have another explanation for the spread in the data. The sybody data seems a little more consistent and I think is more readily interpretable but still has some significant variance.
a. Along these lines, do you worry about channels that are not on the surface altering your measured FRET since they might not experience the osmolarity change in the same way?
b. Do the cells change shape during swelling making the use of a mask challenging.
Any of these I suspect could alter the measured FRET which could create this sort of variance. Taken with the other data in the paper, this is not an essential result, but the data from 3A-3D is not overly convincing here.

Reviewer #3

(Remarks to the Author)
The manuscript by Bertelli and colleagues addresses the gating mechanism of the volume regulated anion channel LRRC8A. A protomer in the hexameric LRRC8 channel is composed of the pore domain (PD) and the regulatory domain LRRD. Conformational changes in the LRRDs are known to cause the channel to open. However, how the changes are transmitted to the PD is unknown. In this work, using a combination of electrophysiology, FRET and cryo-EM, the authors identified ISD, part of the PD, to be the segment responsible for this transmission process. By mimicking two disease-causing mutations, the authors demonstrated that a hydrophobic pocket in the ISD is essential for this functionality and mutations in the pocket allow the LRRDs to adopt various conformations. This discovery is novel and represents a major step in the full elucidation of the gating mechanism of LRRC8 channels. The experiments were done carefully and the manuscript is clearly written. I recommend its publication in Nature Communications with minor revision.

1. In the FRET experiments, how many donor- and acceptor-labeled subunits were there respectively per hexamer? If their stoichiometry or the geometric arrangements were not uniform, would that affect or complicate the interpretation of the FRET results?

2. It is mentioned in Line 355 that LRRDs may directly interact with the lipid membrane. Is it possible protein sample prepared in lipid nanodiscs may yield slightly different structures?

3. This reviewer feels that presentation of the double mutant structure in Fig 5 is not so clear. Some labels are too small, and the relative orientation between various panel parts is not clearly indicated. Perhaps, an SI figure at a larger scale can be added to show the structure better.

4. The final particle numbers in SI Table 1 differ from those listed in SI Figs 4 and 5.

5. Line 352: movement -> tilt or rotation.

6. Line 358: the reference needs to be cited.

7. SI Table 1: 7657 -> 7,657; all the B factors should be shown with the same number of digits after the decimal point.

Reviewer #4

(Remarks to the Author)
In this manuscript, Bertelli, Wang, Klüssendorf, and colleagues combine two-electrode voltage clamp electrophysiology, FRET-based conformational measurements, and cryo-EM to investigate the role of selected residues/mutations in the intracellular subdomain (ISD) of LRRC8A in channel activation. Guided by disease-causing variants previously identified in LRRC8C, the authors identify gain-of-function mutations in LRRC8A that lead to constitutive channel activity, increased LRRD mobility, and pronounced structural rearrangements within the ISD.

Overall, the study provides interesting insights into how mutations of three residues in the intracellular subdomain of LRRC8 channels affect channel activation. However, several aspects of the manuscript would benefit from improved framing and clarity, particularly regarding the broader biological relevance of the identified LRRC8A mutations and the presentation and interpretation of the functional data. While the technical execution of the structures appears to be strong (link to download maps and models is not functional), additional experiments, analysis and changes to the manuscript are needed to clarify the functional data and increase the relevance for the broad readership of Nature Communications.

Major Points
1. Framing and relevance of LRRC8A mutations

The conceptual entry point of the study is the presence of disease-causing variants in LRRC8C that map to the ISD, which motivates a mutational analysis of few selected residues in the corresponding region in LRRC8A. While this initial rationale seems reasonable, the manuscript would benefit from a stronger discussion of why this region of LRRC8A itself should be considered biologically or clinically relevant.

Furthermore, to support the main claim that the intracellular subdomain regulates the channel, additional experiments and ideally unbiased approaches (e.g. Ala scanning) are needed to obtain comprehensive mechanistic insights on the role of this subdomain and the relevance of different regions in the domain.

At present, it remains unclear whether the ISD of LRRC8A is subject to naturally occurring variation, selective constraint, or disease association in humans. As a result, the broader relevance of these gain-of-function mutations for understanding LRRC8A biology, physiology, or pathology is not fully established. Strengthening this connection, (e.g. through an analysis of available human genetic data for LRRC8A) would help contextualize the findings and broaden their significance for a general audience.

2. Presentation and clarity of electrophysiology data (Figure 1)

Figure 1 contains a substantial amount of electrophysiological data, and while the results support the authors' conclusions, the figure is challenging to digest in its current form. Consideration should be given to simplifying the presentation by placing representative traces or secondary datasets into supplementary figures while keeping the key summary data in the main figure to highlight the central conclusions.

The functional comparison of different mutations is challenging to interpret without ensuring that surface expression levels are similar for all the tested constructs. The authors should include an expression comparison to allow interpretation of the functional data with the conclusion that the effects are due to differences in the channel conformation/function rather than a consequence of having more or less channels at the membrane.

In addition, direct comparison between panels is difficult because the current–time plots and voltage-step traces are presented on different scales across panels. Using consistent axes, or explicitly indicating scale differences within the figure panels, would greatly improve readability.

It also requires careful reading of the figure legend to understand that the summary current values are extracted at $T = 0$ and $T \approx 280$ s. Explicitly marking these time points on the current–time traces or annotating the figure to indicate where summary values are taken would make this much clearer to the reader.

3. Variability in current amplitudes

The summary plots show considerable variability in current amplitudes at $T \approx 280$ s across multiple conditions. It would be helpful for the authors to clarify whether this variability primarily reflects differences in initial expression levels, variability in activation kinetics, or cell-to-cell differences in health. Providing additional information on starting current distributions, activation slopes, or exclusion criteria (if any) would help readers better interpret the spread in the data.

Related to this, including a schematic of the voltage-clamp protocol that indicates which portion of the protocol was used for quantification would improve clarity and reduce ambiguity about how the reported values were derived.

4. Rectification and ion selectivity of LRRC8A mutants

In Supplementary Figure 1c, the I–V relationships of the L402W mutant appear largely ohmic. Given that WT LRRC8A channels are typically reported to show pronounced rectification (PMID: 30775971), it would be informative to include comparable I–V data for WT LRRC8A under the same conditions. A direct comparison between WT and mutant channels, potentially including permeability ratios, would provide a clearer assessment of whether the activating mutations alter pore properties or selectivity in addition to gating.

5. Use of the I2C mutation in FRET experiments

The introduction of the I2C mutation to mitigate toxicity in the FRET experiments is clearly explained and technically justified. Nevertheless, this approach complicates direct comparison between the FRET data and the extensive electrophysiological characterization. The reduced dynamic range imposed by the I2C mutation makes it more difficult to fully appreciate the effects of the activating mutations on LRRD rearrangements. The authors should more explicitly discuss this limitation and should consider inducible or lower-expression systems as a way to alleviate toxicity.

Reviewer #5

(Remarks to the Author)
I co-reviewed this manuscript with one of the reviewers who provided the listed reports. This is part of the Nature Communications initiative to facilitate training in peer review and to provide appropriate recognition for Early Career Researchers who co-review manuscripts.

Reviewer #6

(Remarks to the Author)
I co-reviewed this manuscript with one of the reviewers who provided the listed reports. This is part of the Nature Communications initiative to facilitate training in peer review and to provide appropriate recognition for Early Career Researchers who co-review manuscripts.

Reviewer #7

(Remarks to the Author)
This manuscript studies the role of the intracellular subdomain (ISD) in regulating LRRC8 channel activity. LRRC8 proteins are activated by hypotonicity and serve to regulate cell volume. How LRRC8 proteins are activated is not completely understood. The authors demonstrate that mutations in the hydrophobic core of the ISD can lead to increased conductance or even constitutive activity (insensitivity towards hypotonicity; as observed for the L402W mutant). By probing the spatial dynamics of the cytoplasmic LRRDs, they confirm that LRRD mobility and activity remain correlated for the L402W ISD mutant. The structure of LRRC8A(L402W) demonstrate mutation-induced rearrangements in the ISD that in turn result in rearrangements near the membrane domain. In addition, the LRRDs appear highly flexible. The structure does not represent a conducting conformation though.
This study nicely combines functional, structural, and spatial dynamics (in a physiological context) to reveal the ISD as a key player in channel activation.

I only have minor comments
Line 110: can the stronger activating effect be explained by increased expression levels?
Line 113: indicate the phenotype of the respective truncation mutant
Line 244: discuss whether further tilting of the LRRD towards the membrane appears possible based on the structure. Is the channel known to be regulated by lipids/lipid head groups?

Version 1:

Reviewer comments:

Reviewer #1

(Remarks to the Author)
The authors have successfully addressed all my comments.

Reviewer #2

(Remarks to the Author)
Overall the authors responded well to comments and improved the manuscript. I think publication is now warranted.

Reviewer #3

(Remarks to the Author)

The authors have properly addressed all of my concerns regarding the manuscript, and I now recommend its publication.

Reviewer #4

(Remarks to the Author)
The authors have provided a well-executed biophysical characterization of LRRC8A mutations. However, I remain concerned about the limited scope of the study. Because the revision does not place these mutations within the context of human genetic variation or pathology, and lacks a systematic exploration of the intracellular domain, the physiological significance of the findings remains unclear. Furthermore, the broader implications of these data for homologous channels beyond LRRC8A are difficult to ascertain.

Reviewer #5

(Remarks to the Author)
I co-reviewed this manuscript with one of the reviewers who provided the listed reports. This is part of the Nature Communications initiative to facilitate training in peer review and to provide appropriate recognition for Early Career Researchers who co-review manuscripts.

Reviewer #6

(Remarks to the Author)
I co-reviewed this manuscript with one of the reviewers who provided the listed reports. This is part of the Nature Communications initiative to facilitate training in peer review and to provide appropriate recognition for Early Career Researchers who co-review manuscripts.

Open Access This Peer Review File is licensed under a Creative Commons Attribution 4.0 International License, which permits use, sharing, adaptation, distribution and reproduction in any medium or format, as long as you give appropriate credit to the original author(s) and the source, provide a link to the Creative Commons license, and indicate if changes were made.
In cases where reviewers are anonymous, credit should be given to 'Anonymous Referee' and the source.
The images or other third party material in this Peer Review File are included in the article's Creative Commons license, unless indicated otherwise in a credit line to the material. If material is not included in the article's Creative Commons license and your intended use is not permitted by statutory regulation or exceeds the permitted use, you will need to obtain permission directly from the copyright holder.
To view a copy of this license, visit https://creativecommons.org/licenses/by/4.0/

We thank the reviewers for their constructive comments, which we have considered in our revised manuscript and addressed in detail below.

In our revision we have included the following additional data:

- We have compared the expression of LRRC8A WT and of the strongly activating mutant L402W on the cell surface of X. laevis oocytes and HeLa cells and found in both cases similar levels.
- We have determined a structure of the double mutant I2C/L402W used for FRET analysis and found equivalent features as for L402W with respect to the conformational flexibility of the LRR domain and structural changes in the ISD.
- We have added additional electrophysiology data using the inhibitor DCPIB and measured permeability ratios comparing Cl^- with the larger anion glutamate.

Reviewer #1 (Remarks to the Author):

The manuscript by Bertelli et al. addresses a fundamental question in the VRAC field: how conformational changes in the cytoplasmic LRR domains are coupled to pore opening. By focusing on the intracellular subdomain (ISD) of the obligatory LRRC8A subunit, the authors combine electrophysiology, FRET, and cryo-EM to dissect a critical structural element that links LRR domain dynamics to channel gating. The study provides compelling evidence that subtle perturbations within a hydrophobic pocket (V402 and W168) of the ISD can dramatically alter channel activity, including inducing constitutive opening. Overall, the work offers critical mechanistic insight into the activation of VRAC, a physiological important anion channel.

We thank the reviewer for the constructive feedback and for recognizing the potential impact of this work.

Below are a few comments that should be addressed prior to publication.

1. The authors conclude that the mutant VRAC channel activities are insensitive to pharmacological inhibition. However, CBX (concentration not clearly specified) is neither a specific nor a particularly potent VRAC inhibitor. Notably, the observation that smaller currents remain sensitive to CBX inhibition (Fig. S1) suggests that the larger currents may simply require more effective blockade. The authors may therefore consider testing more potent VRAC inhibitors, such as high concentrations (>100 µM) of DCPIB or dicumarol, to more convincingly support the claim of pharmacological insensitivity. Another consideration is that most VRAC inhibitors are thought to act on the pore domain. Conceptually, it is therefore difficult to reconcile why these mutant channels—which primarily exhibit increased flexibility of the LRR domains but retain a largely similar pore architecture—would be insensitive to such blockers. Additional clarification or experimental support would help strengthen this interpretation.

We understand the concerns raised regarding the interpretation of the pharmacological data. It is important to note that the described effects of DCPIB and CBX on VRAC activity have been predominantly characterized for endogenous VRAC currents or heteromeric LRRC8 channels. In contrast, strongly effected channels analyzed here mainly concern homomeric LRRC8A assemblies (i.e. for the mutants LRRC8A-L402W and LRRC8A-L402A), which are known to display distinct pore properties and a different pharmacological profile compared to heteromeric VRAC, that is presumably related to its smaller pore size[1]. We also want to emphasize that the detailed inhibition mechanisms of the described molecules are still not resolved.

To address the referee's suggestion, we repeated the experiments for LRRC8A-L402W using DCPIB at a concentration of 200 µM and obtained results comparable to those observed with CBX, indicating that the mutant channels remain largely insensitive even to this more potent inhibitor.

We have added the DCPIB data as new panel (d) to Supplementary Fig. 1 and made the following changes to the manuscript, Line 123-126:

'As for the heteromeric A/E channels carrying the mutation, also these currents were insensitive to hypotonicity (Fig. 1g-i), hypertonicity (Supplementary Fig. 1b) and the VRAC inhibitors carbenoxolone (CBX) and DCPIB (Supplementary Fig. 1c, d).'

2. On pages 124–125, it would be very helpful to directly compare the permeability to larger osmolytes (such as glutamate) between the L402W LRRC8A homomeric channel and the LRRC8A/8E heteromeric channel, to better clarify how this mutation affects substrate selectivity relative to heteromeric assemblies.

As suggested by the reviewer, we performed permeability experiments using glutamate. The homomeric LRRC8A-L402W channel shows a reduced permeability to glutamate, accompanied by a larger shift in the reversal potential compared to the LRRC8A/8E heteromeric channel. This observation may be consistent with a more restrictive permeation pathway in the homomeric mutant, likely reflecting the smaller pore size imposed by the arginine ring.

We have added the data as panel f of Supplementary Fig. 1 and made the following changes to the manuscript, Line 128-130:

'The slightly less pronounced reduction in glutamate, could indicate a residual permeability of this amino acid (Supplementary Fig. 1e, f).'

3. In Fig. 3e–h (FRET experiments with Sb1 and Sb4), it would be important to present the corresponding electrophysiological data showing the effects of these sybodies on the mutant channels, in order to directly correlate the observed conformational changes with functional outcomes. For example, does Sb1 inhibit L402W mutant? Does Sb4 potentiate W168L?

We want to emphasize the role of the sybodies in this study as tools to either stabilize or induce conformational heterogeneity in the LRR-domains, which was observed on a structural level previously[2]. Nonetheless, the reviewer raises an interesting point. To address this, we expressed the L402W mutant either alone or together with Sb1 or Sb4. Under these conditions, Sb1 retained its inhibitory effect, whereas Sb4 did not further enhance the activity of the constitutively active LRRC8A-L402W mutant (Fig. R1, provided here for the reviewers). However, as several aspects of these experiments remain uncertain, including potential differences in expression levels, we refrain from drawing firm conclusions and have therefore not included these data in the manuscript.

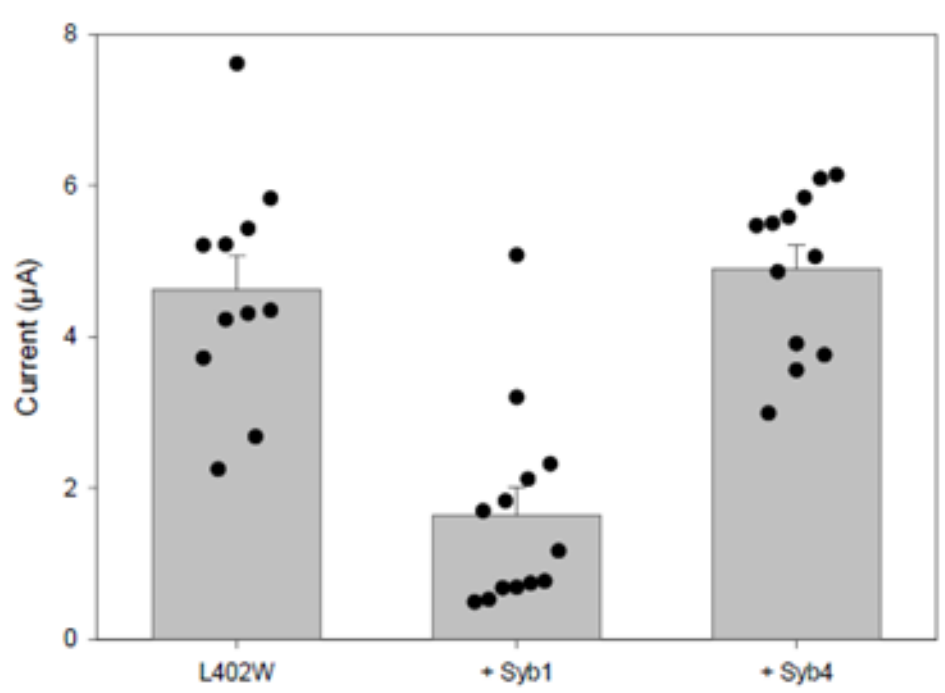

Fig. R1: Effects of sybodies Sb1 and Sb4 on LRRC8A-L402W-mediated currents in *Xenopus* oocytes.

4. Optional: does W168L also compensate the hyperactive phenotype of L402A? It would further support the conclusion in Fig. 2.

Since our data show that the effect of the double mutant W168L/L402W was not a mere compensation of the mutation, we think that this experiment would not be fully conclusive and thus decided not to include it.

5. In Fig. 5, it is unclear why the authors focused on the presumably nonphysiological trimeric complex rather than the canonical monomeric structure. The conformational changes the authors highlighted could simply due to this artificial assembly. It would be important to clarify the rationale for this choice and to present the monomeric structure and indicate if it exhibits similar conformational features.

While the trimer is clearly not relevant in a cellular context, it stabilizes a conformation of the LRRDs that is likely preexisting in solution. It does this by offering an interaction platform that shifts the equilibrium of a majority of the channels towards the trimeric state. Consequently, the

obtained densities are of higher resolution, particularly with respect to the LRRDs. We have decided to document this conformation in larger detail as it likely reflects the intermediate functional phenotype observed for the double mutant and illustrates that the effect is not a simple reversal to the WT conformation. Nevertheless, in the PD region, the structure is very similar to the conformation obtained from monomeric particles. In both cases the density of the LRRDs disappear upon further refinement steps as illustrated in Supplementary Fig. 8. We have now better emphasized this in our revised manuscript and added Supplementary Fig. 9, comparing densities obtained from monomeric and trimeric particle populations and illustrating conformational properties of the PD.

We have made the following changes to the manuscript, Line 323-331:

‘While this unique domain arrangement was stabilized in the trimeric channel assembly, we assume that it would also exist in isolated hexamers. However, in classes of such isolated hexamers, the domain conformation is more dynamic and less well defined in the density and thus difficult to assign with confidence (Fig. 5f, Supplementary Figs. 8b and 9c). Despite the ambiguity in the LRRD conformation, the equivalence of the structure of the PD in both particle populations is evident when comparing its density obtained from isolated particles (at 2.95 Å) with the density obtained upon masking one of the three channels, which was improved to 2.65 Å, where the density of the LRRDs have in both cases vanished (Supplementary Fig. 8f-g and 9d).’

6. Coloring of one hexamer in the top panel of Fig. 5a (map) is incorrect?

We have corrected the coloring in the figure panel.

7. In Fig. 5c, it would be helpful to include a cryo-EM density map of the LRR domains viewed from the cytoplasmic (bottom) side in the supplementary figures, to better support the structural interpretation.

We have added such density as panel a in Supplementary Fig. 9.

Reviewer #2 (Remarks to the Author):

In this paper, Bertelli and colleagues examine a series of mutations (based on disease-causing mutants in LRRC8C) along the ISD of LRRC8A that drastically alter the activation of the resultant homomeric A or heteromeric A/E VRAC channels. They utilize electrophysiology, FRET, and cryo-EM to provide a holistic view of the effect of these mutations on the structure and function of the LRRC8A subunit and resultant VRAC channels. Overall, the study adds to the field of VRAC

literature, but there are some specific critiques for the authors to address, which can be found below.

We thank the reviewer for these generally supportive comments.

1. The authors state that "the fluorescent tag in one of the subunits induces a detectable, yet comparatively small constitutive activity, which is pronounced when substituting the 8A subunit but negligible in substitutions of 8E", and references Figure 1c, which depicts the current for 8A/8E-mCh. However, the sentence is confusing in that it appears to state that substituting a fluorescent tag into 8A results in constitutive activity without any mutations, but there is no figure to demonstrate that, nor any statement of subtracting said constitutive activity due to fluorescent tag from the 8A mutant homomeric channels. This should be clarified.

We agree that in the previous version of the manuscript, the sentence might not have been sufficiently clear. Our intention was to point out that fluorescent tags are known to induce a small constitutive current when one of the two subunits within a heteromeric channel is tagged and a considerably larger current when both subunits are modified. This effect has been previously characterized in detail[3], where it was shown that the presence of a single fluorescent tag contributes similarly to a small constitutive current, regardless of whether the tag is inserted in the 8A or the 8E subunit.

In the present work we have chosen to tag only the LRRC8E subunit to avoid any additive contributions of the fluorescent tags to the already strong effect of the mutation. This strategy also ensures proper comparability between experimental conditions, as both control and mutant channels involve modifications of the same subunit. Tagging LRRC8A in all conditions would have complicated the interpretation of the mutant phenotype, particularly when directly assessing the effects of LRRC8A mutations. We have now revised the sentence to clarify this point and avoid misinterpretation.

We have made the following changes to the manuscript, Line 106-109:

'The fluorescent tag in the E subunit induces a detectable increase of the constitutive activity of the channel, which is, however, small compared with the effect observed when the fluorescent tag is present on both subunits (Fig. 1c). Consequently, we do not expect the presence of the fluorescent protein to strongly bias the results.'

2. Line 120 incorrectly claims Fig 1h, as heteromeric A/E lack of response to hypotonicity, when in fact it is Fig 1g.

We have corrected the mistake.

3. Is it possible that the mutant channels just express better on the plasma membrane? Its not such a trivial thing to know in oocytes but it seems important to be sure these are gating changes and not expression changes. It might be that the data in total is enough to convince readers that it is likely gating, but, at minimum, a mention of this is warranted.

To exclude the possibility that the increased currents observed for the mutant channels arise from altered plasma membrane expression rather than changes in gating, we have now performed additional experiments in both mammalian cells and *Xenopus laevis* oocytes.

In mammalian cells (HeLa), we assessed cell surface expression using two independent approaches: (i) surface biotinylation followed by quantitative Western blot analysis and (ii) immunostaining of an extracellular HA tag under non-permeabilizing conditions. Both assays consistently showed no detectable difference in plasma membrane expression between LRRC8A and LRRC8A-L402W. The data was included as new Supplementary Fig. 4, and we have made the following changes to the text, line 191-193:

'The effect could not be ascribed to increased cell surface expression of the mutants compared to LRRC8A-WT, as confirmed by surface biotinylation and immunostaining of an extracellular HA tag under non-permeabilizing conditions (Supplementary Fig. 4).'

In *Xenopus* oocytes, we compared fluorescence signals of LRRC8A and LRRC8A-L402W constructs and observed comparable expression levels at, or near the cell surface. The data was included as panel a in the revised Supplementary Fig. 1, and we have made the following changes to the text, line 119-123:

'The large currents of 8A-L402W/8E-mCh, which were observed already one day after mRNA injection, led us to express the mutant construct as homomeric assembly without 8E-mCh, resulting in similar large constitutive currents obtained in the same time range (Fig. 1h), with 8A-L402W expressed at similar levels as WT 8A (Supplementary Fig. 1a).'

Collectively, these experiments from independent approaches across two expression systems show no detectable differences in membrane expression between LRRC8A/8E-mCherry and LRRC8A-L402V/8E-mCherry channels, indicating that the enhanced currents are unlikely to result from increased surface expression.

4. The papers lacks a complete and clear description of statistical approaches a. Its possible that I just cannot find it, but there does not appear to be a description of the statistics for the electrophysiology experiments. The authors provide statistical significance within their figures, but do not provide their statistical methods or numbers in the figure captions for Figures 1 or 2. b.

We have added a description of the statistical methods used directly in the captions of Figures 1 and 2, including the number of experiments and the statistical tests applied.

The authors state that 8A-I2C-L402W demonstrated increased FRET with the addition of the Sb1, but that is not statistically significant in their data so they cannot make that statement.

As the observed increase in FRET does not reach statistical significance, we have revised the manuscript to clarify that Sb1 shows only a trend towards increased FRET in this mutant background rather than a significant effect.

We have introduced the following change to the manuscript, line 228-231:

‘While Sb1 showed a trend towards increased FRET in 8A-I2C-L402W and 8A-I2C-W168L-L402W, this effect was not statistically significant and did not reach levels observed with WT 8A-I2C upon addition of the same sybody (Fig. 3g, h).’

c. While the authors present some of their statistical methods in their caption for Figure 3, the paper would benefit from a data analysis and statistics section in the methods.

We have included a dedicated section in the Materials and Methods describing the data analysis procedures and statistical approaches used throughout the study for electrophysiology experiments (line 486-497). The statistical analysis of FRET data is provided in the legend of Fig. 3.

5. For the acceptor photobleaching FRET, what were the criteria for including cells in the analysis beyond the discussed? We have performed a great deal of acceptor photobleaching FRET and we found that a direct fusion of Cerulean and citrine (or CFP and YFP) shows a max FRET efficiency of around 25-30%. The data in Figures 3a-3d basically span the entire range of “full” FRET (25-30%) to almost no FRET (1-2%). That seems like a surprising variance that makes the data a little difficult to interpret especially given the relatively weak statistical significance. Could there be cells that maybe fail a potential exclusion criterion that are included here? Could it be that sometimes you do not have excess donor, which you want in acceptor photobleaching FRET, and sometimes you do? Or do you have another explanation for the spread in the data. The sybody data seems a little more consistent and I think is more readily interpretable but still has some significant variance.

We thank the reviewer for this thoughtful and important comment regarding the variability in FRET efficiencies and the potential influence of donor/acceptor ratios. We agree with the reviewer that

the donor/acceptor ratio can influence the measured FRET efficiency. We would like to clarify that, in acceptor photobleaching FRET experiments, an excess of acceptor relative to donor is generally favourable to ensure efficient energy transfer and accurate estimation of FRET efficiency.

Indeed, when plotting FRET efficiency against the Cerulean/Venus (DD/AA) signal ratio (as a proxy for relative expression levels), we observe a moderate correlation, with higher acceptor levels tending to yield higher FRET efficiencies (Fig. R2a, shown here for the reviewers and editor). However, this relationship is consistent across all LRRC8A variants and all osmolarities, and therefore does not account for the differences observed between genotypes.

Importantly, applying a threshold to exclude cells with high donor/acceptor ratios (e.g., DD/AA > 0.3) does not alter the overall results or conclusions, either for the baseline measurements (Fig. 3b-c) or in the presence of sybodies (Fig. R3, shown here for the reviewers and editor). We therefore chose an unbiased approach and included all cells in the analysis rather than introducing an arbitrary cutoff.

Regarding the overall spread in FRET efficiencies, we note that LRRC8A complexes are expected to exhibit conformational flexibility, particularly in the C-terminal LRR domains, which could contribute to variability in inter-subunit distances and thus FRET efficiency. In line with this interpretation, the reviewer correctly notes that the presence of sybodies reduces variability; this is consistent with their proposed role in stabilizing defined conformational states.

Together, these analyses indicate that while donor/acceptor ratios contribute modestly to variability, they do not explain the observed differences between constructs, nor do they affect the conclusions of our study.

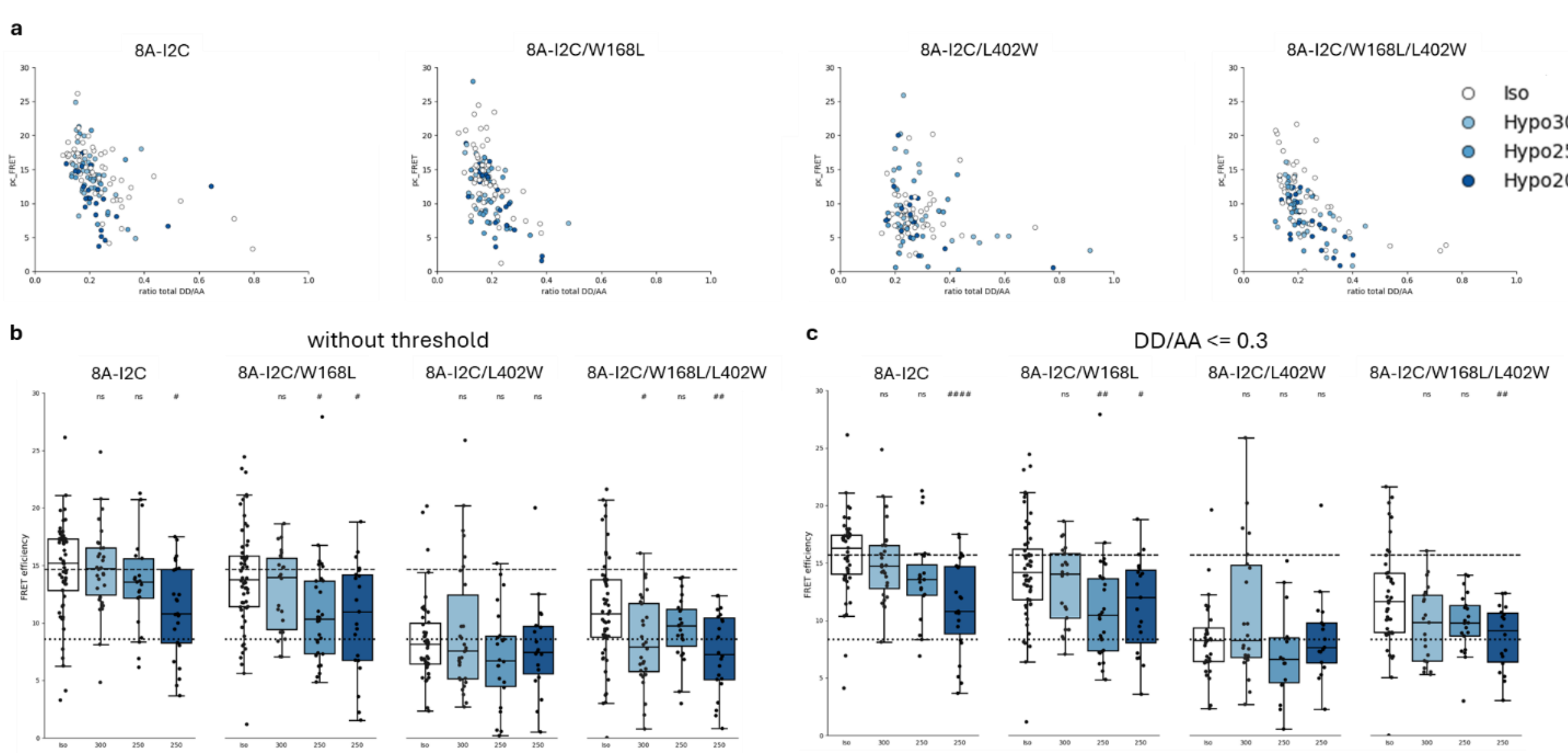

Fig. R2: Dependence of FRET efficiency in acceptor photo-bleaching experiments on donor/acceptor ratio. **a**, FRET efficiency plotted against the ratio of donor/acceptor fluorescence signal corresponding to LRRC8A-mCerulean3/LRRC8A-mVenus expression ratio for the four

LRRC8A variants as depicted. Shades of blue indicate osmolarity. **b-c**, Summary of the results, **b**, including all cells as in the manuscript or, **c**, excluding cells with a donor/acceptor ratio above 0.3.

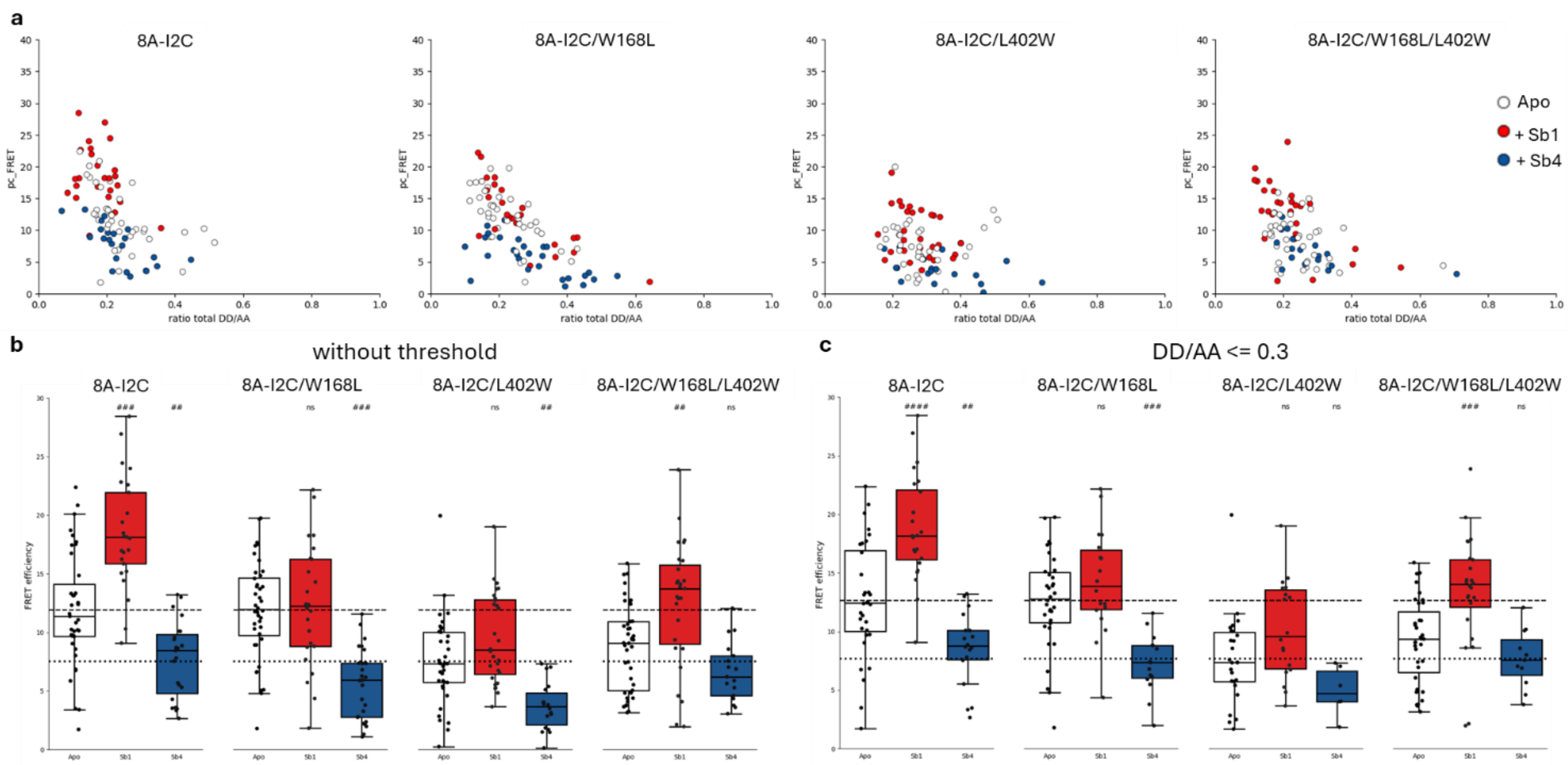

Fig. R3: Dependence of FRET efficiency in sybody experiments on donor/acceptor ratio. **a**, FRET efficiency plotted against the ratio of donor/acceptor fluorescence signal corresponding to LRRC8A-mCerulean3/LRRC8A-mVenus expression ratio for the four LRRC8A variants as depicted. Color code depicts coexpression with sybodies. **b-c**, Summary of the results, **b**, including all cells as in the manuscript or, **c**, excluding cells with a donor/acceptor ratio above 0.3.

a. Along these lines, do you worry about channels that are not on the surface altering your measured FRET since they might not experience the osmolarity change in the same way?

We agree that intracellular pools of the channel will contribute to the measured FRET signal and might not respond to osmotic changes in the same way as channels at the plasma membrane. However, as now demonstrated by our additional experiments (surface biotinylation and immunostaining of an extracellular HA tag under non-permeabilizing conditions; see response to comment 3 and Supplementary Fig. 4), all constructs exhibit comparable levels of plasma membrane expression. Therefore, any contribution from intracellular channels would be expected to affect all variants similarly and cannot explain the observed differences between LRRC8A constructs. In addition, our HA immunostaining indicates that plasma membrane and intracellular pools cannot be reliably separated by simple masking approaches, due to overlapping fluorescence signals, further supporting our use of a whole-cell analysis.

b. Do the cells change shape during swelling making the use of a mask challenging. Any of these I suspect could alter the measured FRET which could create this sort of variance. Taken with the other data in the paper, this is not an essential result, but the data from 3A-3D is not overly convincing here.

We did not observe substantial distortions that would compromise segmentation or mask-based analysis, which might be relevant for the SE-FRET experiments. In addition, the acceptor photobleaching FRET approach relies on intensity changes within the same cell before and after bleaching, making it relatively robust to moderate changes in cell morphology.

More generally, we acknowledge that factors such as expression variability, subcellular localization, and conformational flexibility may contribute to the spread in FRET efficiencies. As discussed above, additional analyses (including donor/acceptor ratio filtering) do not alter the conclusions. Furthermore, the reduced variance observed in the presence of sybodies is consistent with their stabilizing effect on channel conformation, supporting a biological origin of the variability.

Taken together, while we agree that the variability limits the quantitative interpretability of individual FRET values, the overall trends are consistent across conditions and supported by independent lines of evidence.

Reviewer #3 (Remarks to the Author):

The manuscript by Bertelli and colleagues addresses the gating mechanism of the volume regulated anion channel LRRC8A. A protomer in the hexameric LRRC8 channel is composed of the pore domain (PD) and the regulatory domain LRRD. Conformational changes in the LRRDs are known to cause the channel to open. However, how the changes are transmitted to the PD is unknown. In this work, using a combination of electrophysiology, FRET and cryo-EM, the authors identified ISD, part of the PD, to be the segment responsible for this transmission process. By mimicking two disease-causing mutations, the authors demonstrated that a hydrophobic pocket in the ISD is essential for this functionality and mutations in the pocket allow the LRRDs to adopt various conformations. This discovery is novel and represents a major step in the full elucidation of the gating mechanism of LRRC8 channels. The experiments were done carefully and the manuscript is clearly written. I recommend its publication in Nature Communications with minor revision.

We thank the reviewer for the supporting comments.

1. In the FRET experiments, how many donor- and acceptor-labeled subunits were there respectively per hexamer? If their stoichiometry or the geometric arrangements were not uniform, would that affect or complicate the interpretation of the FRET results?

In our experiments, donor- and acceptor-labelled LRRC8A subunits were co-expressed at a nominal 1:1 ratio using identical constructs differing only in the fluorescent tag. We therefore expect that, on average, donor and acceptor subunits are present at similar levels within the cell, although some variability between individual cells is unavoidable. Given the hexameric assembly of LRRC8A channels, this expression scheme is expected to yield complexes that most frequently contain approximately three donor- and three acceptor-labelled subunits, while other stoichiometries (e.g., 2:4 or 4:2) will also occur. Likewise, the geometric arrangement of donor and acceptor subunits within individual hexamers is not controlled and will vary.

We agree that such heterogeneity can contribute to the spread in measured FRET efficiencies. However, these parameters are determined by stochastic assembly and expression and are therefore expected to be similar across all constructs and experimental conditions (see also our answer to Reviewer 2, point 5). As a result, they would primarily introduce variability that averages out across the population, rather than systematically biasing one construct relative to another. Importantly, the consistent differences observed between LRRC8A variants, as well as the reduced variability upon addition of sybodies (which are expected to constrain conformational flexibility), support the interpretation that the measured FRET changes reflect underlying differences in channel conformation rather than differences in subunit stoichiometry or arrangement.

2. It is mentioned in Line 355 that LRRDs may directly interact with the lipid membrane. Is it possible protein sample prepared in lipid nanodiscs may yield slightly different structures?

Please note that the proposal of a potential interaction with the membrane is at this stage speculative. Previous structures of LRRC8A in lipid nanodiscs[4] have shown generally similar structural properties of the TMD but an even larger structural heterogeneity of the LRRDs. Additionally, the membrane area provided by a small nanodisc might not be sufficiently large to allow for interactions with tilted LRRDs. We thus hope to be able to address this question in structures of the protein in native vesicles in future studies.

3. This reviewer feels that presentation of the double mutant structure in Fig 5 is not so clear. Some labels are too small, and the relative orientation between various panel parts is not clearly indicated. Perhaps, an SI figure at a larger scale can be added to show the structure better.

We have increased labels in Fig. 5 and tried to make clearer assignment of subunits in the trimeric channel assembly. We have also introduced Supplementary Fig. 9 to better illustrate the LRRD domain density in different channel reconstructions.

4. The final particle numbers in SI Table 1 differ from those listed in SI Figs 4 and 5.

We have corrected this mistake.

5. Line 352: movement -> tilt or rotation.

We have corrected this sentence to make clear that we are talking about a tilt of the LRRD.

6. Line 358: the reference needs to be cited.

We have included the reference.

7. SI Table 1: 7657 -> 7,657; all the B factors should be shown with the same number of digits after the decimal point.

We have introduced this correction.

Reviewer #4 (Remarks to the Author):

In this manuscript, Bertelli, Wang, Klüssendorf, and colleagues combine two-electrode voltage clamp electrophysiology, FRET-based conformational measurements, and cryo-EM to investigate the role of selected residues/mutations in the intracellular subdomain (ISD) of LRRC8A in channel activation. Guided by disease-causing variants previously identified in LRRC8C, the authors identify gain-of-function mutations in LRRC8A that lead to constitutive channel activity, increased LRRD mobility, and pronounced structural rearrangements within the ISD.

Overall, the study provides interesting insights into how mutations of three residues in the intracellular subdomain of LRRC8 channels affect channel activation. However, several aspects of the manuscript would benefit from improved framing and clarity, particularly regarding the broader biological relevance of the identified LRRC8A mutations and the presentation and interpretation of the functional data. While the technical execution of the structures appears to be strong (link to download maps and models is not functional), additional experiments, analysis and changes to the manuscript are needed to clarify the functional data and increase the relevance for the broad readership of Nature Communications.

We thank the reviewer for the supporting comments and take the remarks concerning clarity serious. Most of these comments are addressed in our revised manuscript.

Major Points

1. Framing and relevance of LRRC8A mutations

The conceptual entry point of the study is the presence of disease-causing variants in LRRC8C that map to the ISD, which motivates a mutational analysis of few selected residues in the corresponding region in LRRC8A. While this initial rationale seems reasonable, the manuscript would benefit from a stronger discussion of why this region of LRRC8A itself should be considered biologically or clinically relevant. Furthermore, to support the main claim that the intracellular subdomain regulates the channel, additional experiments and ideally unbiased approaches (e.g. Ala scanning) are needed to obtain comprehensive mechanistic insights on the role of this subdomain and the relevance of different regions in the domain.

At present, it remains unclear whether the ISD of LRRC8A is subject to naturally occurring variation, selective constraint, or disease association in humans. As a result, the broader relevance of these gain-of-function mutations for understanding LRRC8A biology, physiology, or pathology is not fully established. Strengthening this connection, (e.g. through an analysis of available human genetic data for LRRC8A) would help contextualize the findings and broaden their significance for a general audience.

We thank the referee for these comments and for raising important points regarding the framing and relevance of the intracellular subdomain (ISD) of LRRC8A. The primary objective of this study was to investigate a specific mechanistic aspect of VRAC function, namely the interaction between particular residues within the ISD that influence channel gating and the way how conformational rearrangements of the cytosolic LRR domains are coupled to pore opening in VRAC. This work was motivated by disease-associated variants in LRRC8C[5], which guided the selection of the corresponding residues in LRRC8A for functional analysis. Our focus was therefore targeted and hypothesis-driven, and not intended as an exhaustive exploration of the ISD.

Regarding the suggestion to employ unbiased approaches such as alanine scanning, we agree that such strategies could yield broader insight into the functional landscape of the ISD. However, a systematic mutational analysis of this region would constitute a substantial study on its own and lies beyond the scope of the present work. Importantly, preliminary data indicate that the observed effects are highly residue-specific with mutations at positions neighboring W168 (e.g., residues 167 or 169) not showing the same functional phenotype. This highlights the specific role of the studied residues while not excluding that other positions in the ISD could also be functionally relevant. Since the data on other positions is at that stage preliminary and not systematic, we decided to not include these additional mutants and instead add them in a future systematic investigation.

We also acknowledge the referee's point regarding naturally occurring variation or disease association in the ISD of LRRC8A. While we agree that this represents an interesting and potentially important avenue for future investigation, an analysis of human genetic variation in LRRC8A was outside the scope of the present study. It should also be emphasized that currently there are no credible associations of LRRC8A mutants with disease. In fact, since the constitutively open mutant of the ubiquitously expressed LRRC8A seems toxic to cells (i.e. we had to combine it with the inhibitory I2C mutation for live-cell experiments and found reduced total expression levels in our newly added biotinylation experiments in HeLa cells, Supplementary Fig. 4c), we would expect such mutations to be lethal and thus not be found in patients. We therefore deliberately refrained from speculative discussion on clinical relevance beyond what is directly supported by our experimental findings.

In summary, our conclusions are strictly based on the evidence presented: we identify a specific structural interaction within the ISD of LRRC8A that is essential for coupling LRRD conformational changes to channel gating. The disruption of this interaction strongly alters channel function, demonstrating that this region acts as a critical coupling element, without making broader claims about domain-wide functionality or disease relevance.

2. Presentation and clarity of electrophysiology data (Figure 1)

Figure 1 contains a substantial amount of electrophysiological data, and while the results support the authors' conclusions, the figure is challenging to digest in its current form. Consideration should be given to simplifying the presentation by placing representative traces or secondary datasets into supplementary figures while keeping the key summary data in the main figure to highlight the central conclusions.

While we agree that Fig. 1 is quite dense, we think that the traces are helpful to better appreciate differences in the current magnitude and activation properties. We thus decided against moving the traces to the supplementary materials.

The functional comparison of different mutations is challenging to interpret without ensuring that surface expression levels are similar for all the tested constructs. The authors should include an expression comparison to allow interpretation of the functional data with the conclusion that the effects are due to differences in the channel conformation/function rather than a consequence of having more or less channels at the membrane.

We appreciate the referee's suggestion and agree that it is essential to ensure that the observed functional effects are not simply due to differences in plasma membrane expression. To address this, we performed additional experiments in both mammalian cells and *Xenopus laevis* oocytes (see also our answer to Reviewer 2, point 3). These analyses revealed no detectable differences

in surface expression between wild-type LRRC8A and mutant LRRC8A-L402W in oocytes, indicating that the enhanced currents result from altered channel gating rather than changes in abundance at the plasma membrane.

We have included these novel data as new Supplementary Fig. 1a (A-VFP fluorescence quantification in oocytes) and Supplementary Fig. 4 (cell surface biotinylation of LRRC8A variants expressed in HeLa cells and cell surface expression of LRRC8A variants assessed by HA immunostaining without cell permeabilization) and made changes in the manuscript that were described in the response to Reviewer 2 point 3.

In addition, direct comparison between panels is difficult because the current–time plots and voltage-step traces are presented on different scales across panels. Using consistent axes, or explicitly indicating scale differences within the figure panels, would greatly improve readability.

The current traces represent data from representative recordings where we have chosen different scales to more clearly represent the current evolution over time for different mutants. A comparison of the current magnitudes of the entire dataset is displayed in the bar-graphs in Fig. 1f, I, n.

It also requires careful reading of the figure legend to understand that the summary current values are extracted at T = 0 and T ≈ 280 s. Explicitly marking these time points on the current–time traces or annotating the figure to indicate where summary values are taken would make this much clearer to the reader.

As suggested, we have revised the figure legends to make them more descriptive and to improve readability, clearly indicating what is shown in each panel. In particular, we now explicitly state that the summary current values correspond to measurements taken at *t* = 0 and after ~280 s of hypotonic stimulation (towards the end of the displayed recordings). To further clarify this point, we have added a detailed description of how these values were extracted in the Materials and Methods section (Statistical analysis of electrophysiology data, line 486-497). There, we now specify the timing of solution exchange and the time points used for quantification during the time-course recordings.

3. Variability in current amplitudes

The summary plots show considerable variability in current amplitudes at T ≈ 280 s across multiple conditions. It would be helpful for the authors to clarify whether this variability primarily reflects

differences in initial expression levels, variability in activation kinetics, or cell-to-cell differences in health. Providing additional information on starting current distributions, activation slopes, or exclusion criteria (if any) would help readers better interpret the spread in the data.

Related to this, including a schematic of the voltage-clamp protocol that indicates which portion of the protocol was used for quantification would improve clarity and reduce ambiguity about how the reported values were derived.

Variability in current amplitudes across oocytes is expected, reflecting inherent differences in expression levels that can fluctuate between experimental batches and from day to day. This variability is unavoidable under our experimental conditions; however, it is reasonably assumed to be evenly distributed across all experimental groups. Regarding the quantification, we did not rely on full current-voltage relationships. Instead, summary values were derived from the average current during a 50-ms pulse at 60 mV, applied every 5 seconds in the time-course experiments. For clarity, we have now included a short description of this approach in the Materials and Methods section.

Line 468-470:

'To monitor the activation kinetics during hypotonic stimulation, oocytes were clamped at 0 mV interrupted by 200 ms pulses to +60 mV in 5 s intervals, with averaged currents over the pulse period plotted as a function of time.'

4. Rectification and ion selectivity of LRRC8A mutants

In Supplementary Figure 1c, the I–V relationships of the L402W mutant appear largely ohmic. Given that WT LRRC8A channels are typically reported to show pronounced rectification (PMID: 30775971), it would be informative to include comparable I–V data for WT LRRC8A under the same conditions. A direct comparison between WT and mutant channels, potentially including permeability ratios, would provide a clearer assessment of whether the activating mutations alter pore properties or selectivity in addition to gating.

We thank the reviewer for this suggestion. Unfortunately, a direct comparison with wild-type LRRC8A currents is not feasible in our experimental system. It is well established in the literature that LRRC8A expressed alone does not produce robust macroscopic currents in cellular contexts such as cell lines or oocytes and therefore cannot be reliably characterized by conventional whole-cell electrophysiology upon cell swelling. Functional VRAC currents typically require heteromeric assemblies containing LRRC8A together with additional LRRC8 subunits (e.g., LRRC8C/D/E), whereas LRRC8A homomers generally exhibit very low activity and fail to recapitulate native VRAC properties. LRRC8A homomers have been characterized by electrophysiology only at very low intracellular ionic strength[1,6] or in artificial reconstitution systems[4,7], which differ from the recordings shown here. Our study focuses on constitutively active

LRRC8A mutants that allow robust electrophysiological characterization of channel behavior. Importantly, the primary goal of this work is to investigate the activation mechanism of these mutants rather than to perform a detailed analysis of ion selectivity or permeability changes. While such measurements could be informative, they fall outside the scope of the present study.

5. Use of the I2C mutation in FRET experiments

The introduction of the I2C mutation to mitigate toxicity in the FRET experiments is clearly explained and technically justified. Nevertheless, this approach complicates direct comparison between the FRET data and the extensive electrophysiological characterization. The reduced dynamic range imposed by the I2C mutation makes it more difficult to fully appreciate the effects of the activating mutations on LRRD rearrangements. The authors should more explicitly discuss this limitation and should consider inducible or lower-expression systems as a way to alleviate toxicity.

While we observed a similar reduction in SE-FRET upon hypotonic treatment with the I2C mutation compared to WT LRRC8A (Supplementary Fig. 3d), the reviewer raises an important point. To investigate whether the introduction of the mutation I2C would interfere with the conformational properties of the LRRDs in the background of the mutation L402W, we have determined the cryo-EM structure of the double mutant 8A-I2C-L402W where we found very similar structural properties as in the single mutant 8A-L402W, including the strong tilt of the LRRD in 2D and 3D classes at low resolution and the same rearrangement of the ISD in the refined map at 2.5 Å, where the LRRDs have averaged out. These data illustrate that the introduction of I2C did not alter the general structural properties of the mutant and that its use for FRET experiments is fully justified. The presence of a pronounced band of a dimer of the 8A-I2C-L402W mutant detected in a SDS PAGE gel under non-reducing conditions, suggest the formation of a disulfide bridge between the disordered N-termini of the subunits as plausible mechanism of its inability to conduct ions.

We have included this structure in our revised manuscript (Supplementary Figs. 6 and 7) and made the following changes to the text, line 282-299:

‘To clarify whether similar structural properties would also be observed in the construct used for FRET studies, we have determined the structure of the double mutant 8A-I2C-L402W that combines the strongly activating L402W with a mutation on the N-terminus exerting the opposite effect to demonstrate that the inhibitory mutation did not perturb the structural rearrangements of the former (Supplementary Fig. 6, Supplementary Table 1). In this dataset, we found equivalent strong distortions in the conformation of the LRRDs in low resolution reconstructions as observed in the data of LRRC8A-L402W (Supplementary Figs. 5b and 6c). As in case of the single mutant, the LRRD density vanishes in the high-resolution reconstruction at 2.5 Å (Supplementary Fig 6f), whereas the ISD shows the same conformational rearrangement leading to the dissociation of the terminal helix CTH3, which is not defined in the density (Supplementary Fig. 7). Similarly, we did not find pronounced density for the N-terminus, which remains disordered. Instead, we found

the appearance of a crosslink leading to the formation of dimers on SDS-PAGE gels under non-reducing conditions that was not observed in the single mutant (Supplementary Fig. 6b), which suggests that the introduction of a cysteine residue leads to the formation of inter-subunit disulfide bridges, which could block the pore at the region of the N-terminus. Consequently, this data confirms that the mutation of sites in the ISD, while essential in the transduction of a conformational change leading to pore opening, acts independently of the actual gate of the pore that regulates the pore conformation. ‘

Reviewer #7 (Remarks to the Author):

This manuscript studies the role of the intracellular subdomain (ISD) in regulating LRRC8 channel activity. LRRC8 proteins are activated by hypotonicity and serve to regulate cell volume. How LRRC8 proteins are activated is not completely understood. The authors demonstrate that mutations in the hydrophobic core of the ISD can lead to increased conductance or even constitutive activity (insensitivity towards hypotonicity; as observed for the L402W mutant). By probing the spatial dynamics of the cytoplasmic LRRDs, they confirm that LRRD mobility and activity remain correlated for the L402W ISD mutant. The structure of LRRC8A(L402W) demonstrate mutation-induced rearrangements in the ISD that in turn result in rearrangements near the membrane domain. In addition, the LRRDs appear highly flexible. The structure does not represent a conducting conformation though.

This study nicely combines functional, structural, and spatial dynamics (in a physiological context) to reveal the ISD as a key player in channel activation.

We thank the reviewer for the supportive comments.

I only have minor comments:

Line 110: can the stronger activating effect be explained by increased expression levels?

To address whether the stronger activating effect could be explained by increased expression levels, we examined this explicitly using the strongly activating mutant L402W as a representative example. Using multiple independent approaches, including surface biotinylation and immunostaining of an extracellular HA tag under non-permeabilizing conditions in mammalian cells, as well as fluorescence-based assessment in *Xenopus laevis* oocytes, we did not detect increased plasma membrane expression compared to WT LRRC8A.

These results indicate that the enhanced activity is unlikely to arise from increased expression levels. A detailed description of these experiments and their outcomes is provided in our responses to Reviewer 2 (point 3) and Reviewer 4 (point 2), where this issue is addressed more extensively.

Line 113: indicate the phenotype of the respective truncation mutant

We have made the following changes to indicate the functional phenotype (line 114-116):

'We next constructed the mutant 8A-L402W (Fig. 1a), inspired by the second disease-related variant of 8C that carries a frameshift mutation at the corresponding position, resulting in a subunit lacking the LRRD. In 8C, this variant also strongly enhances activation. '

Line 244: discuss whether further tilting of the LRRD towards the membrane appears possible based on the structure. Is the channel known to be regulated by lipids/lipid head groups?

Although we regard the question as clearly interesting, we want to emphasize that the assignment of the detailed conformation of the LRRD in the mutant L402W is prohibited by the low resolution of the cryo-EM density in this region as a consequence of the high mobility of the domain. While it appears that in the observed conformation the domain could get close to the membrane surface, the interaction with membrane lipids remains uncertain and a potential regulation by lipid head groups has so far not been described.

References

1 Yamada, T., Figueroa, E. E., Denton, J. S. & Strange, K. LRRC8A homohexameric channels poorly recapitulate VRAC regulation and pharmacology. *Am J Physiol Cell Physiol* **320**, C293–C303 (2021). https://doi.org/10.1152/ajpcell.00454.2020

2 Deneka, D. *et al.* Allosteric modulation of LRRC8 channels by targeting their cytoplasmic domains. *Nat Commun* **12**, 5435 (2021). https://doi.org/10.1038/s41467-021-25742-w

3 Gaitán-Peñas, H. *et al.* Investigation of LRRC8-Mediated Volume-Regulated Anion Currents in Xenopus Oocytes. *Biophys J* **111**, 1429–1443 (2016). https://doi.org/10.1016/j.bpj.2016.08.030

4 Kern, D. M., Oh, S., Hite, R. K. & Brohawn, S. G. Cryo-EM structures of the DCPIB-inhibited volume-regulated anion channel LRRC8A in lipid nanodiscs. *Elife* **8**, e42636 (2019). https://doi.org/10.7554/eLife.42636

5 Quinodoz, M. *et al.* De novo variants in LRRC8C resulting in constitutive channel activation cause a human multisystem disorder. *EMBO J* **44**, 413–436 (2025). https://doi.org/10.1038/s44318-024-00322-y

6 Deneka, D., Sawicka, M., Lam, A. K. M., Paulino, C. & Dutzler, R. Structure of a volume-regulated anion channel of the LRRC8 family. *Nature* **558**, 254–259 (2018). https://doi.org/10.1038/s41586-018-0134-y

7 Syeda, R. *et al.* LRRC8 Proteins Form Volume-Regulated Anion Channels that Sense Ionic Strength. *Cell* **164**, 499–511 (2016). https://doi.org/10.1016/j.cell.2015.12.031

REVIEWER COMMENTS

Reviewer #4 (Remarks to the Author):

The authors have provided a well-executed biophysical characterization of LRRC8A mutations. However, I remain concerned about the limited scope of the study. Because the revision does not place these mutations within the context of human genetic variation or pathology, and lacks a systematic exploration of the intracellular domain, the physiological significance of the findings remains unclear. Furthermore, the broader implications of these data for homologous channels beyond LRRC8A are difficult to ascertain.

As mentioned before, there were so far no credible disease-related mutations on LRRC8A in this region identified, most likely since such strongly activating mutations would be lethal. Even for the equivalent disease-related mutations in the less abundant subunit LRRC8C, there were only few patients identified. A systematic investigation of this region was beyond the scope of this study and will be part of future investigations.
